# Poor statistical reporting, inadequate data presentation and spin persist despite editorial advice

**DOI:** 10.1371/journal.pone.0202121

**Published:** 2018-08-15

**Authors:** Joanna Diong, Annie A. Butler, Simon C. Gandevia, Martin E. Héroux

**Affiliations:** 1 Sydney Medical School, University of Sydney, Sydney, NSW, Australia; 2 Neuroscience Research Australia (NeuRA), Randwick, NSW, Australia; 3 University of New South Wales, Randwick, NSW, Australia; Van Andel Institute, UNITED STATES

## Abstract

The Journal of Physiology and British Journal of Pharmacology jointly published an editorial series in 2011 to improve standards in statistical reporting and data analysis. It is not known whether reporting practices changed in response to the editorial advice. We conducted a cross-sectional analysis of reporting practices in a random sample of research papers published in these journals before (n = 202) and after (n = 199) publication of the editorial advice. Descriptive data are presented. There was no evidence that reporting practices improved following publication of the editorial advice. Overall, 76-84% of papers with written measures that summarized data variability used standard errors of the mean, and 90-96% of papers did not report exact p-values for primary analyses and post-hoc tests. 76-84% of papers that plotted measures to summarize data variability used standard errors of the mean, and only 2-4% of papers plotted raw data used to calculate variability. Of papers that reported p-values between 0.05 and 0.1, 56-63% interpreted these as trends or statistically significant. Implied or gross spin was noted incidentally in papers before (n = 10) and after (n = 9) the editorial advice was published. Overall, poor statistical reporting, inadequate data presentation and spin were present before and after the editorial advice was published. While the scientific community continues to implement strategies for improving reporting practices, our results indicate stronger incentives or enforcements are needed.

## Introduction

The accurate communication of scientific discovery depends on transparent reporting of methods and results. Specifically, information on data variability and results of statistical analyses are required to make accurate inferences.

The quality of statistical reporting and data presentation in scientific papers is generally poor. For example, one third of clinical trials in molecular drug interventions and breast cancer selectively report outcomes [1], 60-95% of biomedical research papers report statistical analyses that are not pre-specified or are different to published analysis plans [2], and one third of all graphs published in the prestigious Journal of the American Medical Association cannot be interpreted unambiguously [3]. In addition, reported results may differ from the actual statistical results. For example, distorted interpretation of statistically non-siginficant results (i.e. spin) is present in more than 40% of clinical trial reports [4].

Many reporting guidelines (e.g. the Consolidated Standards of Reporting Trials; CONSORT [5]) have been developed, endorsed and mandated by key journals to improve the quality of research reporting. Furthermore, journals have published editorial advice to advocate better reporting standards [6–9]. Nevertheless, it is arguable whether reporting standards have improved substantially [10–12].

In response to the poor quality of statistical reporting and data presentation in physiology and pharmacology, the Journal of Physiology published an editorial series to provide authors with clear, non-technical guidance on best-practice standards for data analysis, data presentation and reporting of results. Co-authored by the Journal of Physiology’s senior statistics editor and a medical statistician, the editorial series by Drummond and Vowler was jointly published in 2011 under a non-exclusive licence in the Journal of Physiology, the British Journal of Pharmacology, as well as several other journals. (The editorial series was simultaneously published, completely or in part, in Experimental Physiology, Advances in Physiology Education, Microcirculation, the British Journal of Nutrition, and Clinical and Experimental Pharmacology and Physiology.) The key recommendations by Drummond and Vowler include instructions to (1) report variability of continuous outcomes using standard deviations instead of standard errors of the mean, (2) report exact p-values for primary analyses and post-hoc tests, and (3) plot raw data used to calculate variability [13–15]. These recommendations were made so authors would implement them in future research reports. However, it is not known whether reporting practices in these journals have improved since the publication of this editorial advice.

We conducted a cross-sectional analysis of research papers published in the Journal of Physiology and the British Journal of Pharmacology to assess reporting practices. Specifically, we assessed statistical reporting, data presentation and spin in a random sample of papers published in the four years before and four years after the editorial advice by Drummond and Vowler was published.

## Materials and methods

### PubMed search and eligibility criteria

All papers published in the Journal of Physiology and the British Journal of Pharmacology in the years 2007-2010 and 2012-2015 and indexed on PubMed were extracted using the search strategy: (J Physiol[TA] OR Br J Pharmacol[TA]) AND yyyy: yyyy[DP] NOT (editorial OR review OR erratum OR comment OR rebuttal OR crosstalk). Papers were excluded if they were editorials, reviews, erratums, comments, rebuttals, or part of the Journal of Physiology’s Crosstalk correspondence series. Of the eligible papers, a random sample of papers published in the four years before the 2011 editorial advice by Drummond and Vowler was published (2007-2010) and four years after (2012-2015) was extracted (S1 File), and full-text PDFs were obtained.

### Question development and pilot testing

Ten questions and scoring criteria were developed to assess statistical reporting, data presentation and spin in the text and figures of the extracted papers. Questions assessing statistical reporting in the text (Q1-5) determined if and how written measures that summarize variability were defined, and if exact p-values were reported for primary analyses and post-hoc tests. Questions assessing data presentation in figures (Q6-8) determined if and how plotted measures that summarize variability were defined, and if raw data used to calculate the variability were plotted. Questions assessing the presence of spin (Q9-10) determined if p-values between 0.05 and 0.1 were interpreted as trends or statistically significant.

**Fig 1:**
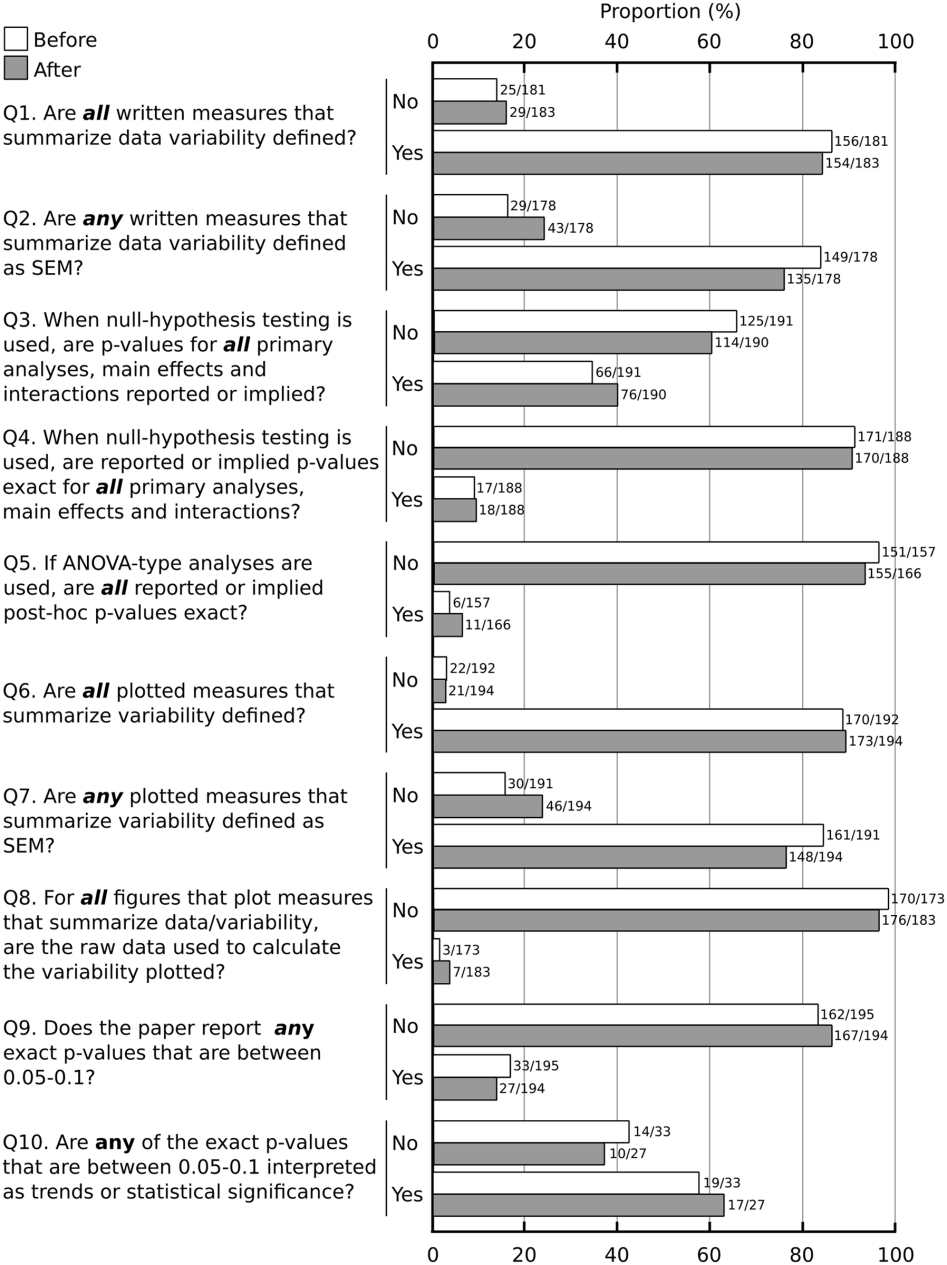
Descriptive results of statistical reporting, data presentation and spin. Counts and proportions of papers that fulfilled scoring criteria for each question before (white) and after (gray) the editorial advice was published. Abbreviations are SEM: standard error of the mean, ANOVA: analysis of variance.

A random sample of 20 papers before and 20 papers after the editorial advice was used to assess the clarity of the scoring instructions and scoring agreement between raters. These papers were separate from those included in the full audit. All papers were independently scored by three raters (AAB, JD, MEH). Scores that differed between raters were discussed to reach agreement by consensus. The wording of the questions, scoring criteria, and scoring instructions were refined to avoid different interpretations by raters. The questions are shown in Fig 1. Scoring criteria and additional details of the questions are provided in the scoring information sheets in the supporting information (S2 File).

### Data collection, outcomes and analysis

Each rater (AAB, JD, MEH, SCG) had to score and extract data independently from 50 papers before and 50 papers after the editorial advice was published. For each rater, papers from the 2007-2010 and 2012-2015 periods were audited in an alternating pattern to avoid an order or block effect. One rater unintentionally audited an additional 2007-2010 paper, and another rater unintentionally audited a 2007-2010 paper instead of a 2012-2015 paper. Thus, data from a random sample of 202 papers before and 199 papers after the editorial advice were analysed. When scoring was completed, papers that were difficult or ambiguous to score (less than 5% of all papers) were reviewed by all raters and scoring determined by consensus.

It was difficult to score some papers unambiguously on some of the scoring criteria. For example in question 3, it was sometimes difficult to determine what a paper’s primary analyses, main effects and interactions were, in order to determine whether p-values for these were reported or implied. When raters could not unambiguously interpret the data, either individually or as a team, we scored papers to give authors the benefit of doubt.

Counts and proportions of papers that fulfilled the scoring criteria for each question were calculated; no statistical tests were performed. Descriptive data are reported. All data processing and analysis were performed using Python (v3.5). Raw data, computer analysis code and result are available in the supporting information (S3 File).

## Results

The random sample of audited papers was reasonably representative of the number of papers published each year in the Journal of Physiology and the British Journal of Pharmacology in the two periods of interest (Table 1).

**Table 1:** Number of published and audited papers from the Journal of Physiology (JP) and the British Journal of Pharmacology (BJP).

Year	Published papers n* (JP, BJP)	Audited papers n (JP, BJP)
2007	1045 (648, 397)	66 (41, 25)
2008	824 (435, 389)	48 (23, 25)
2009	822 (418, 404)	55 (27, 28)
2010	740 (337, 403)	33 (12, 21)
**Total**	**3431 (1838, 1593)**	**202 (103, 99)**
2012	772 (390, 382)	62 (37, 25)
2013	762 (416, 346)	47 (23, 24)
2014	669 (321, 348)	47 (24, 23)
2015	771 (343, 368)	43 (13, 30)
**Total**	**2914 (1470, 1444)**	**199 (97, 102)**

* From PubMed: (J Physiol[TA] OR Br J Pharmacol[TA]) AND <year>[DP] NOT (editorial OR review OR erratum OR comment OR rebuttal OR crosstalk)

The proportions of audited papers that fulfilled the scoring criteria are presented in Fig 1. The figure shows there is no substantial difference in statistical reporting, data presentation or the presence of spin after the editorial advice was published. Overall, 76-84% of papers with written measures that summarized data variability used standard errors of the mean, and 90-96% of papers did not report exact p-values for primary analyses and post-hoc tests. 76-84% of papers that plotted measures to summarize variability used standard errors of the mean, and only 2-4% of papers plotted raw data used to calculate variability.

Of papers that reported p-values between 0.05 and 0.1, 56-63% interpreted such p-values as trends or statistically significant. Examples of such interpretations include:

- “A P < 0.05 level of significance was used for all analyses. […] As a result, further increases in the tidal P_oes_/VT (by 2.64 and 1.41 cmH_2_O l^−1^, P = 0.041) and effort–displacement ratios (by 0.22 and 0.13 units, P = 0.060) were consistently greater during exercise …” (PMID 18687714)
- “The level of IL-15 mRNA tended to be lower in vastus lateralis than in triceps (P = 0.07) (Fig 1A)” (PMID 17690139)
- “… where P < 0.05 indicates statistical significance […] was found to be slightly smaller than that of basal cells (−181 ± 21 pA, n = 7, P27–P30) but the difference was not quite significant (P = 0.05)” (PMID 18174213)
- “… resting activity of A5 neurons was marginally but not significantly higher in toxin-treated rats (0.9 ± 0.2vs. 1.8 ± 0.5 Hz, P = 0.068)” (PMID 22526887)
- “… significantly smaller than with fura-6F alone (P = 0.009), and slightly smaller than with fura-6F and EGTA (P = 0.08).” (PMID 18832426)
- “The correlation becomes only marginally significant if the single experiment with the largest effect is removed (r = 0.41, P = 0.057, n = 22).” (PMID 17916607)

Implied or gross spin (i.e. spin other than interpreting p-values between 0.05 and 0.1 as trends or statistically significant) was noted incidentally in papers before (n = 10) and after (n = 9) the editorial advice was published. Examples of statements where implied or gross spin was present include:

- “However, analysis of large spontaneous events (>50 pA in four of six cells) (Fig 1G–1I) showed the frequency to be increased from 0.4 ± 0.1 Hz to 0.8 ± 0.2 Hz (P < 0.05) (Fig 1G and 1H) and the amplitude by 20.3 ± 15.6 pA (P > 0.1) …” (PMID 24081159)
- “… whereas there was only a non-significant trend in the older group.” (no p-value, PMID 18469848)

Post-hoc analyses revealed audit results were comparable between raters (S4 File). Additional post-hoc analyses revealed audit results were relatively consistent across years and journals (S5 File). A notable exception was the British Journal of Pharmacology and its lower rate of reporting p-values (3-27% lower; question 3) and exact p-values for main analyses (8-22% lower; question 4).

## Discussion

In 2011 the Journal of Physiology and the British Journal of Pharmacology jointly published editorial advice on best practice standards for statistical reporting and data presentation [13]. These recommendations were reiterated in the Journals’ Instructions to Authors. Our cross-sectional analysis shows there was no substantial improvement in statistical reporting and data presentation in the four years after publication of this editorial advice.

Our results confirm that the quality of statistical reporting is generally poor. We found that ∼80% of papers that plotted error bars used standard error of the mean. In line with this, a systematic review of 703 papers published in key physiology journals revealed 77% of papers plotted bar graphs with standard error of the mean [16]. Similarly, one of the authors (MH) audited all 2015 papers published in the Journal of Neurophysiology and found that in papers with error bars, 65% used standard error of the mean and ∼13% did not define their error bars. That audit also revealed ∼42% of papers did not report exact p-values and ∼57% of papers with p-values between 0.05 and 0.1 interpreted these p-values as trends or statistically significant [12]. Our current study found that ∼93% of papers included non-exact p-values and ∼60% of papers with p-values between 0.05 and 0.1 reported these with spin. It is unfortunate that authors adopt practices that distort the interpretation of results and mislead readers into viewing results more favorably. This problem was recently highlighted by a systematic review on the prevalence of spin in the biomedical literature [17]. Spin was present in 35% of randomized control trials with significant primary outcomes, 60% of randomized controls with non-significant primary outcomes, 84% of non-randomized trials and 86% of observational studies. Overall, these results highlight the sheer magnitude of the problem: poor statistical reporting and questionable interpretation of results are truly common practice for many scientists.

Our findings also broadly agree with other observational data on the ineffectiveness of statistical reporting guidelines in biomedical and clinical research. For example, the CONSORT guidelines for the reporting of randomized controlled trials are widely supported and mandated by key medical journals, but the quality of statistical reporting and data presentation in randomized trial reports remains inadequate [18–20]. A scoping audit of papers published by American Physiological Society journals in 1996 showed most papers mistakenly reported standard errors of the mean as estimates of variability, not as estimates of uncertainty [21]. Consequently, in 2004 the Society published editorial guidelines to improve statistical reporting practices [22]. These guidelines instructed authors to report variability using standard deviations, and report uncertainty about scientific importance using confidence intervals. However, the authors of the guidelines audited papers published before and after their implementation and found no improvement in the proportion of papers reporting standard errors of the mean, standard deviations, confidence intervals, and exact p-values [10]. Likewise, in 1999 and 2001 the American Psychological Association published guidelines instructing authors to report effect sizes and confidence intervals [23, 24]. Once again, an audit of papers published before and after the guidelines were implemented found no improvement in the proportion of figures with error bars defined as standard errors of the mean (43-59%) or worse, with error bars that were not defined (29-34%) [25].

One example where editorial instructions improved reporting practices occurred in public health. In the mid-80’s the American Journal of Public Health had an influential editor who advocated and enforced the use of confidence intervals rather than p-values. An audit of papers published before and during the tenure of this editor found that the reliance on p-values to interpret findings dropped from 63% to 5% and the reporting of confidence intervals increased from 10% to 54% [26]. However, few authors referred to confidence intervals when interpreting results. In psychology, when editors of Memory & Cognition and the Journal of Consulting and Clinical Psychology enforced the use of confidence intervals and effect sizes, the use of these statistics increased to some extent, even though long-term use was not maintained [27]. These examples provide evidence that editors with training in statistical intepretation may enforce editorial instructions more successfully, even if author understanding does not necessarily improve.

Why are reporting practices not improving? The pressure to publish may be partly to blame. Statistically significant findings that are visually and numerically clean are easier to publish. Thus, it should come as no surprise that p-values between 0.05 and 0.1 are interpreted as trends or statistically significant, and that researchers use standard errors of the mean to plot and report results. There is also a cultural component to these practices. The process of natural selection ensures that practices associated with higher publication rates are transmitted from one generation of successful researchers to the next [28]. Unfortunately, some of these practices include poor reporting practices. As was recently highlighted by Goodman [29], conventions die hard, even if they contribute to irreproducible research. In the article, citing a government report on creating change within a system, Goodman highlights that “culture will trump rules, standards and control strategies every single time”. Thus, researchers will often opt for reporting practices that make their papers look like others in their field, conscious or not that these reporting practices are inadequate and not in line with published reporting guidelines. A final contributing factor is that many researchers continue to misunderstand key statistical concepts, such as measures of variability and uncertainty, inferences made from independent and repeated-measures study designs, and error bars and how they reflect statistical significance [30]. This partly explains the resistance to statistical innovations and robust reporting practices [27].

The recent reproducibility crisis has seen all levels of the scientific community implement new strategies to improve how science is conducted and reported. For example, journals have introduced article series to promote awareness [9, 31] and adopted more stringent reporting guidelines [32, 33]. Whole disciplines have also taken steps to tackle these issues. For example, the Academy of Medical Sciences partnered with the Biotechnology and Biological Sciences Research Council, the Medical Research Council and the Wellcome Trust to host a symposium on improving reproducibility and reliability of biomedical research [34]. Funding bodies have also acted. For example, the NIH launched training modules to educate investigators on topics such as bias, blinding and experimental design [35], and the Wellcome Trust published guidelines on research integrity and good research practice [36]. Other initiatives include the Open Science Framework, which facilitates open collaboration [37], and the Transparency and Openness Promotion guidelines, which were developed to improve reproducibility in research and have been endorsed by many key journals [38]. To improve research practices, these initiatives aim to raise awareness of the issues, educate researchers and provide tools to implement the various recommendations. While the enduring success of these initiatives remains to be determined, we remain hopeful for the future. There is considerable momentum throughout science, and many leaders from various disciplines have stepped up to lead the way.

## Conclusion

In summary, reporting practices have not improved despite published editorial advice. Journals and other members of the scientific community continue to advocate and implement strategies for change, but these have only had limited success. Stronger incentives, better education and widespread enforcement are needed for enduring improvements in reporting practices to occur.

## Supporting information

S1 File Random paper selection. Python code and PubMed search results used to randomly select papers for the audit. See the included README.txt file for a full description.
(ZIP)

S2 File Scoring information sheets. Scoring criteria and details of questions 1-10.
(PDF)

S3 File Data and code. Comma-separated-values (CSV) file of raw scores for questions 1-10 and the Python files used to analyse the data. See the included README.txt file for a full description.
(ZIP)

S4 File Audit results across raters. Comparison of audit results across raters indicates the scoring criteria were applied uniformly across raters.
(PDF)

S5 File Audit results across years and journals. Comparison of audit results for each year and journal. The British Journal of Pharmacology consistently had lower reporting rates of p-values for main analyses, and exact p-values for main analyses.
(PDF)
