# Supplementary material for: Poor statistical reporting, inadequate data presentation and spin persist despite editorial advice
Source: PLoS One. 2018 Aug 15;13(8):e0202121. doi: 10.1371/journal.pone.0202121 (PMC6093658; doi:10.1371/journal.pone.0202121)
Supplement: S2 File — Scoring criteria and details of questions 1-10. (PDF) [file pone.0202121.s002.pdf]

If you have a situation that you consider falls in a grey zone/area, give the benefit of the doubt to the authors.

If you don't think it is possible to answer a question because something is not clear, mark that question with '???' to discuss at group review.

Q1	Are ***all*** written measures that summarize data variability defined?
Q2	Are ***any*** written measures that summarize data variability defined as SEM?
Q3	When null-hypothesis testing is used, are p-values for ***all*** primary analyses, main effects and interactions reported or implied?
Q4	When null-hypothesis testing is used, are reported or implied p-values exact for ***all*** primary analyses, main effects and interactions?
Q5	If ANOVA-type analyses are used, are ***all*** reported or implied post-hoc p-values exact?
Q6	Are ***all*** plotted measures that summarize variability defined?
Q7	Are ***any*** plotted measures that summarize variability defined as SEM?
Q8	For ***all*** figures that plot measures that summarize data/variability, are the raw data used to calculate the variability plotted?
Q9	Does the paper report ***any*** exact p-values that are between 0.05-0.1 ?
Q10	Are ***any*** of the exact p-values that are between 0.05-0.1 interpreted as trends or statistical significance?

Q1 Are ***all*** written measures that summarize data variability defined?

DEFINITIONS

Measures that summarize data variability.
These include the standard deviation (SD), the standard error of the mean (SEM), 95% CI, the interquartile or similar range (IQR) and the range.

SCOPE
Numerical values reported in the main text of the article, tables or figure legends.

When authors report that 'All measures/results' or 'Measures/results' are reported as mean±SEM, mean±SD, median [IQR], etc., consider this to apply to all written measures that summarize data variability.

POSSIBLE ANSWERS

NA
No such measures are reported.

0 or no
Such measures are reported, but the type of at least one cannot be determined.

1 or yes
Such measures are reported and the type of all measures can be determined.

0 (no)	1 (yes)
"Their mean age was 27.3 (4.5)."	"Their mean age was 27.3 (4.5) (mean (SD))."
"The cats weighed 1.6±0.4 kg (mean ±SEM), and the amount of food they ate varied considerably (100 g/day [50-375])."	"The cats weighed 1.6±0.4 kg (mean ±SEM), and the amount of food they ate varied considerably (100 g/day [50-375]; median [range])."
"Their mean age was 32±4. Stimulus intensity was adjusted for each subjects (1.3±0.4mA) before the start of the next trial. [...] The effect of brain stimulation on hot dog cravings is reported as mean ± SEM."	"Their mean age was 32±4. Stimulus intensity was adjusted for each subjects (1.3±0.4mA) before the start of the next trial. [...] Results are reported as mean ± SEM."
"**Figure 3. Response rate.** There was a marked increase in the response rate of blue turtles (a). This may reflect their slow cervical reflex (23.4±4.8 ms)."	"**Figure 3. Response rate.** There was a marked increase in the response rate of blue turtles (a). This may reflect their slow cervical reflex (23.4±4.8 ms; mean±SEM)."

0 (no):

TABLE 1.

GROUP	IQ SCORES
treatment	98 (5)
control	106 (7)
placebo	94 (4)

1 (yes):

TABLE 1.

GROUP	IQ SCORES*
treatment	98 (5)
control	106 (7)
placebo	94 (4)

* values are mean (SD)

Q2 Are ***any*** written measures that summarize data variability defined as SEM?

DEFINITIONS
Measures that summarize data variability.
These include the standard deviation (SD), the standard error of the mean (SEM), 95% CI, the interquartile or similar range (IQR) and the range.

SCOPE
Numerical values reported in the main text of the article, tables or figure legends.

POSSIBLE ANSWERS
NA
No such measures are reported, or measures are not defined.

0 or no
No.

1 or yes
Yes.

0 (no)	1 (yes)
"Their mean age was 27.3 (4.5)."	"Their mean age was 27.3 (4.5), mean (SEM)."
"The cats weighed 1.6±0.4 kg (mean ±SD), and the amount of food they ate varied considerably (100 g/day [50-375]; median [range])."	"The cats weighed 1.6±0.4 kg (mean ±SEM), and the amount of food they ate varied considerably (100 g/day [50-375]; median [range])."
"Their mean age was 32±4. Stimulus intensity was adjusted for each subjects (1.3±0.4mA) before the start of the next trial. [...] The effect of brain stimulation on hot dog cravings is reported as mean ± SD."	"Their mean age was 32±4. Stimulus intensity was adjusted for each subjects (1.3±0.4mA) before the start of the next trial. [...] Results are reported as mean ± SEM."

Q3 When null-hypothesis testing is used, are p-values for ***all*** primary analyses, main effects and interactions reported or implied?

DEFINITIONS

Main effects and interactions. Results of ANOVA-type statistical tests.

Primary analyses. Statistical tests that are ***not*** post-hoc tests of an ANOVA-type analysis. Examples include t-tests, Chi-square tests, Pearson's product-momment correlations, regression-type analyses. If several t-tests are performed when an ANOVA would be more appropriate, these t-tests will be considered as primary analyses.

Implied. For example "There was no main effect of *group*" or "The was no age difference between groups" or "The *group* by *time* interaction was non-significant". Simply stating ANOVA before reporting post-hoc results does **NOT** count.

SCOPE

Reported or implied p-values may be located in the main text of the paper, tables or figures.

POSSIBLE ANSWERS

NA

The paper does not include primary analyses, main effects or interactions.

0 or no

The paper includes primary analyses, main effects or interactions, but at least one of these does not have a reported or implied p-value.

1 or yes

The paper includes primary analyses, main effects or interactions, and all of these have reported or implied p-values.

Q3 When null-hypothesis testing is used, are p-values for ***all*** primary analyses, main effects and interactions reported or implied?

0 (no)

“The cells were inhibited by the DOWN-receptor at time-point 2, 3 and 6 (Dunnett post-hoc test, p<0.05).”

“The cells were inhibited by the DOWN-receptor at time-point 2, 3 and 6 (ANOVA, Dunnett post-hoc test, p<0.05).”

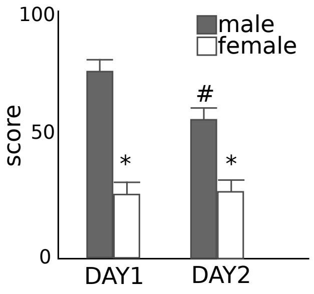

* Different from male on same day, p<0.01
# Male different between days, p<0.05

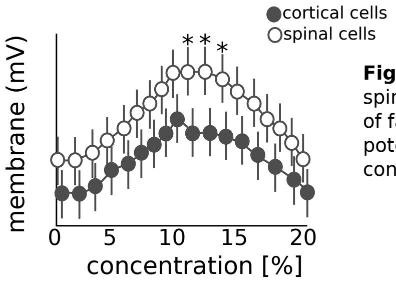

Figure 3. Plotted are the results for cortical and spinal cells when exposted to different concentration of factor #452-Zion. As can be seen, the membrane potential of spinal cells was significantly less when the concentration was 11, 12 and 13 % (* p<0.05).

1 (yes)

“The DOWN-receptor had a ***significant*** impact on cell excitability, causing significant inhibited at time-points 2, 3 and 6 (Dunnett post-hoc test, p<0.05).” (**implied**)

“The DOWN-receptor had a significant impact on cell excitability ***(F(42,2)=22.4, p=0.012)***, causing significant inhibited at time-points 2, 3 and 6 (Dunnett post-hoc test, p<0.05).” (**reported**)

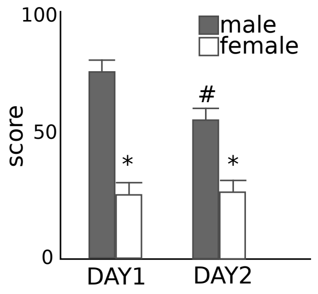

main effect of gender: $F_{(66,1)}$ = 42.5, p < 0.001
main effect of day: $F_{(2,1)}$ = 24.8, p = 0.024
gender by day interaction: $F_{(30,4)}$ = 19.0, p = 0.016

* Different from male on same day, p<0.01
# Male different between days, p<0.05

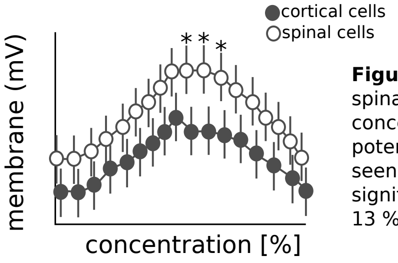

Figure 3. Plotted are the results for cortical and spinal cells showing the significant effect of different concentration of factor #452-Zion on membrane potential excitability ($F_{(128,21)}$=54.2, p=0.003). As can be seen, the membrane potential of spinal cells was significantly less when the concentration was 11, 12 and 13 % (Bonferonni post-hoc * p<0.05).

Q4 When null-hypothesis testing is used, are reported or implied p-values exact for ***all*** primary analyses, main effects and interactions?

DEFINITIONS

Main effects and interactions. Results of ANOVA-type statistical tests.

Primary analyses. Statistical tests that are ***not*** post-hoc tests of an ANOVA-type analysis. Examples include t-tests, Chi-square tests, Pearson's product-momment correlations, regression-type analyses. If several t-tests are performed when an ANOVA would be more appropriate, these t-tests will be considered as primary analyses.

Implied. For example "There was no main effect of *group*" or "The was no age difference between groups" or "The *group* by *time* interaction was non-significant".

exact. Refers to p = 0.xxx (e.g., p=0.012, p=0.564, p=0.002) and p<0.001.

SCOPE

Reported or implied p-values may be located in the main text of the paper, tables or figures.

POSSIBLE ANSWERS

NA
The paper does not report primary analyses, main effects or interactions.

0 or no
No.

1 or yes
Yes.

Q4 When null-hypothesis testing is used, are reported or implied p-values exact for ***all*** primary analyses, main effects and interactions?

0 (no)

"The DOWN-receptor had a significant impact on cell excitability, causing significant inhibition at time-points 2, 3 and 6 (Dunnett post-hoc test, p<0.05)."

"The DOWN-receptor had a significant impact on cell excitability (p<0.05), causing significant inhibited at time-points 2, 3 and 6 (Dunnett post-hoc test, p<0.05)."

"The ANOVA revealed a main effect of time (P<0.05) and a main effect of group (p<0.01), but no time by group interaction (p>0.05)."

"The ANOVA revealed a main effect of time (P=0.012), but there was no main effect of group or time by group interaction."

"The subjects weight was significantly correlated with age (r=0.12), income (r=0.09) and foot length (r=0.56). The other correlation were not significant."

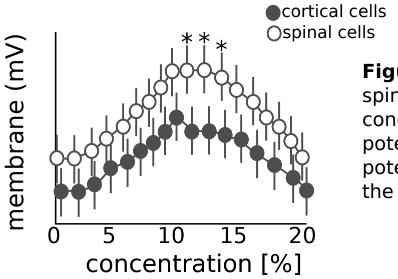

Figure 3. Plotted are the results for cortical and spinal cells showing the significant effect of different concentration of factor #452-Zion on membrane potential excitability. As can be seen, the membrane potential of spinal cells was significantly less when the concentration was 11, 12 and 13 % (* p<0.05).

1 (yes)

"The DOWN-receptor had a significant impact on cell excitability (F(42,2) =22.4, p=0.012), causing significant inhibition at time-points 2, 3 and 6 (Dunnett post-hoc test, p<0.05)."

"The DOWN-receptor had a significant impact on cell excitability (F(42,2) =22.4, p=0.012), causing significant inhibited at time-points 2, 3 and 6 (Dunnett post-hoc test, p<0.05)."

"The ANOVA revealed a main effect of time (P=0.012) and a main effect of group (p<0.001), but no time by group interaction (p=0.545)."

"The ANOVA revealed a main effect of time (P=0.012) and a main effect of group (p<0.001), but no time by group interaction (p=0.545)."

"The subjects weight was significantly correlated with age (r=0.12, p=0.012), income (r=0.09, p=0.0258) and foot length (r=0.56, p<0.001). However, weight was not correlated with IQ (r=0.16, p=0.556) or perceived index finger length (r=0.01, p=0.06)"

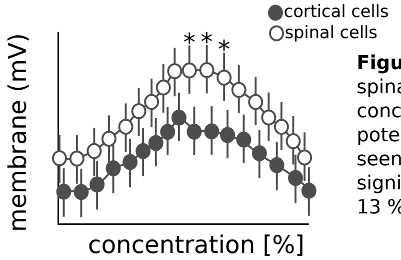

Figure 3. Plotted are the results for cortical and spinal cells showing the significant effect of different concentration of factor #452-Zion on membrane potential excitability ($F_{(128,21)}$=54.2, p=0.003). As can be seen, the membrane potential of spinal cells was significantly less when the concentration was 11, 12 and 13 % (Bonferonni post-hoc * p<0.05).

Q5 If ANOVA-type analyses are used, are ***all*** reported or implied post-hoc p-values exact?

DEFINITIONS

Implied. For example "Values at 10 and 15 min were significantly different from baseline".

exact. Refers to p = 0.xxx (e.g., p=0.012, p=0.564, p=0.002) and p<0.001.

SCOPE
Reported or implied post-hoc p-values may be located in the main text of the paper, tables or figures.

POSSIBLE ANSWERS

NA
The paper does not report or imply any post-hoc comparisons.

0 or no
No.

1 or yes
Yes.

**0 (no)**	**1 (yes)**
"The DOWN-receptor caused a significant inhibition at time-points 2, 3 and 6 (p<0.05)."	"The DOWN-receptor caused a significant inhibition at time-points 2, 3 and 6 (p<0.001)." "The DOWN-receptor caused a significant inhibition at time-points 2 (p=0.021), 3 (p=0.004) and 6 (p = 0.019)."
"Post-hoc comparison revealed a significant reduction in membrane potential at the two highest stimulus intensities."	"Post-hoc comparison revealed a significant reduction in membrane potential at 2mA (p=0.038) and 5mA (p<0.001)."

0 (no)

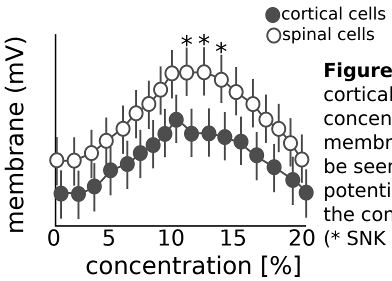

Figure 3. Plotted are the results for cortical and spinal cells for different concentration of factor #452-Zion on membrane potential excitability. As can be seen, spinal cell membrane potentials were significantly less when the concentration was 11, 12 and 13% (* SNK post-hoc, p<0.05).

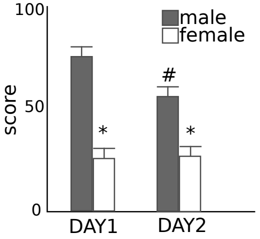

* Different from males on same day, p<0.01
# Male different between days, p<0.05

1 (yes)

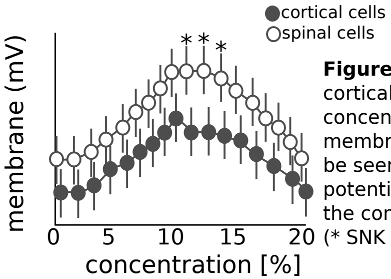

Figure 3. Plotted are the results for cortical and spinal cells for different concentration of factor #452-Zion on membrane potential excitability. As can be seen, spinal cell membrane potentials were significantly less when the concentration was 11, 12 and 13% (* SNK post-hoc, p<0.001).

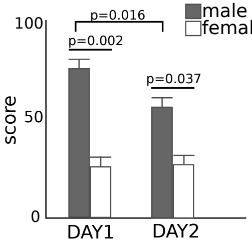

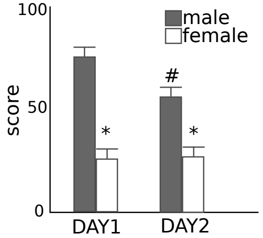

* Different from males on same day, p<0.001
# Male different between days, p=0.042

Q6 Are *all* plotted measures that summarize variability defined?

DEFINITIONS

Measures that summarize data variability. These include the standard deviation (SD), the standard error of the mean (SEM), 95% CI, the interquartile or similar range (IQR) and the range.

SCOPE

Figures that are included are those that visually summarize data variability, whether it be for multiple samples from a single subject, or data from multiple subjects.

POSSIBLE ANSWERS

NA
The paper does not contain such figures.

0 or no
No.

1 or yes
Yes.

0 (no)

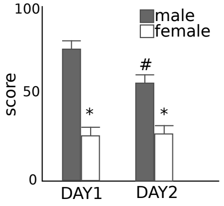

Figure 1. Effect of factor Z23#15 on scores of male and female subjects. * Different from males on same day, p<0.01. # Male different between days, p<0.05

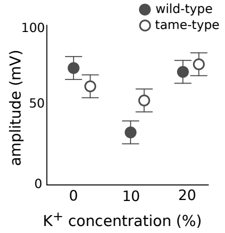

Figure 1. Effect of K+ concentration on retinal reflex amplitude for wild- and tame-type mice.

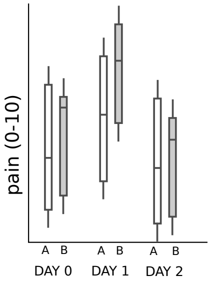

Figure 1. Change in pain scores across days for people who had to only use words starting with 'A' or 'B'.

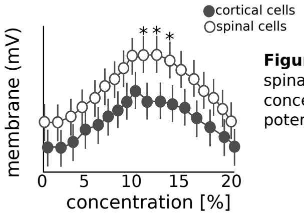

Figure 3. Plotted are the results for cortical and spinal cells showing the significant effect of different concentration of factor #452-Zion on membrane potential excitability. (* p<0.05).

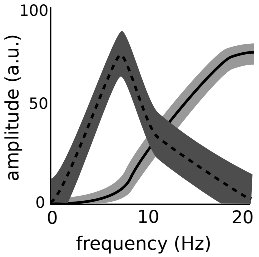

Figure 2. Individual subject data showing the frequency response of the toe (dashed line) and hip joint (solid line).

1 (yes)

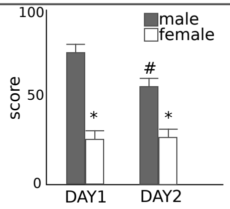

Statistical analysis
Blah Blah [...]. Results are reported as mean±SD.

OR

Figure 1. Effect of factor Z23#15 on scores of male and female subjects. * Different from males on same day, p<0.01. # Male different between days, p<0.05. Values are mean±SD.

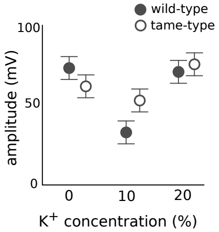

Statistical analysis
Blah Blah [...]. All values are reported as mean±SEM.

OR

Figure 1. Effect of K+ concentration on retinal reflex amplitude for wild- and tame-type mice. Values are mean±SEM.

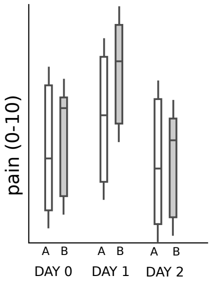

Statistical analysis
Blah Blah [...]. All values are reported as median and interquartile range. In figures, vertical lines depict range.

OR

Figure 1. Change in pain scores across days for people who had to only use words starting with 'A' or 'B'. Boxplots represent median and interquartile range; vertical lines depict range.

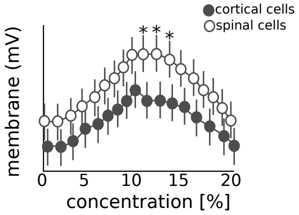

Statistical analysis
Blah Blah [...]. All values are reported as mean±SD.

OR

Figure 3. Plotted are the results for cortical and spinal cells showing the significant effect of different concentration of factor #452-Zion on membrane potential excitability. (* p<0.05). Values are mean±SD.

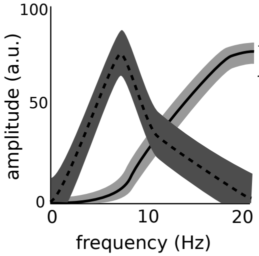

Figure 2. Individual subject data showing the frequency response of the toe (dashed line) and hip joint (solid line). Shadded area represent 95% confidence interval.

Q7 Are ***any*** plotted measures that summarize variability defined as SEM?

DEFINITIONS

Measures that summarize data variability. These include the standard deviation (SD), the standard error of the mean (SEM), 95% CI, the interquartile or similar range (IQR) and the range.

SCOPE

Figures that are included are those that visually summarize **data variabilty**, whether it be for multiple samples from a single subject, or data from multiple subjects. **Do not** consider figures without measures that summarize variability.

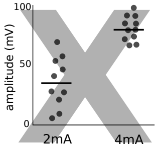

POSSIBLE ANSWERS

NA

The paper does not contain such figures.

0 or no

No.

1 or yes

0 (no)

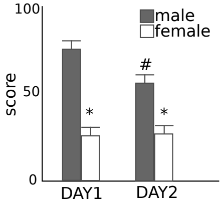

Figure 1. Effect of factor Z23#15 on scores of male and female subjects. * Different from males on same day, p<0.01. # Male different between days, p<0.05. Error bars represent 95% CI.

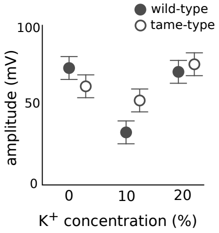

Figure 1. Effect of K+ concentration on retinal reflex amplitude for wild- and tame-type mice. Values represent mean±SD.

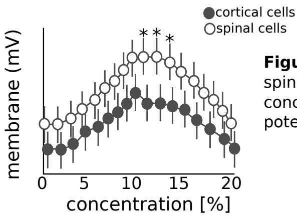

Figure 3. Plotted are the results for cortical and spinal cells showing the significant effect of different concentration of factor #452-Zion on membrane potential excitability. (* p<0.05).

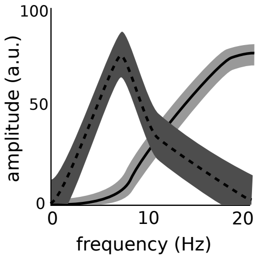

Figure 2. Individual subject data showing the frequency response of the toe (dashed line) and hip joint (solid line). Shadded area represents 95% CI.

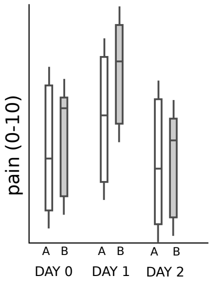

Figure 1. Change in pain scores across days for people who had to only use words starting with 'A' or 'B'. Boxplots represent median and interquartile range; vertical lines depict range.

1 (yes)

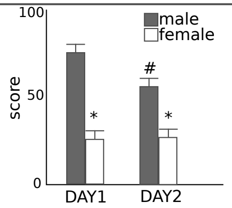

Statistical analysis
Results are reported as mean±SEM.

OR

Figure 1. Effect of factor Z23#15 on scores of male and female subjects. * Different from males on same day, p<0.01. # Male different between days, p<0.05. Values are mean±SEM.

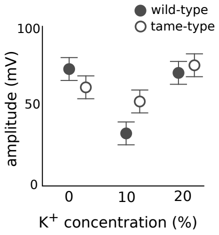

Statistical analysis
All values are reported as mean±SEM.

OR

Figure 1. Effect of K+ concentration on retinal reflex amplitude for wild- and tame-type mice. Values are mean±SEM.

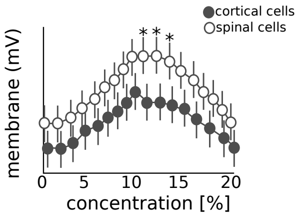

Statistical analysis
All values are reported as mean±SD.

OR

Figure 3. Plotted are the results for cortical and spinal cells showing the significant effect of different concentration of factor #452-Zion on membrane potential excitability. (* p<0.05). Values are mean±SEM.

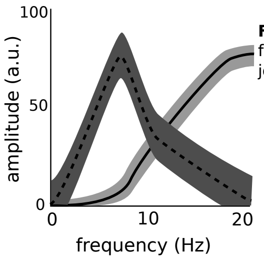

Figure 2. Individual subject data showing the frequency response of the toe (dashed line) and hip joint (solid line). Shadded area represent SEM.

Q8 For ***all*** figures that plot measures that summarize data/variability, are the raw data used to calculate the variability plotted?

DEFINITIONS

Measures that summarize data variability. These include the standard deviation (SD), the standard error of the mean (SEM), 95% CI, the interquartile or similar range (IQR) and the range.

SCOPE

Figures that that are included are those that visually summarize data variabilty, whether it be for multiple samples from a single subject, or data from multiple subjects.

Figures that connect sequential values with a line ***are not*** to be considered.

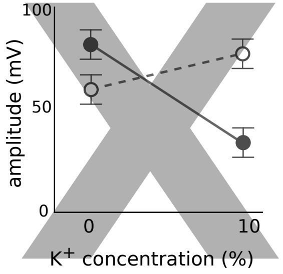

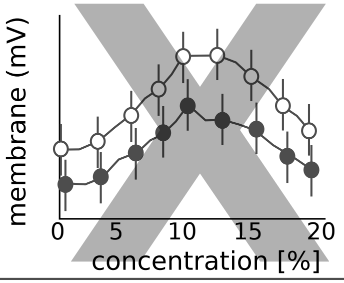

POSSIBLE ANSWERS

NA

The paper does contain such figures.

0 or no

No.

1 or yes

Yes.

**0 (no)**	**1 (yes)**
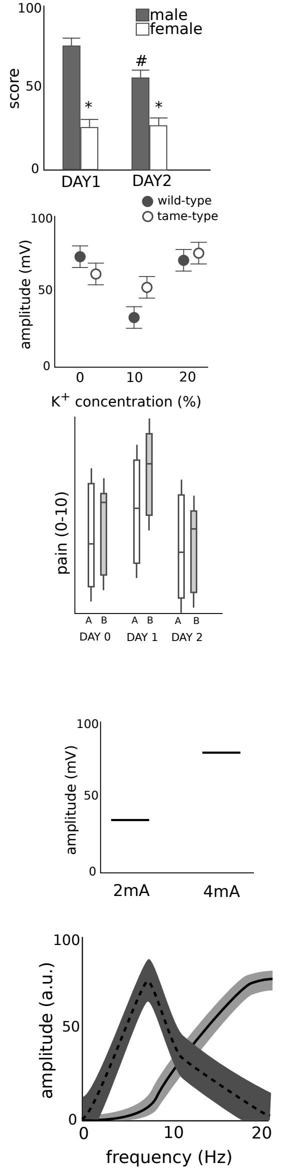	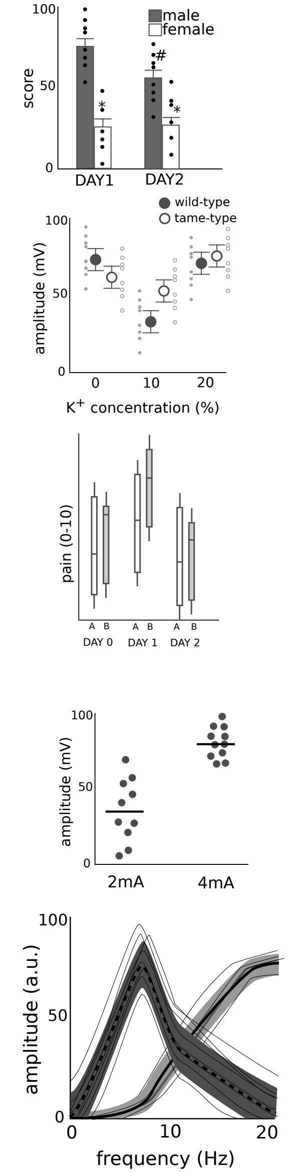

Q9 Does the paper report ***any*** exact p-values that are between 0.05-0.1 ?

SCOPE
This relates to exact p-values (p=0.1; p=0.0742) reported in the main text of the article, tables and figures.

POSSIBLE ANSWERS
NA
The paper does not report p-values.
0 or no
No.
1 or yes
Yes.

Q10 Are ***any*** of the exact p-values that are between 0.05-0.1 interpreted as trends or statistically significant?

SCOPE
This relates to exact p-values (p=0.0742) reported in the main text of the article, tables and figures. **!!** If there is spin, consider copying the relevant text in the Comment section.
POSSIBLE ANSWERS
NA
The paper does not report exact p-values between 0.05-0.1.
0 or no
No.
1 or yes
Yes.

0 (no)	1 (yes)
"The effect of heat was not significant (p=0.062)."	"There was a trend for heat to effect the outcome (p=0.062)."<br><br>"The effect of heat was significant (p=0.062)."
