# Supplementary figures and images for: Poor statistical reporting, inadequate data presentation and spin persist despite editorial advice
Source: PLoS One. 2018 Aug 15;13(8):e0202121. doi: 10.1371/journal.pone.0202121 (PMC6093658; doi:10.1371/journal.pone.0202121)

Question 10: Are any p-values between 0.05-0.1 interpreted as trends/significance?

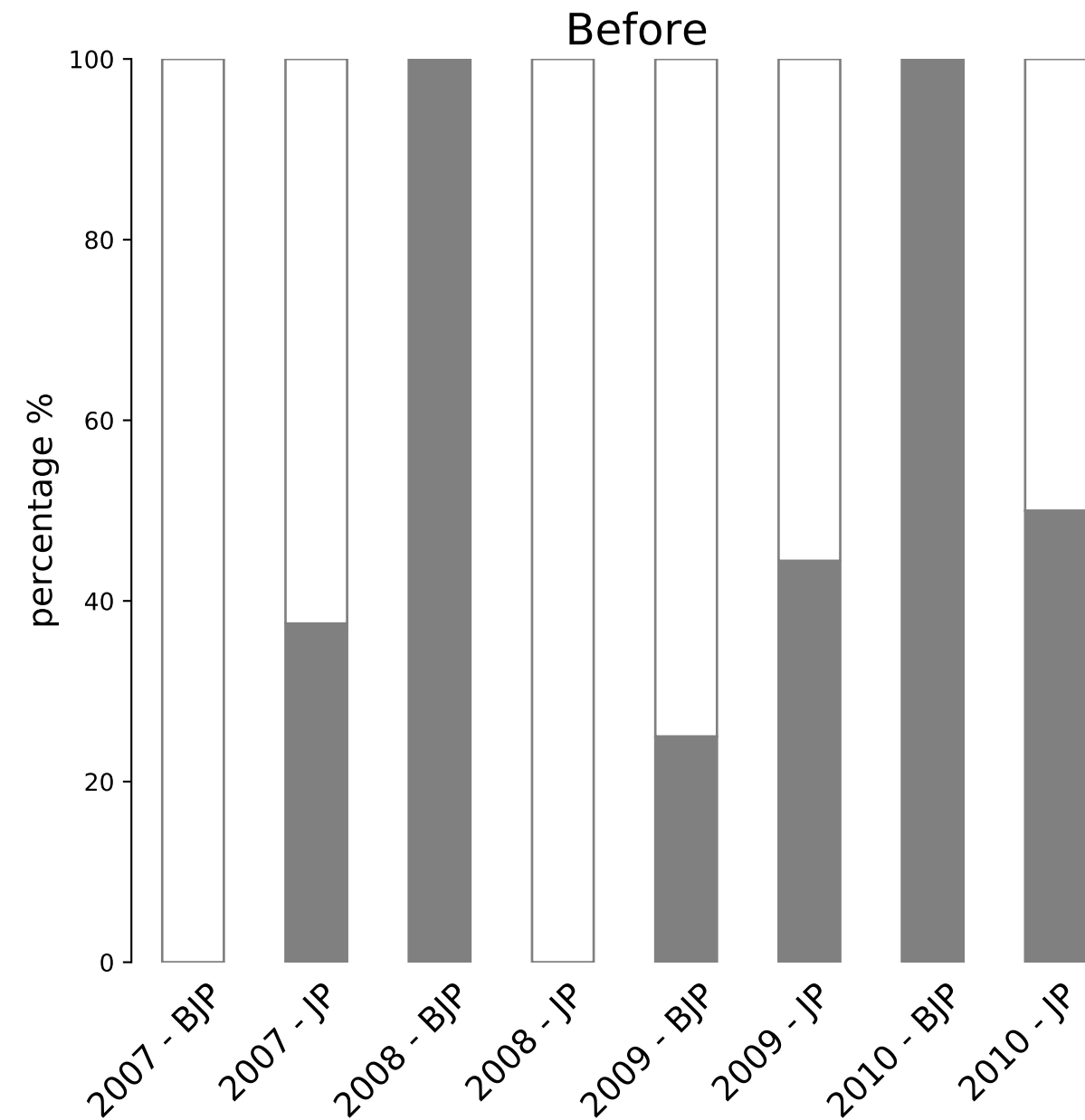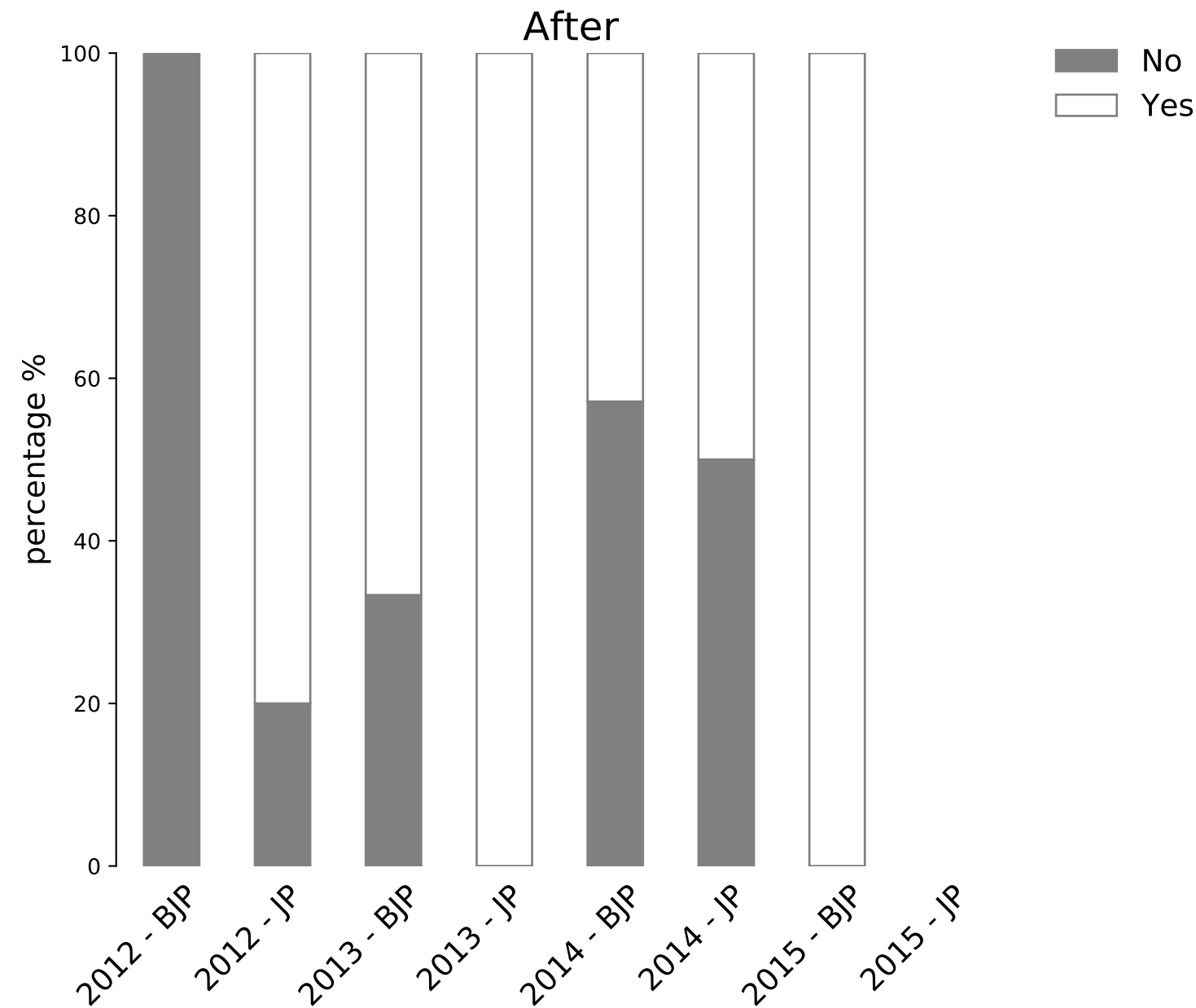

Supplement: S3 File — Comma-separated-values (CSV) file of raw scores for questions 1-10 and the Python files used to analyse the data. See the included README.txt file for a full description. (ZIP) [file pone.0202121.s003.zip › S3 File/supplementary_results/suppl_q10_year_journal.pdf]

# Question 9: Does the paper report any exact p-values that are between 0.05-0.1?

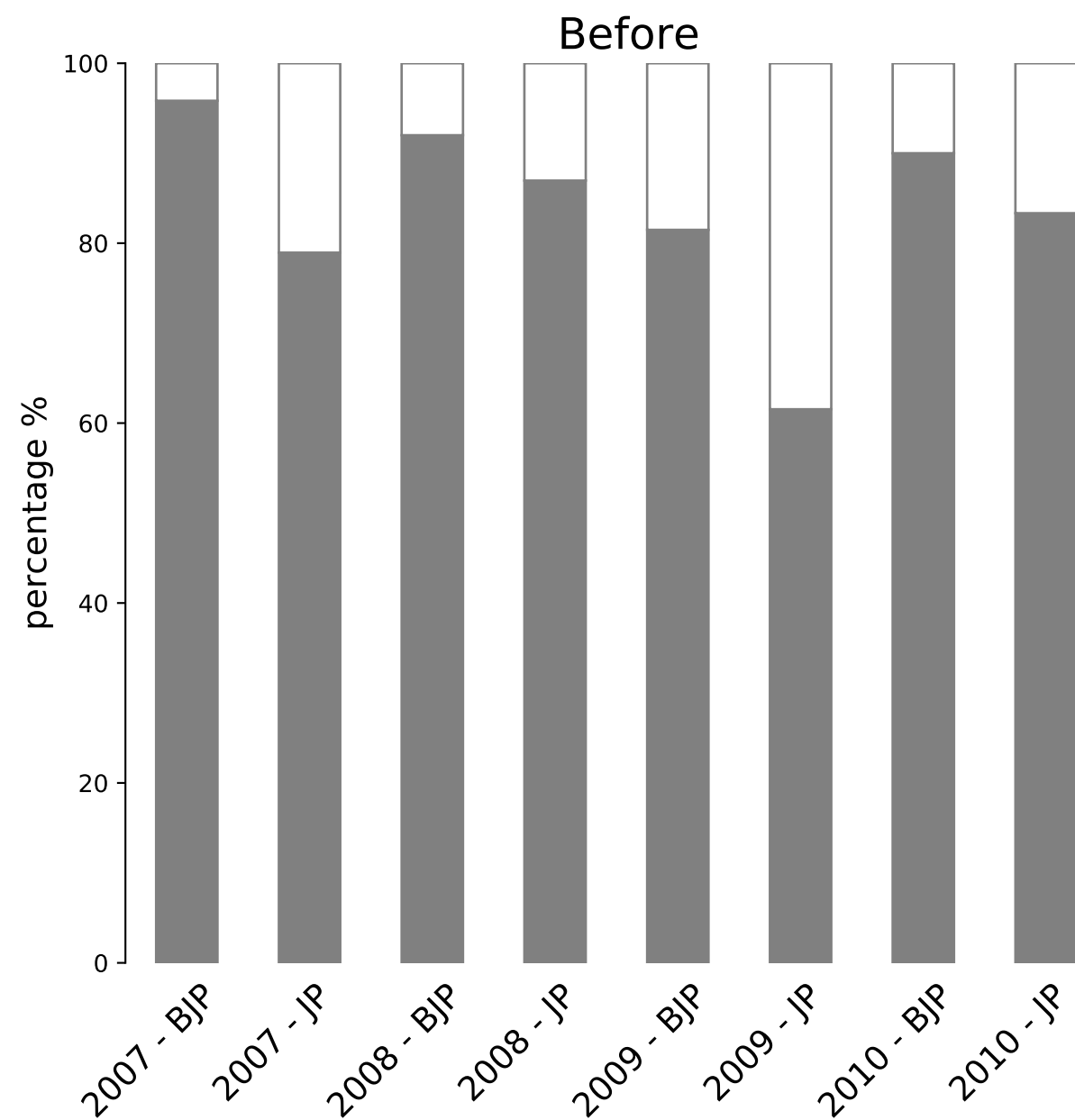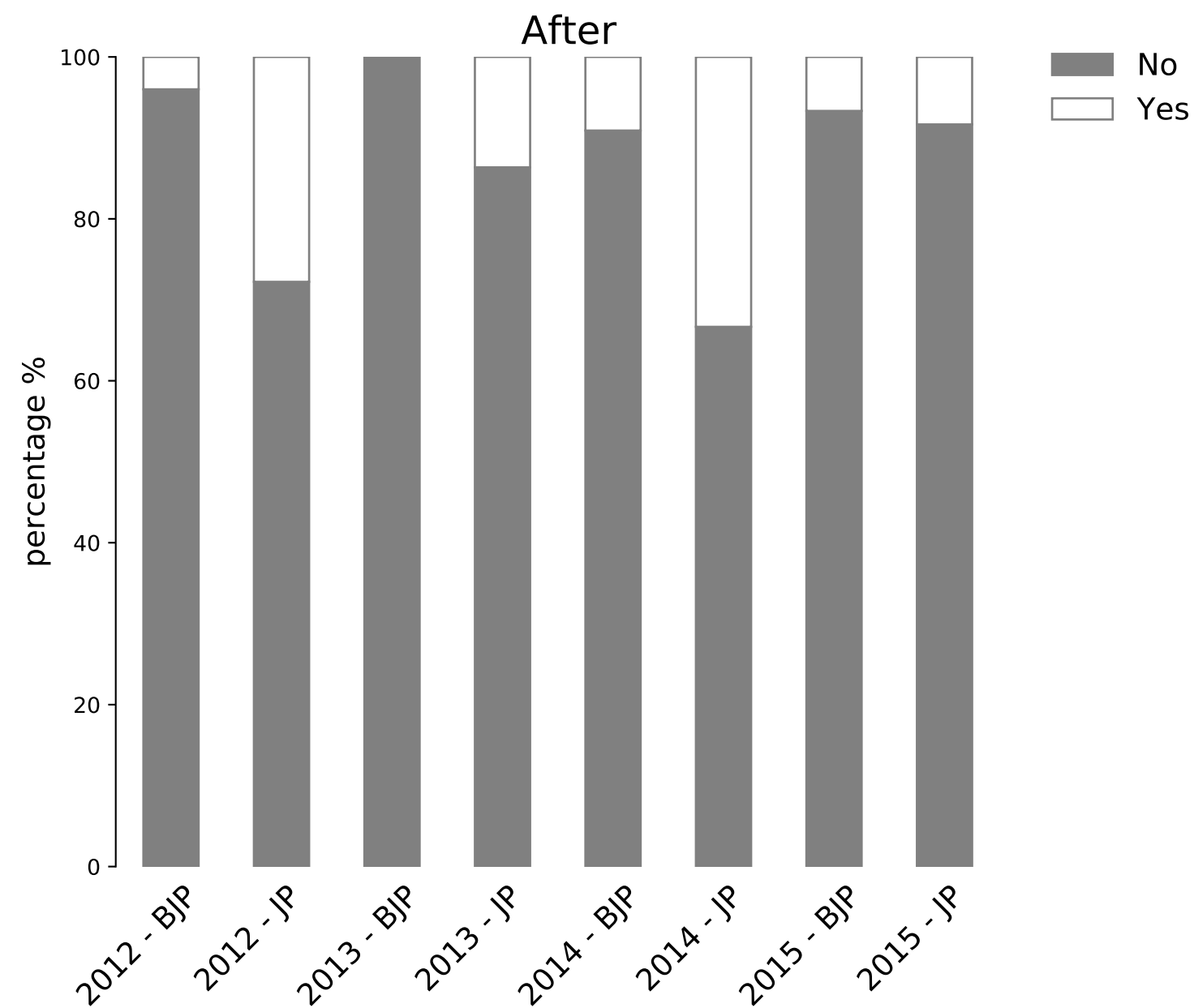

Supplement: S3 File — Comma-separated-values (CSV) file of raw scores for questions 1-10 and the Python files used to analyse the data. See the included README.txt file for a full description. (ZIP) [file pone.0202121.s003.zip › S3 File/supplementary_results/suppl_q9_year_journal.pdf]

Question 8: For all figures that summarize data/variability, are raw data plotted?

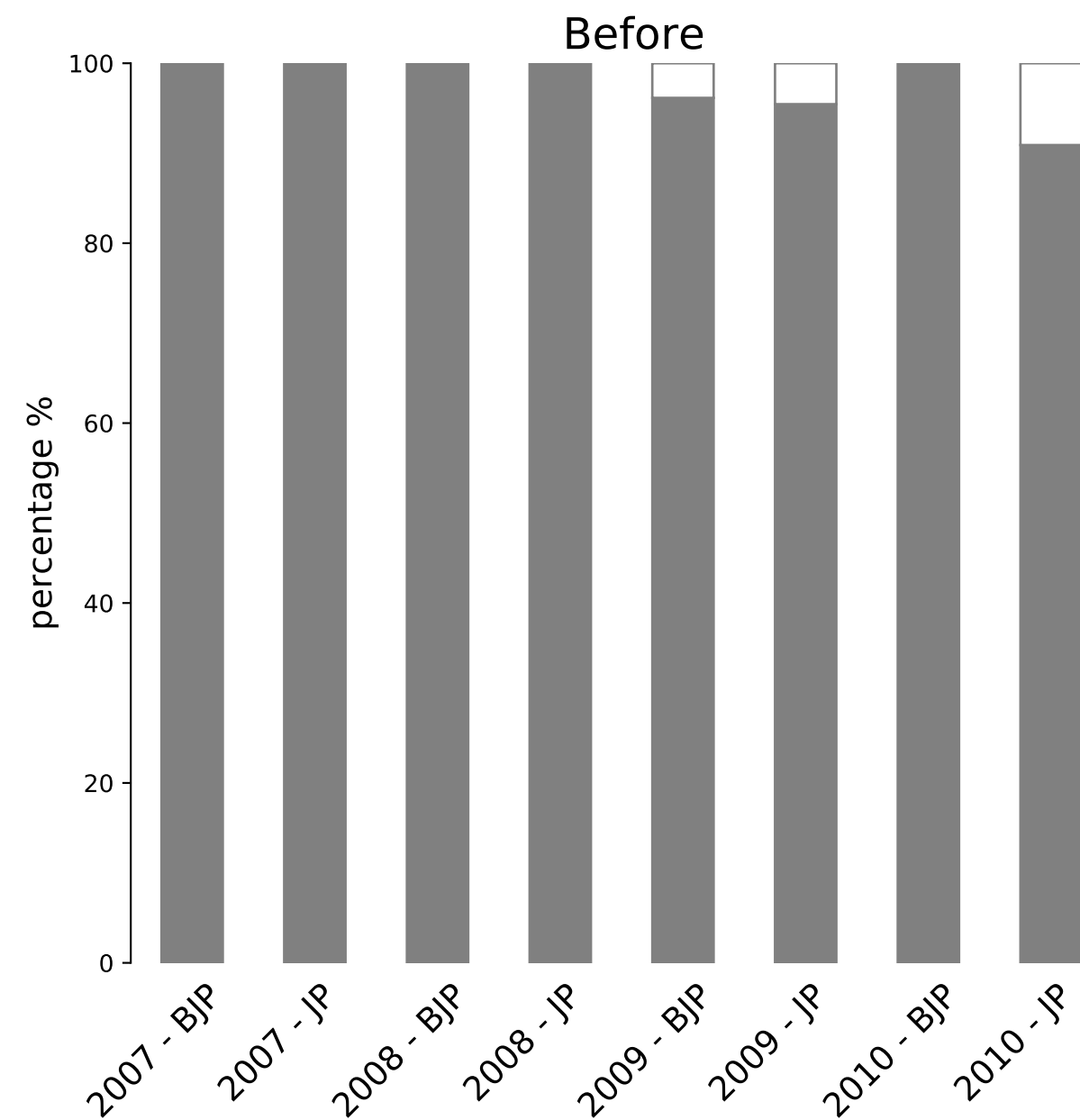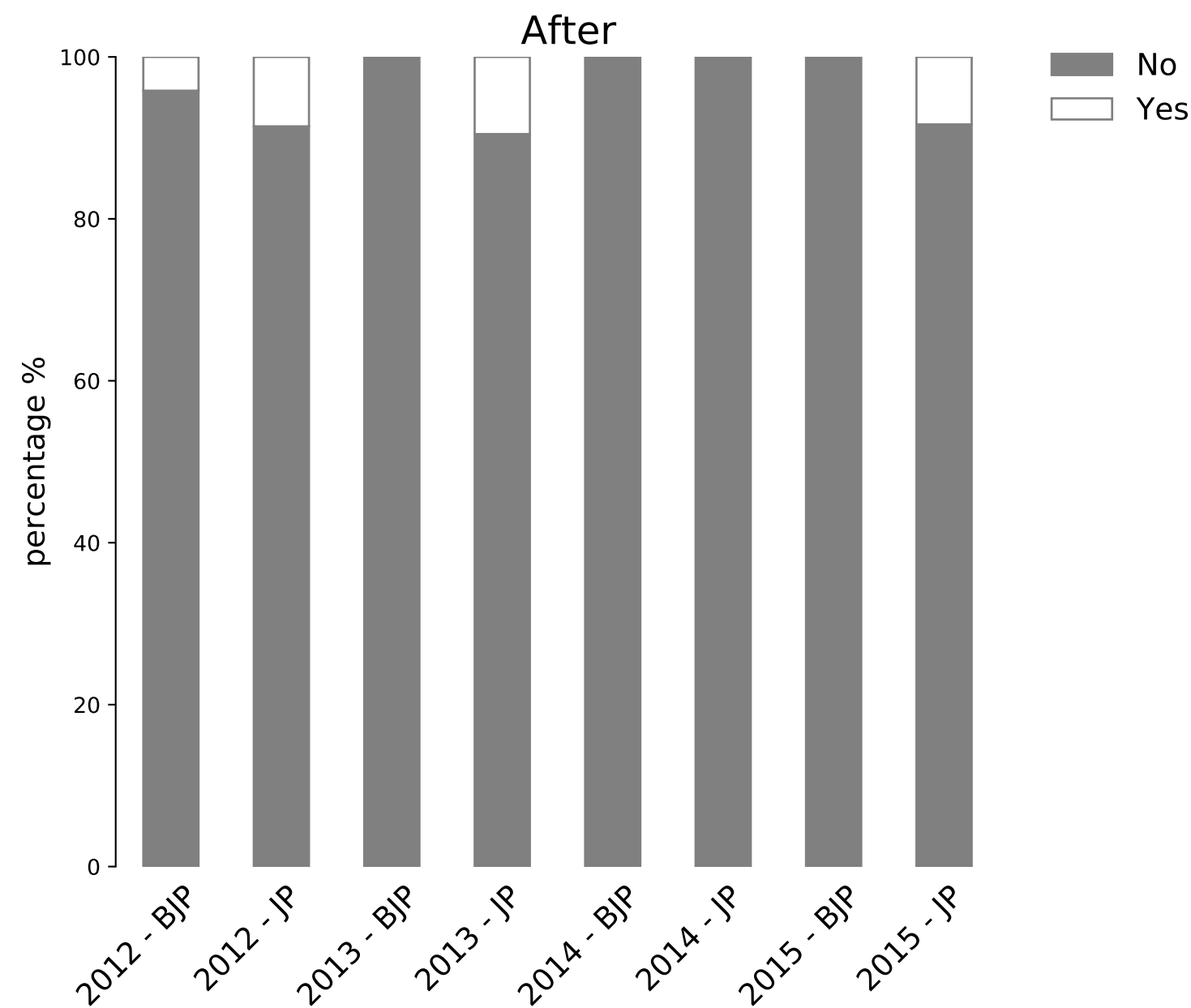

Supplement: S3 File — Comma-separated-values (CSV) file of raw scores for questions 1-10 and the Python files used to analyse the data. See the included README.txt file for a full description. (ZIP) [file pone.0202121.s003.zip › S3 File/supplementary_results/suppl_q8_year_journal.pdf]

Question 7: Are any plotted measures that summarize variability SEM?

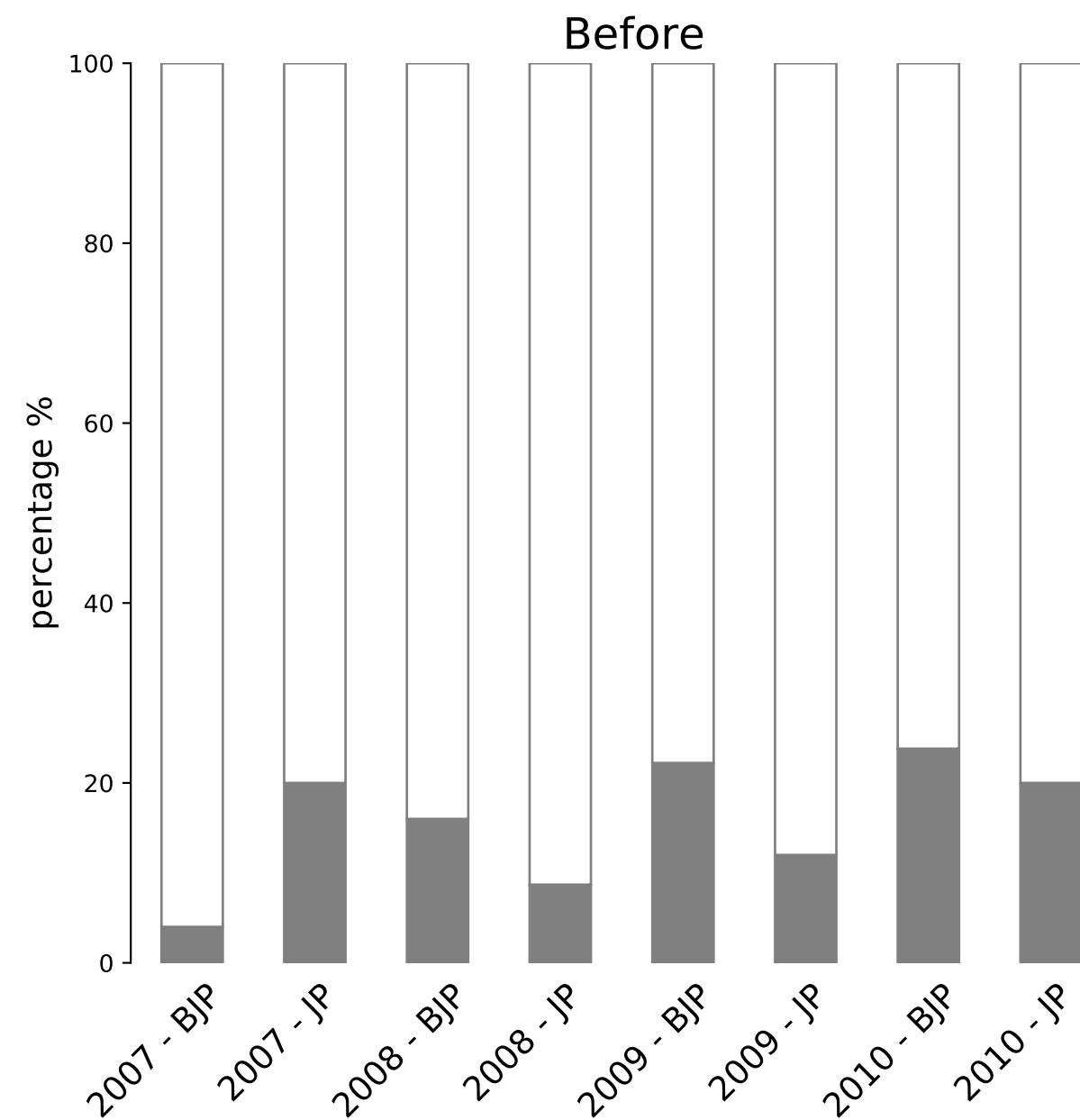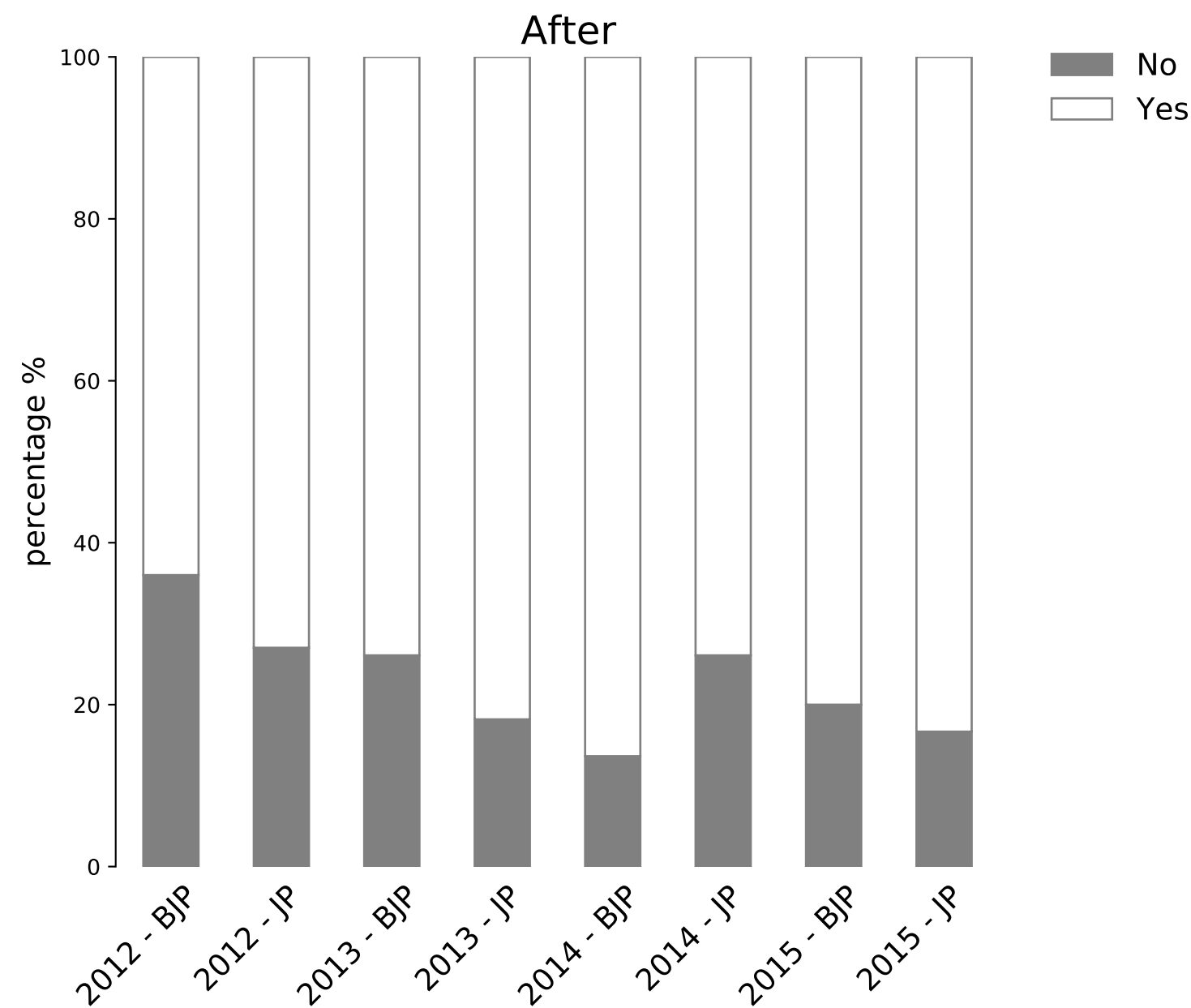

Supplement: S3 File — Comma-separated-values (CSV) file of raw scores for questions 1-10 and the Python files used to analyse the data. See the included README.txt file for a full description. (ZIP) [file pone.0202121.s003.zip › S3 File/supplementary_results/suppl_q7_year_journal.pdf]

## Question 6: Are all plotted measures that summarize variability defined?

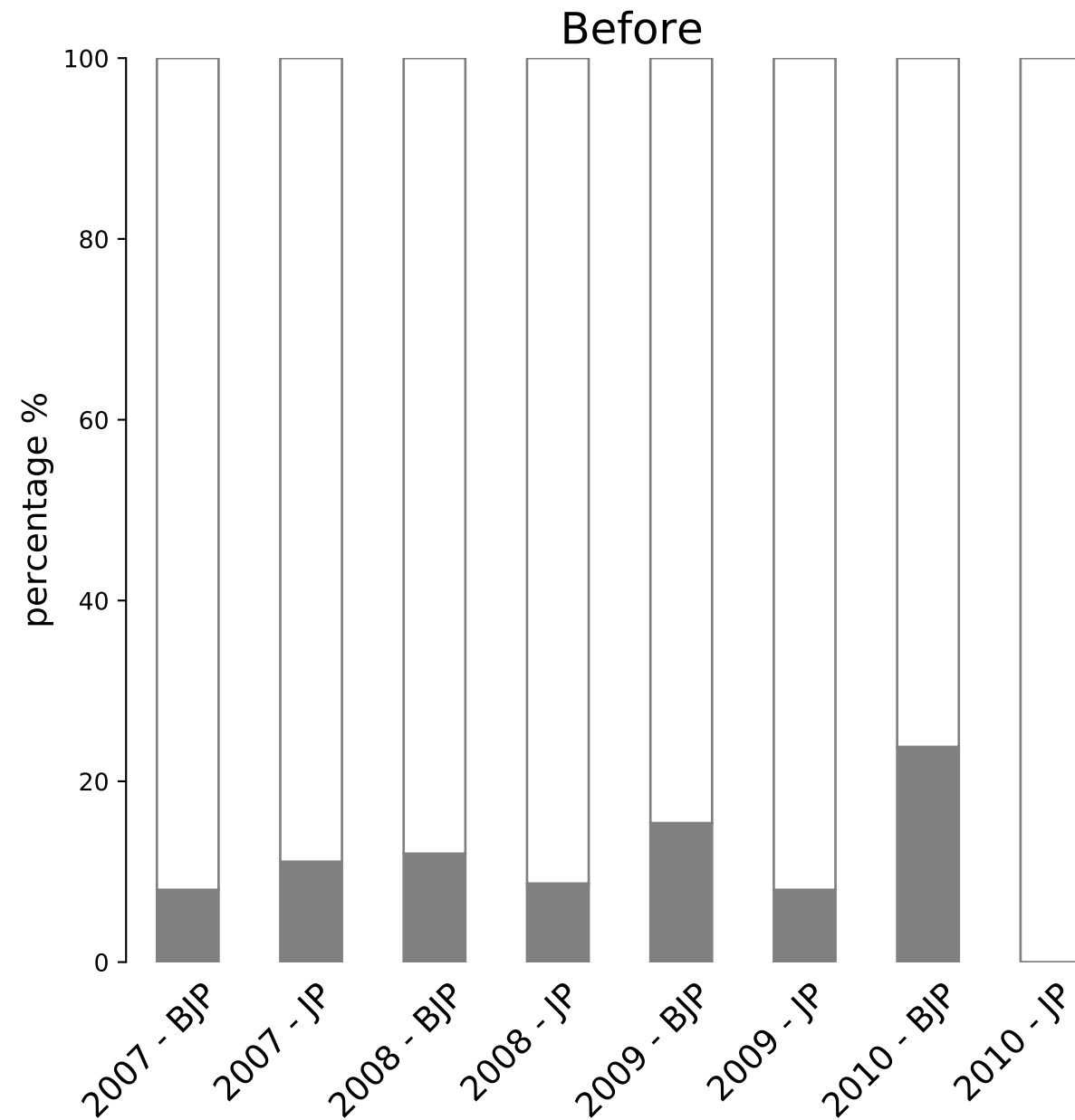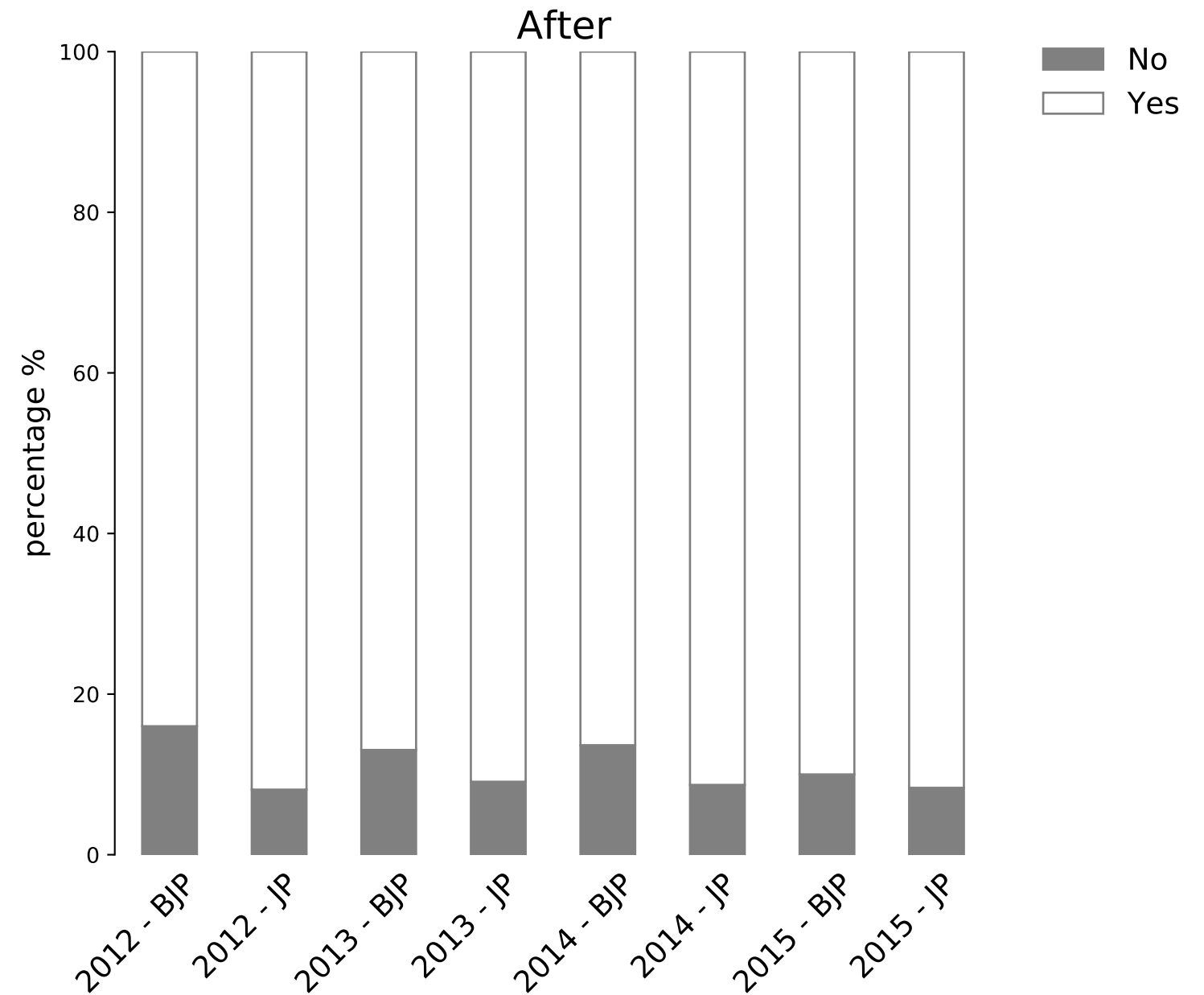

Supplement: S3 File — Comma-separated-values (CSV) file of raw scores for questions 1-10 and the Python files used to analyse the data. See the included README.txt file for a full description. (ZIP) [file pone.0202121.s003.zip › S3 File/supplementary_results/suppl_q6_year_journal.pdf]

## Question 5: Are all reported or implied post-hoc p-values exact?

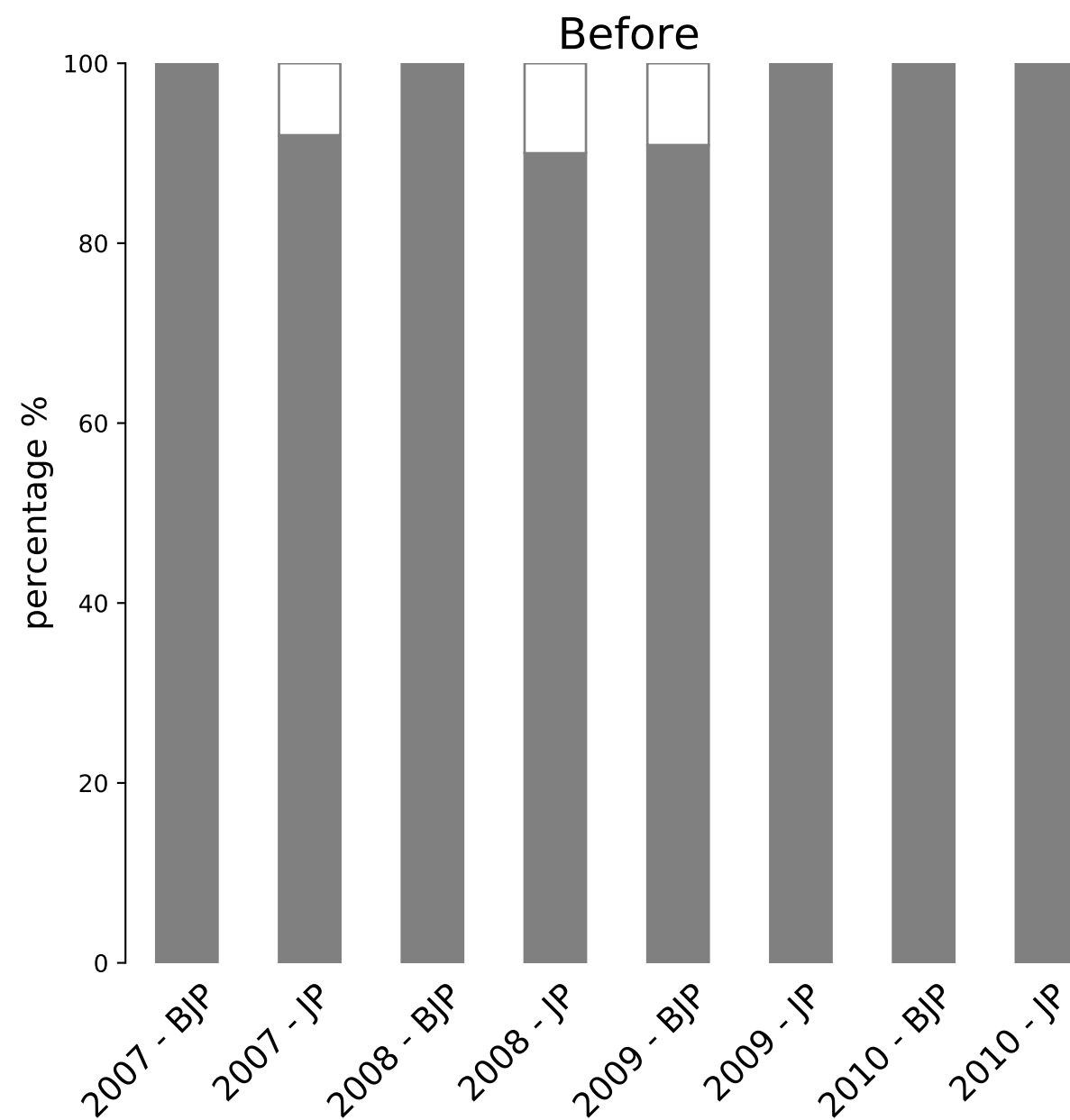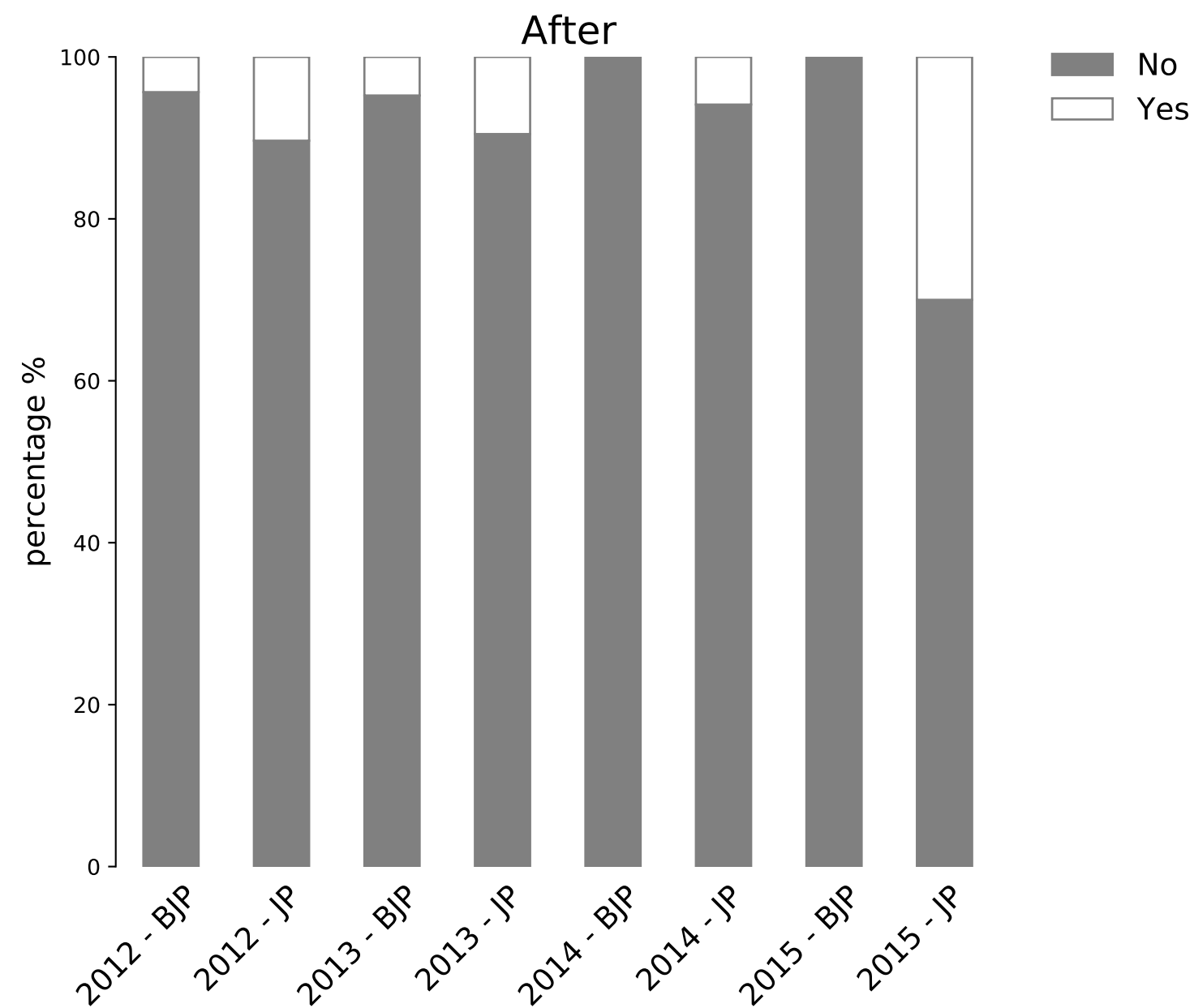

Supplement: S3 File — Comma-separated-values (CSV) file of raw scores for questions 1-10 and the Python files used to analyse the data. See the included README.txt file for a full description. (ZIP) [file pone.0202121.s003.zip › S3 File/supplementary_results/suppl_q5_year_journal.pdf]

### Question 3: Are p-values for all main analyses reported or implied?

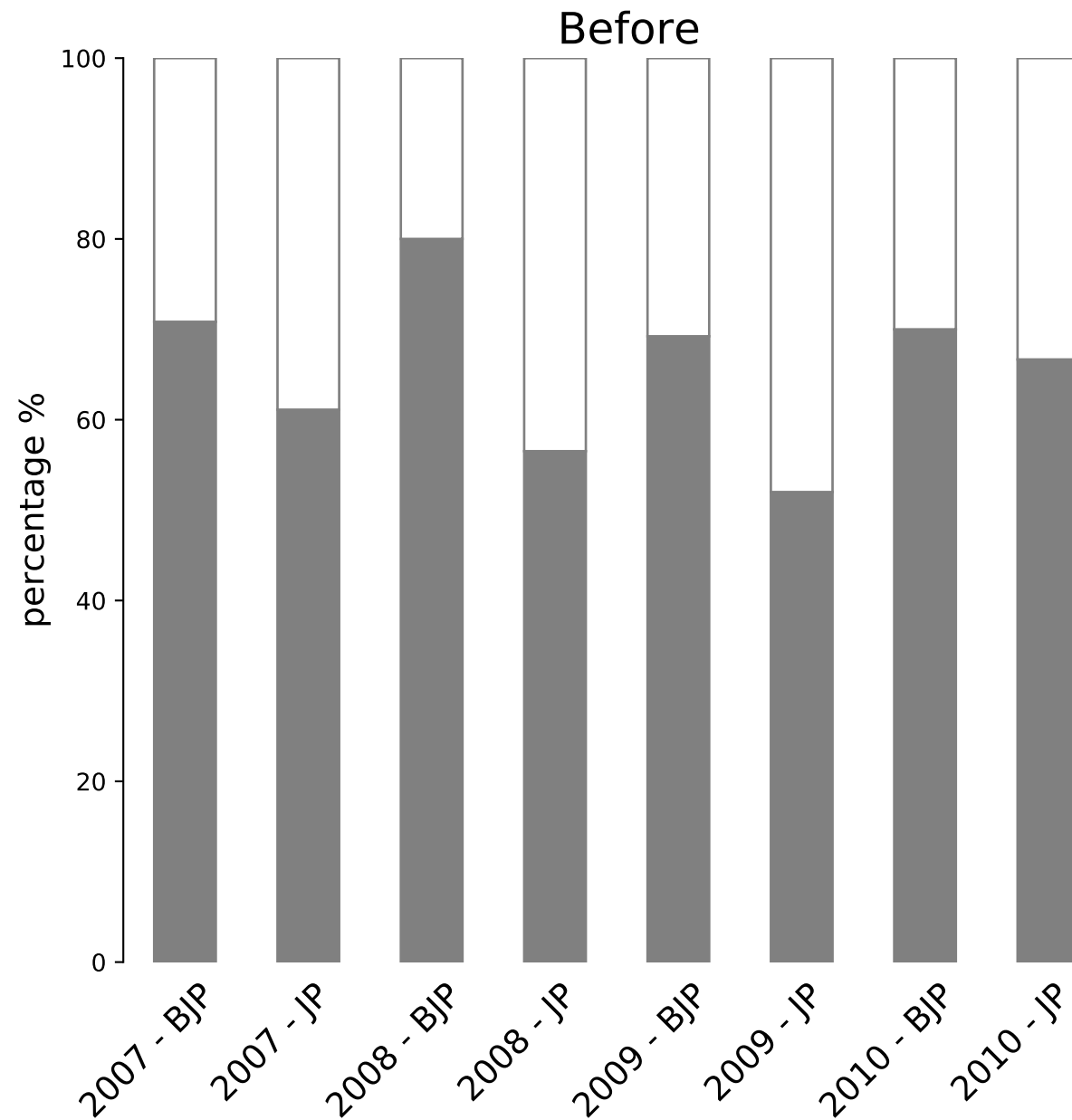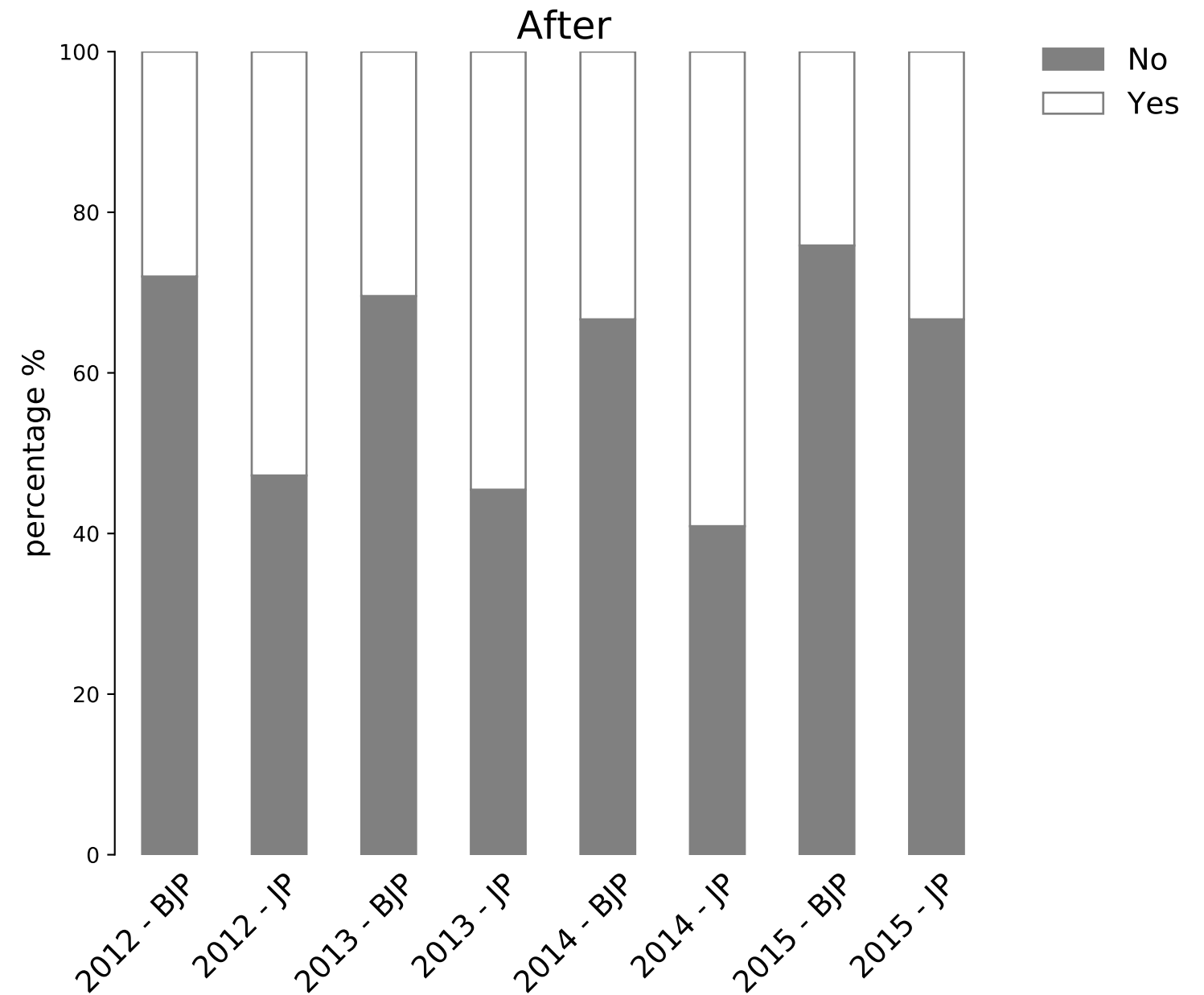

Supplement: S3 File — Comma-separated-values (CSV) file of raw scores for questions 1-10 and the Python files used to analyse the data. See the included README.txt file for a full description. (ZIP) [file pone.0202121.s003.zip › S3 File/supplementary_results/suppl_q3_year_journal.pdf]

## Question 2: Are any written measures that summarize data variability SEM?

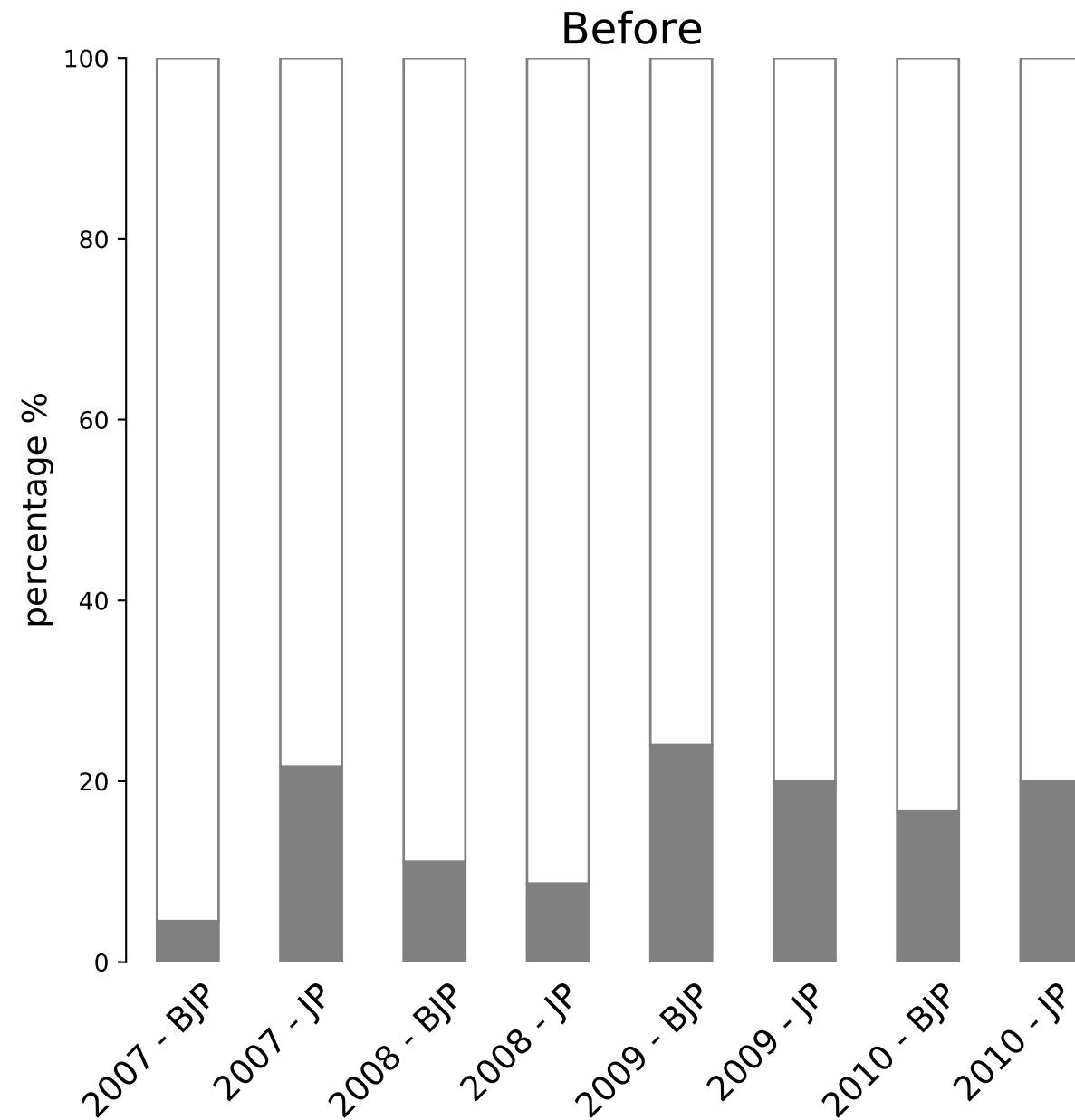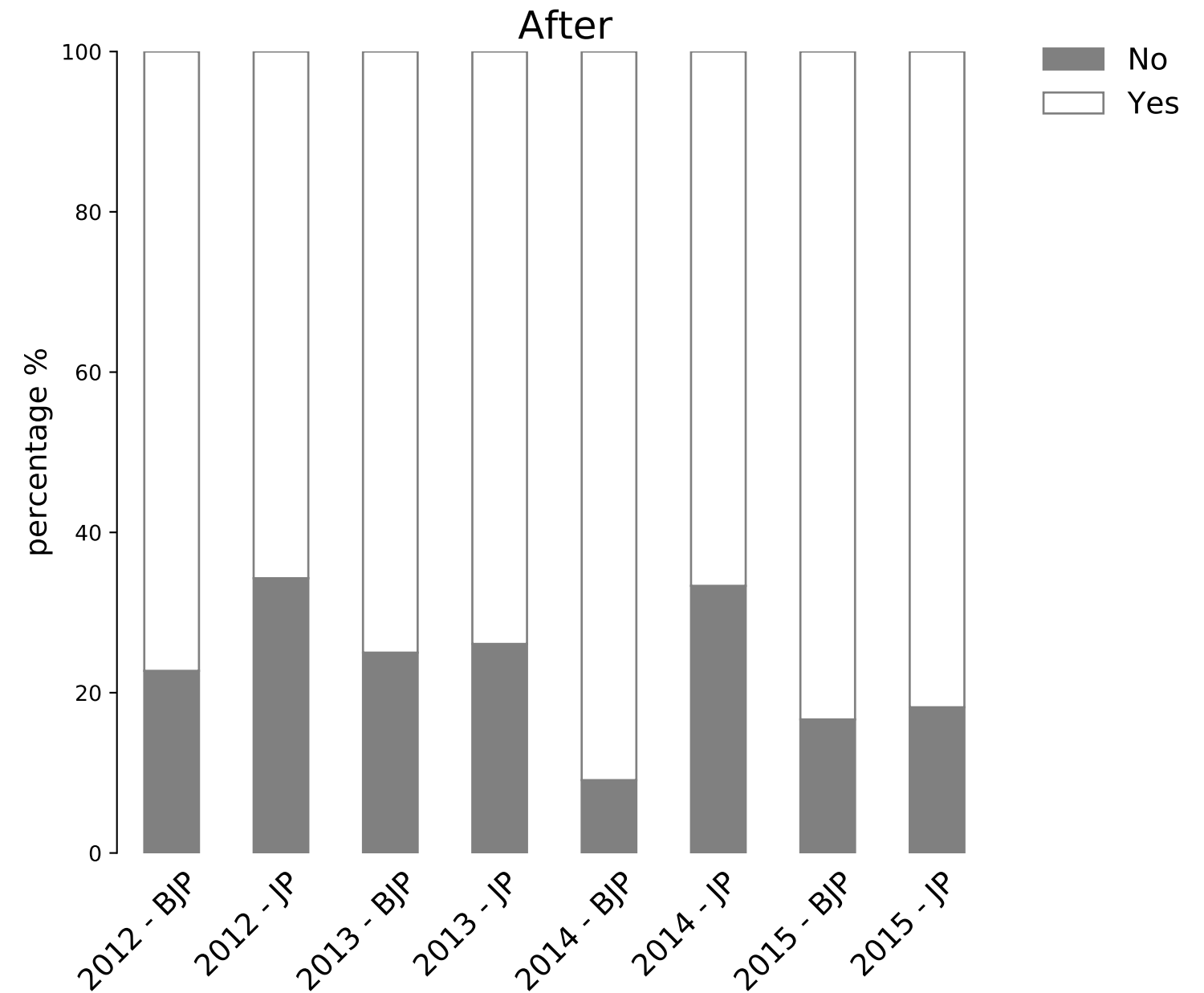

Supplement: S3 File — Comma-separated-values (CSV) file of raw scores for questions 1-10 and the Python files used to analyse the data. See the included README.txt file for a full description. (ZIP) [file pone.0202121.s003.zip › S3 File/supplementary_results/suppl_q2_year_journal.pdf]

# Question 1: Are all written measures that summarize data variability defined?

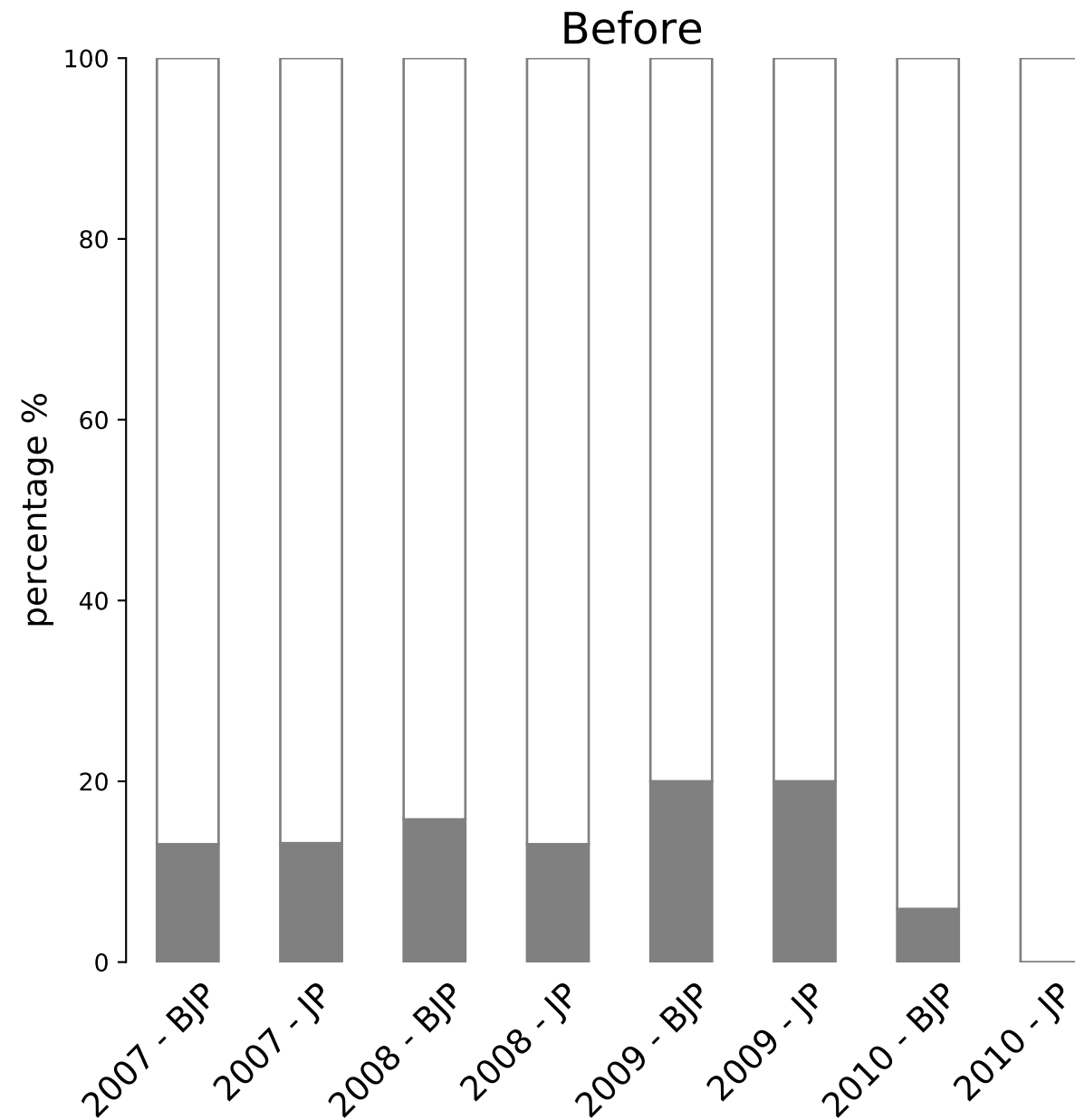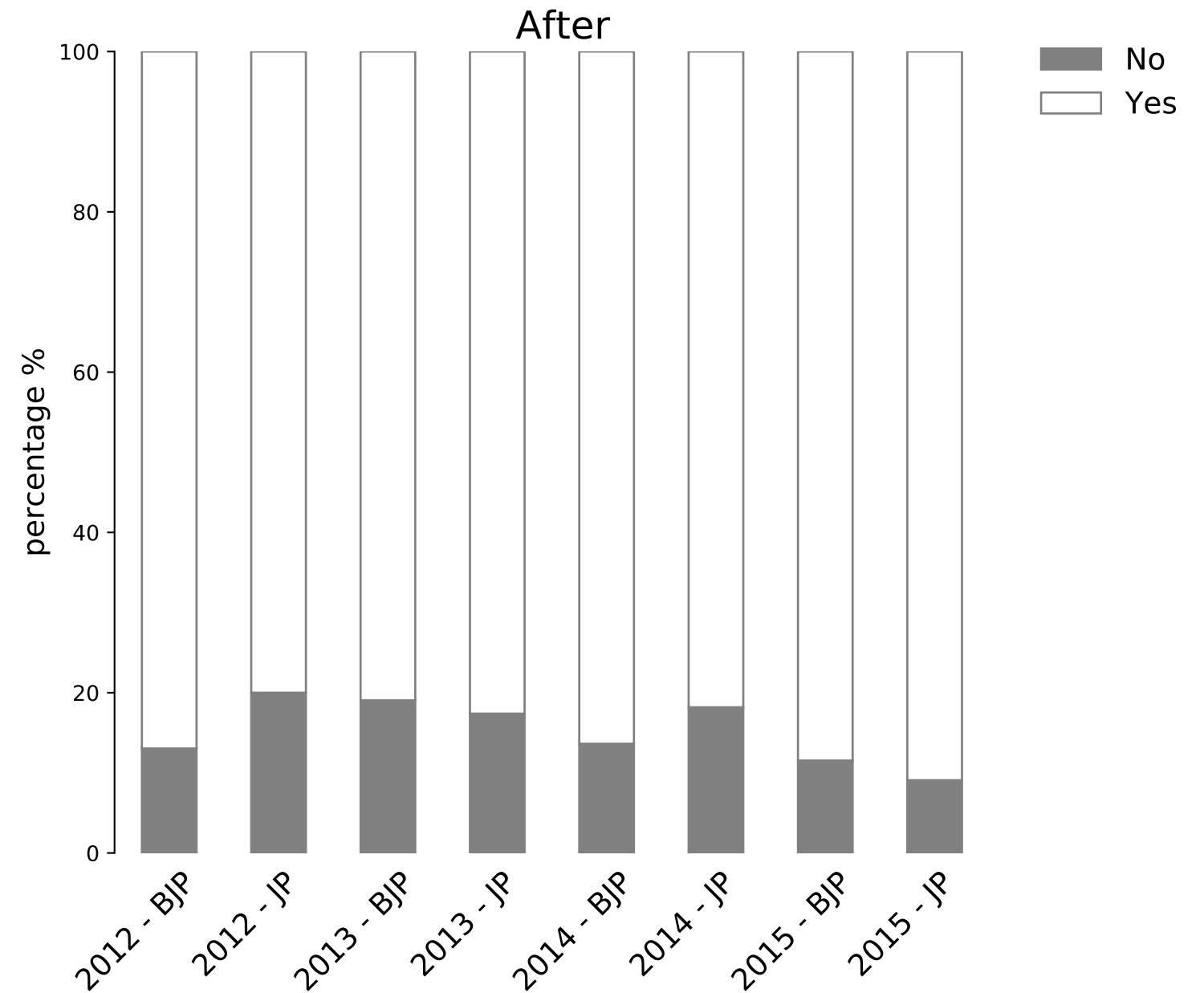

Supplement: S3 File — Comma-separated-values (CSV) file of raw scores for questions 1-10 and the Python files used to analyse the data. See the included README.txt file for a full description. (ZIP) [file pone.0202121.s003.zip › S3 File/supplementary_results/suppl_q1_year_journal.pdf]

Question 10: Are any p-values between 0.05-0.1 interpreted as trends/significance?

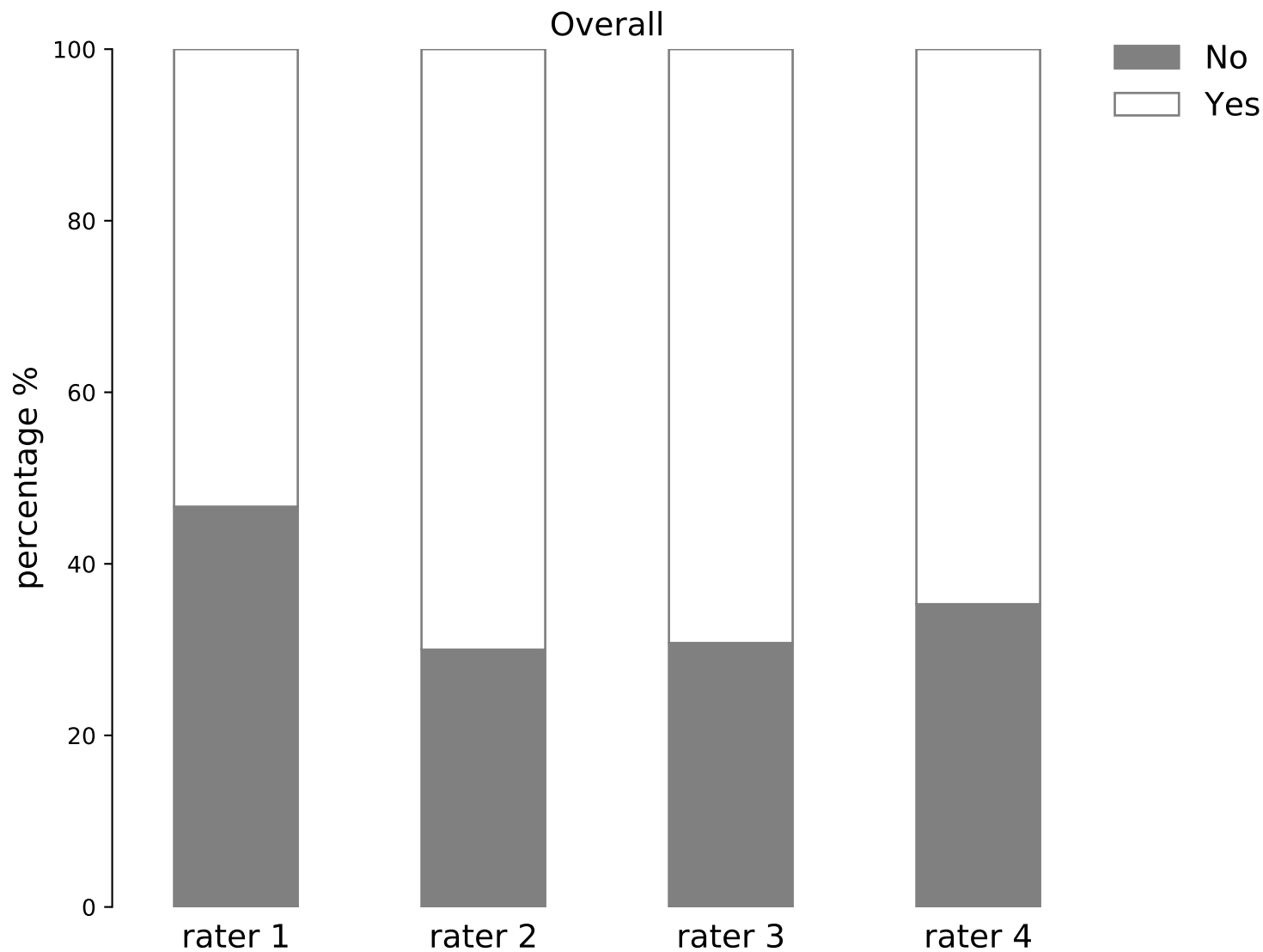

Supplement: S3 File — Comma-separated-values (CSV) file of raw scores for questions 1-10 and the Python files used to analyse the data. See the included README.txt file for a full description. (ZIP) [file pone.0202121.s003.zip › S3 File/supplementary_results/suppl_q10_rater.pdf]

Question 9: Does the paper report any exact p-values that are between 0.05-0.1?

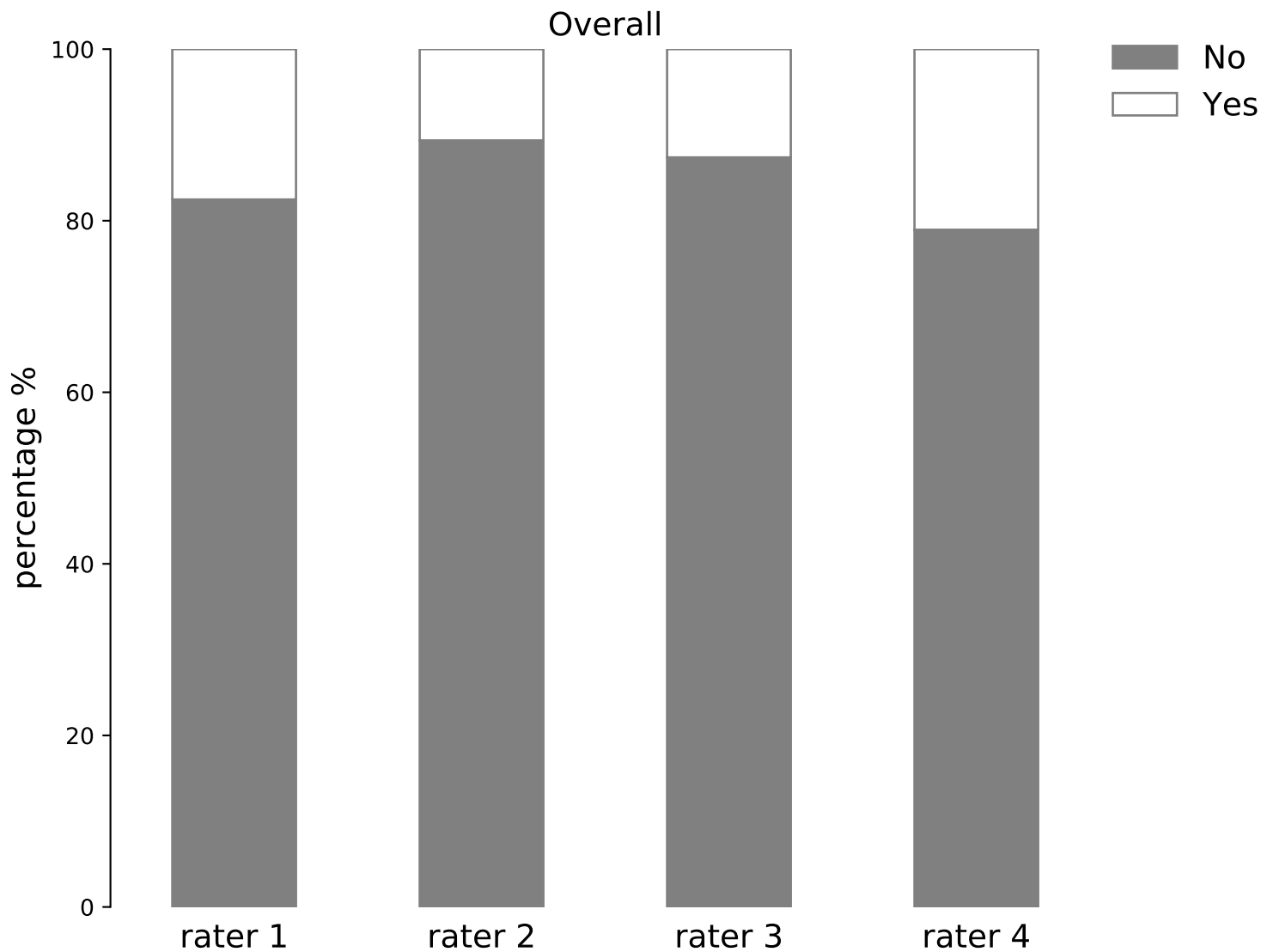

Supplement: S3 File — Comma-separated-values (CSV) file of raw scores for questions 1-10 and the Python files used to analyse the data. See the included README.txt file for a full description. (ZIP) [file pone.0202121.s003.zip › S3 File/supplementary_results/suppl_q9_rater.pdf]

Question 8: For all figures that summarize data/variability, are raw data plotted?

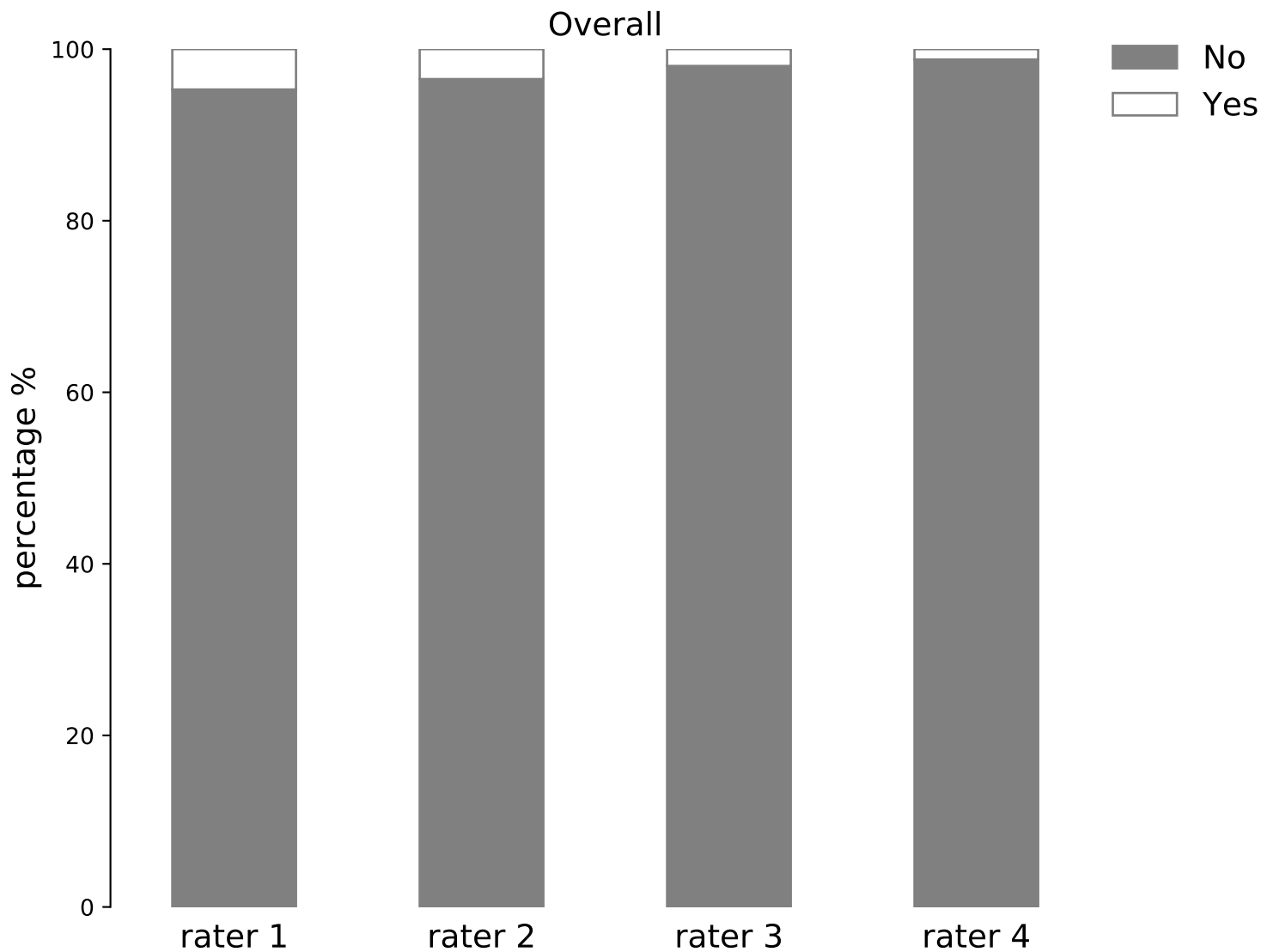

Supplement: S3 File — Comma-separated-values (CSV) file of raw scores for questions 1-10 and the Python files used to analyse the data. See the included README.txt file for a full description. (ZIP) [file pone.0202121.s003.zip › S3 File/supplementary_results/suppl_q8_rater.pdf]

Question 7: Are any plotted measures that summarize variability SEM?

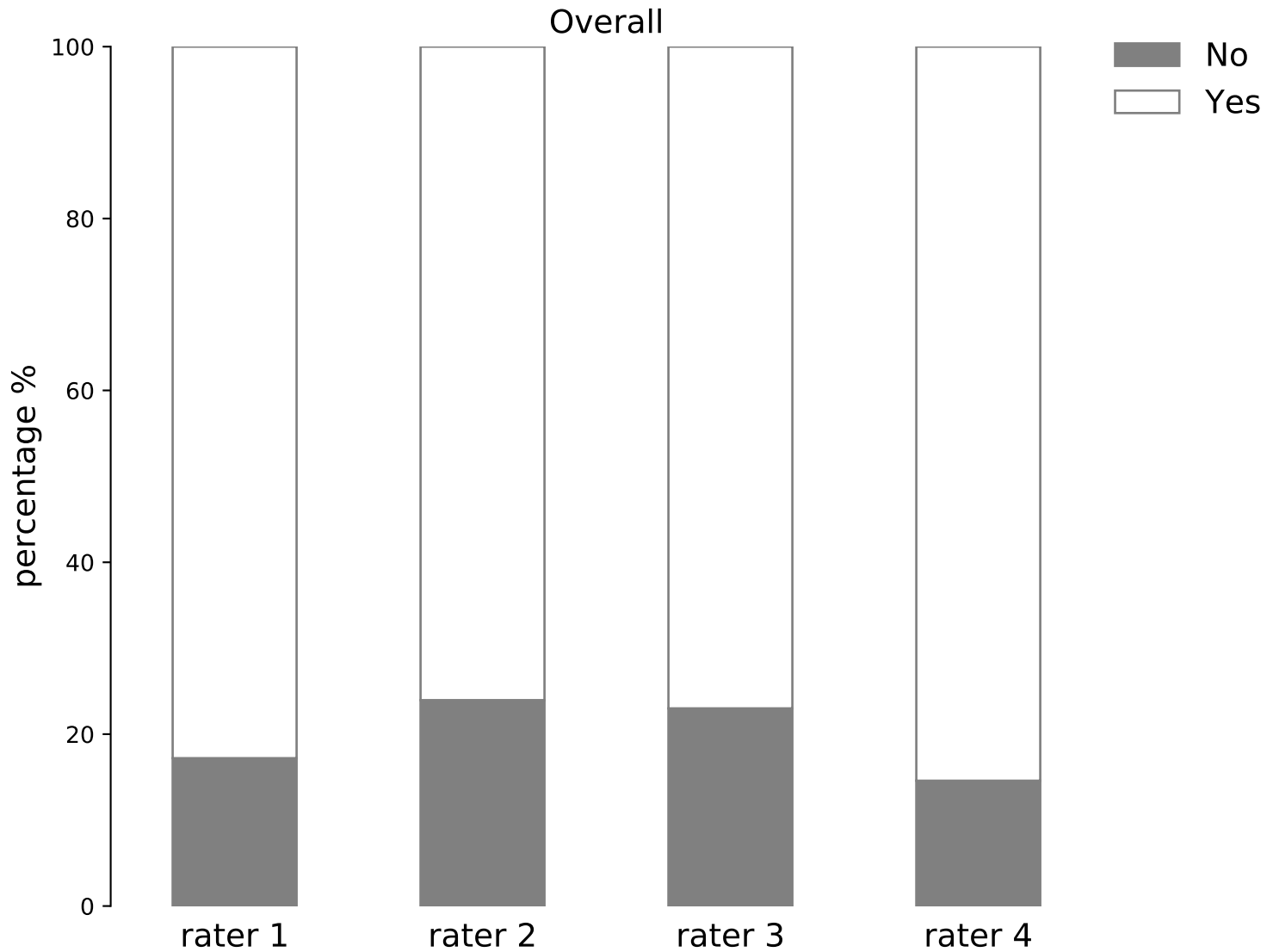

Supplement: S3 File — Comma-separated-values (CSV) file of raw scores for questions 1-10 and the Python files used to analyse the data. See the included README.txt file for a full description. (ZIP) [file pone.0202121.s003.zip › S3 File/supplementary_results/suppl_q7_rater.pdf]

Question 6: Are all plotted measures that summarize variability defined?

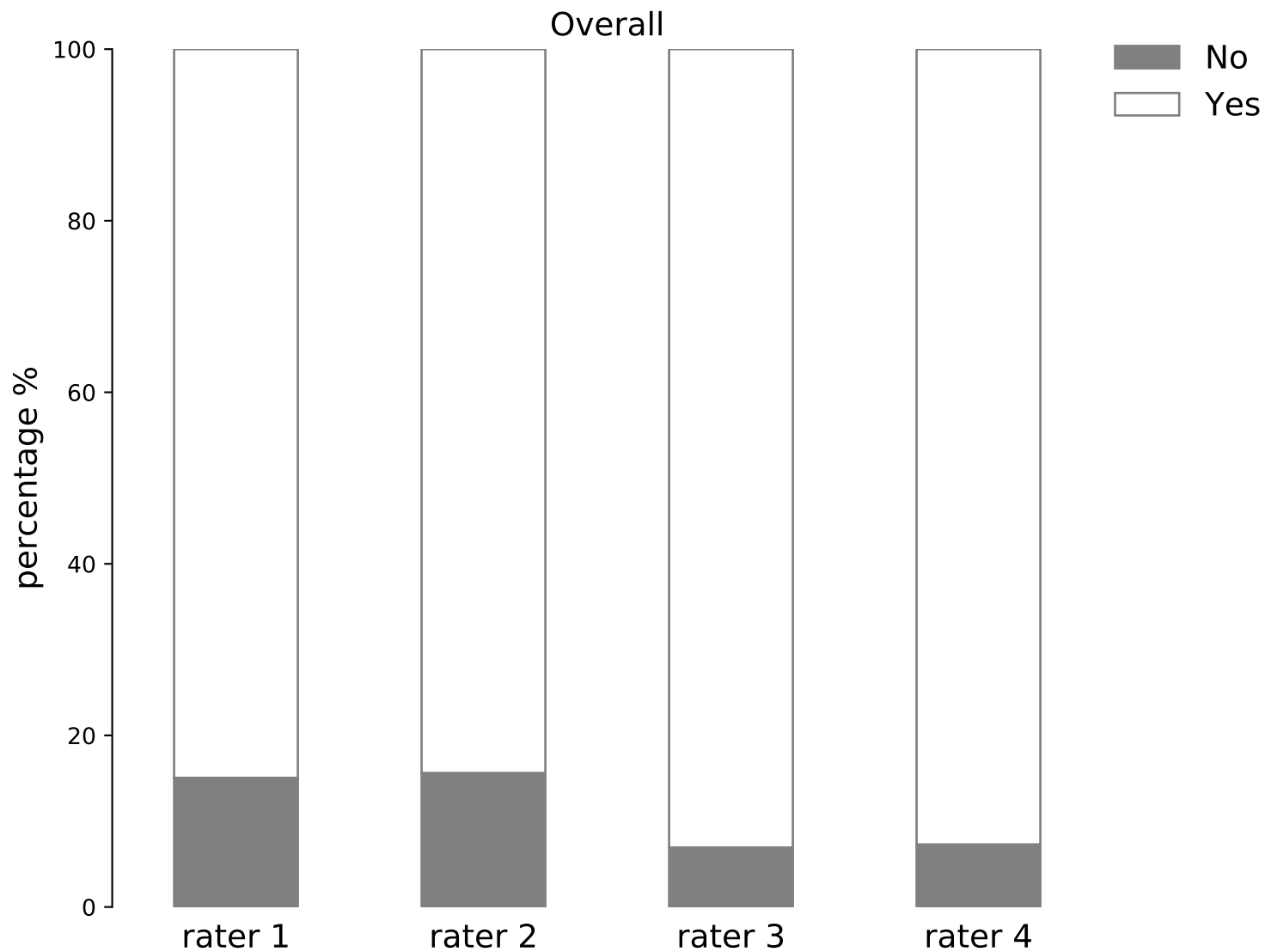

Supplement: S3 File — Comma-separated-values (CSV) file of raw scores for questions 1-10 and the Python files used to analyse the data. See the included README.txt file for a full description. (ZIP) [file pone.0202121.s003.zip › S3 File/supplementary_results/suppl_q6_rater.pdf]

Question 5: Are all reported or implied post-hoc p-values exact?

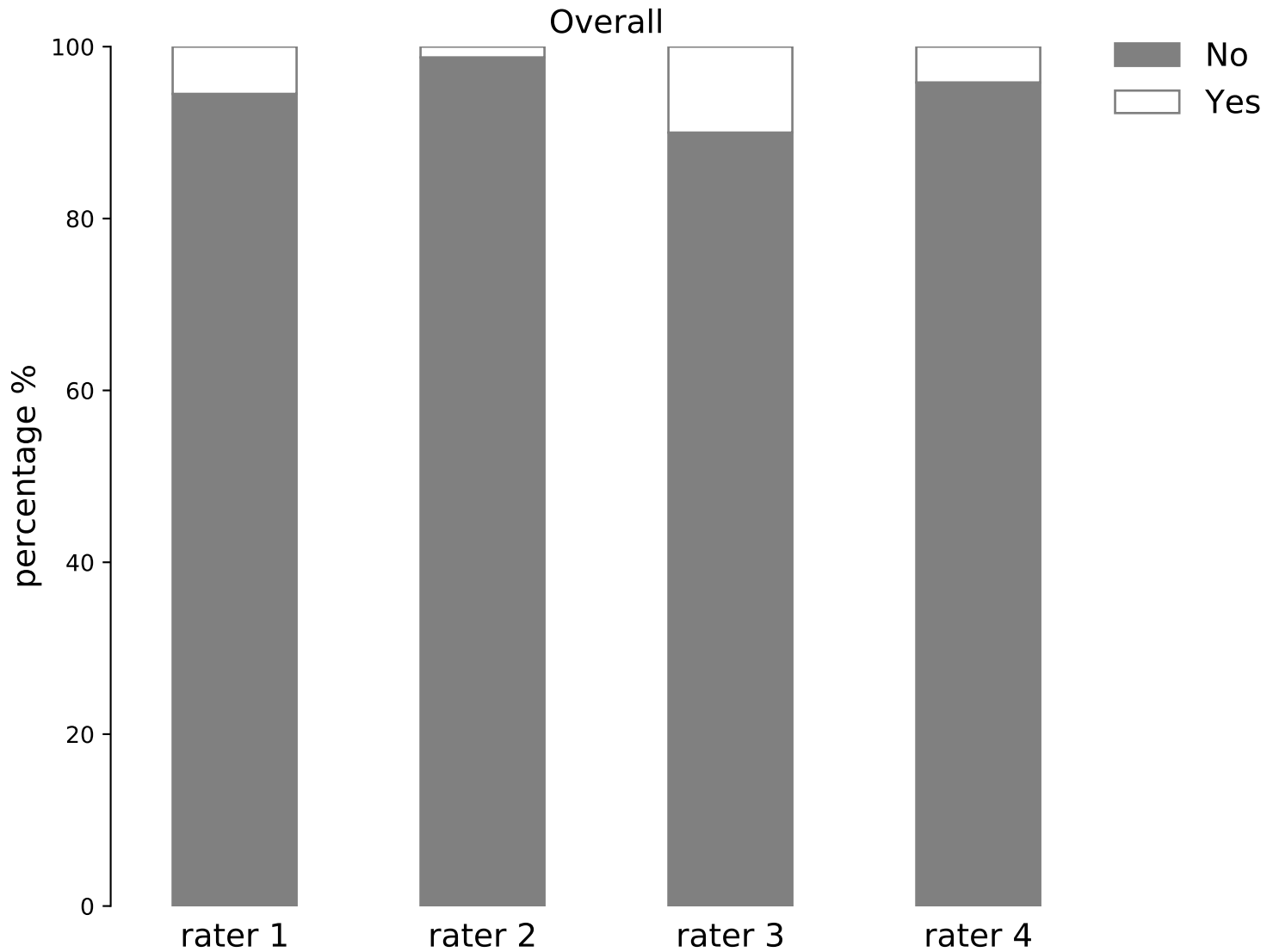

Supplement: S3 File — Comma-separated-values (CSV) file of raw scores for questions 1-10 and the Python files used to analyse the data. See the included README.txt file for a full description. (ZIP) [file pone.0202121.s003.zip › S3 File/supplementary_results/suppl_q5_rater.pdf]

Question 4: Are reported or implied p-values exact for main analyses?

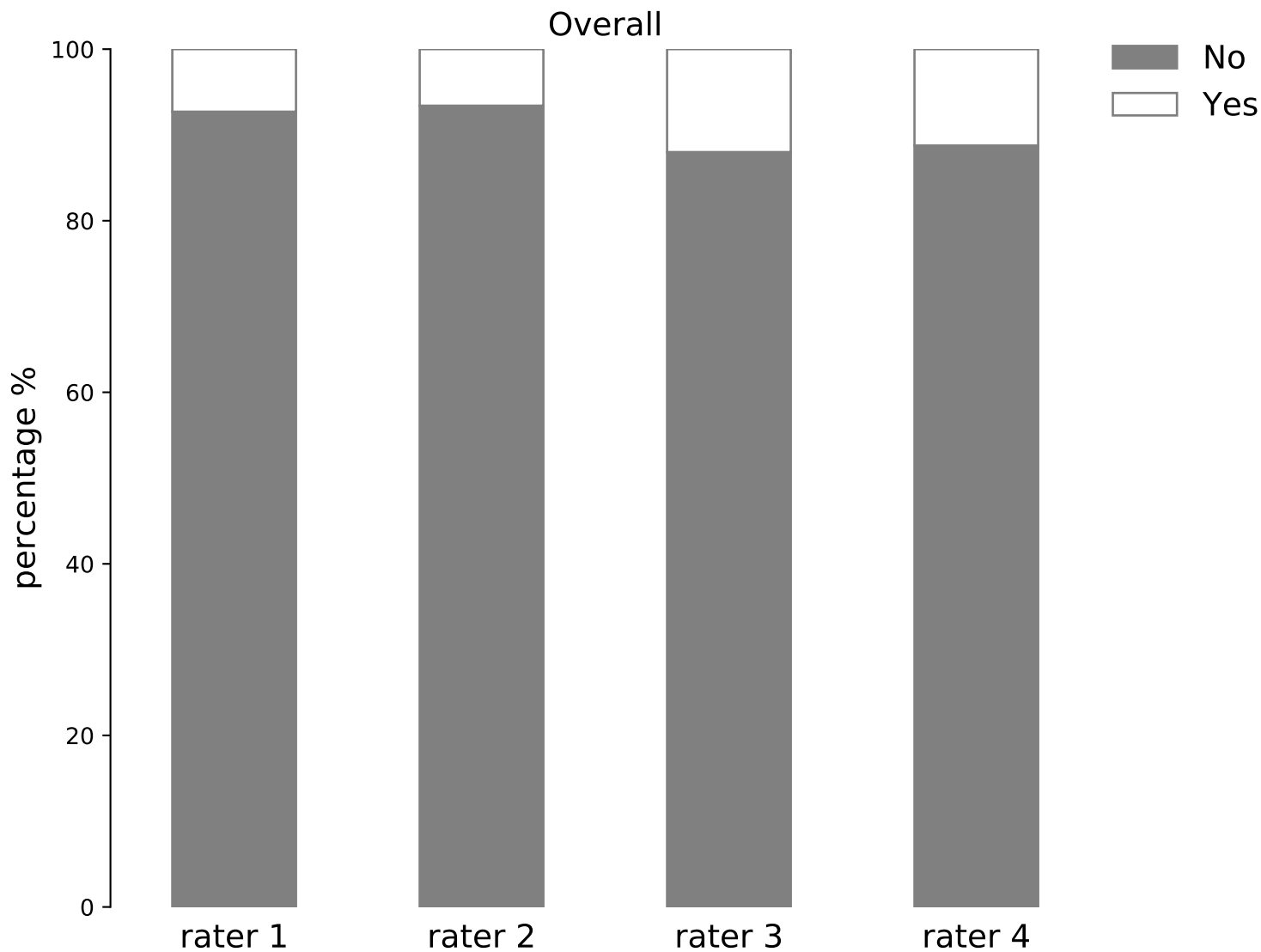

Supplement: S3 File — Comma-separated-values (CSV) file of raw scores for questions 1-10 and the Python files used to analyse the data. See the included README.txt file for a full description. (ZIP) [file pone.0202121.s003.zip › S3 File/supplementary_results/suppl_q4_rater.pdf]

Question 3: Are p-values for all main analyses reported or implied?

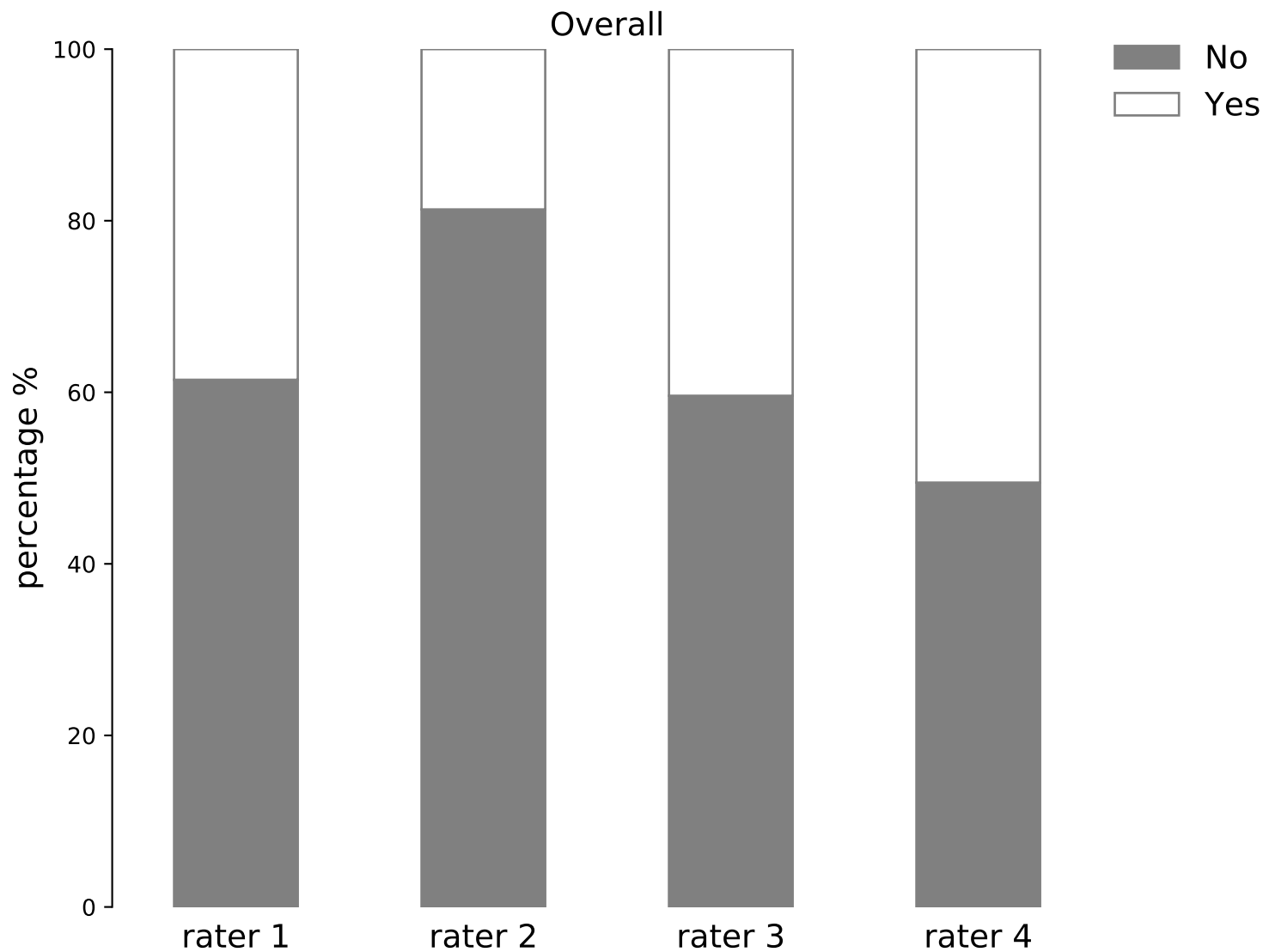

Supplement: S3 File — Comma-separated-values (CSV) file of raw scores for questions 1-10 and the Python files used to analyse the data. See the included README.txt file for a full description. (ZIP) [file pone.0202121.s003.zip › S3 File/supplementary_results/suppl_q3_rater.pdf]

Question 2: Are any written measures that summarize data variability SEM?

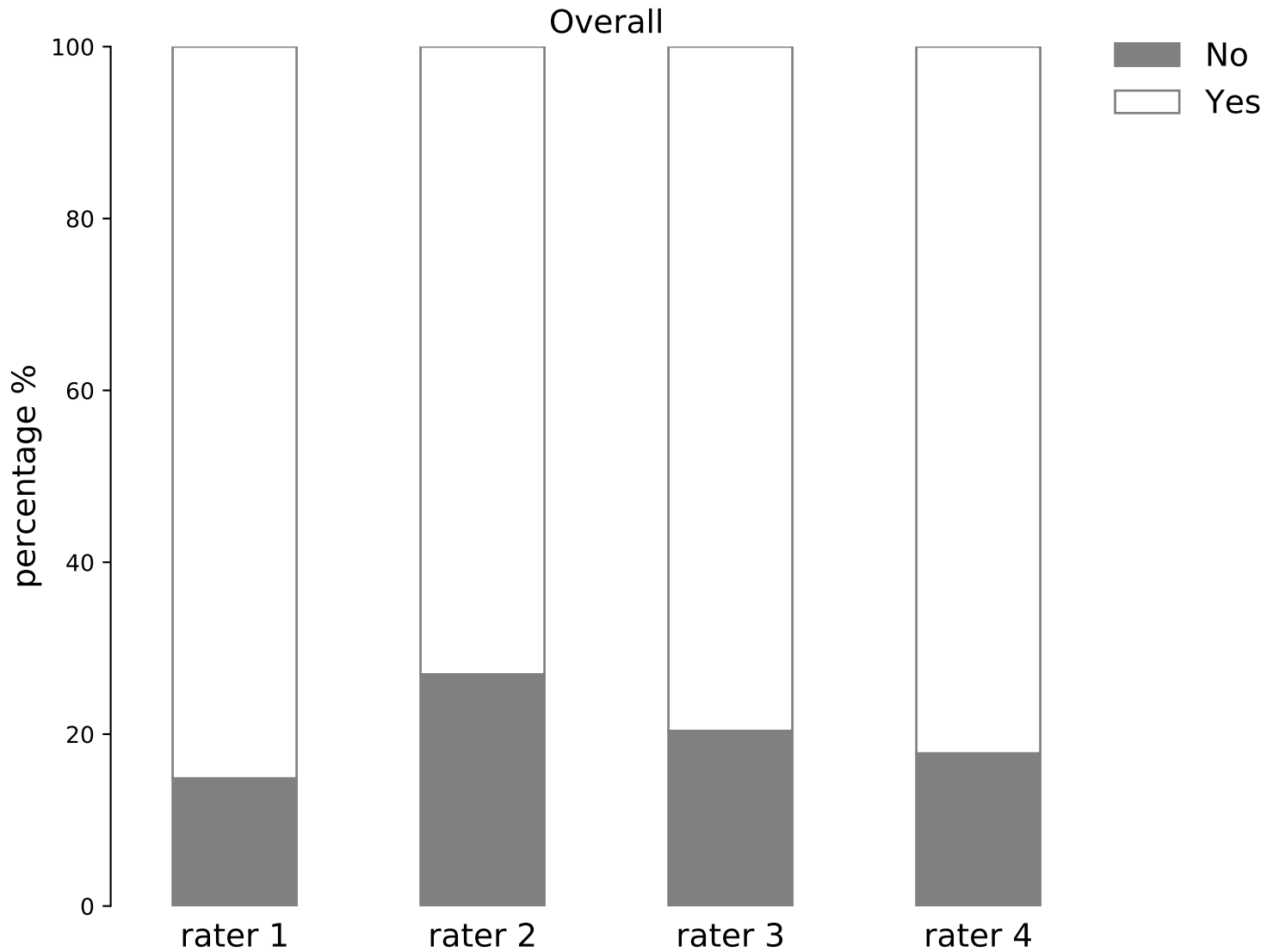

Supplement: S3 File — Comma-separated-values (CSV) file of raw scores for questions 1-10 and the Python files used to analyse the data. See the included README.txt file for a full description. (ZIP) [file pone.0202121.s003.zip › S3 File/supplementary_results/suppl_q2_rater.pdf]

# Question 4: Are reported or implied p-values exact for main analyses?

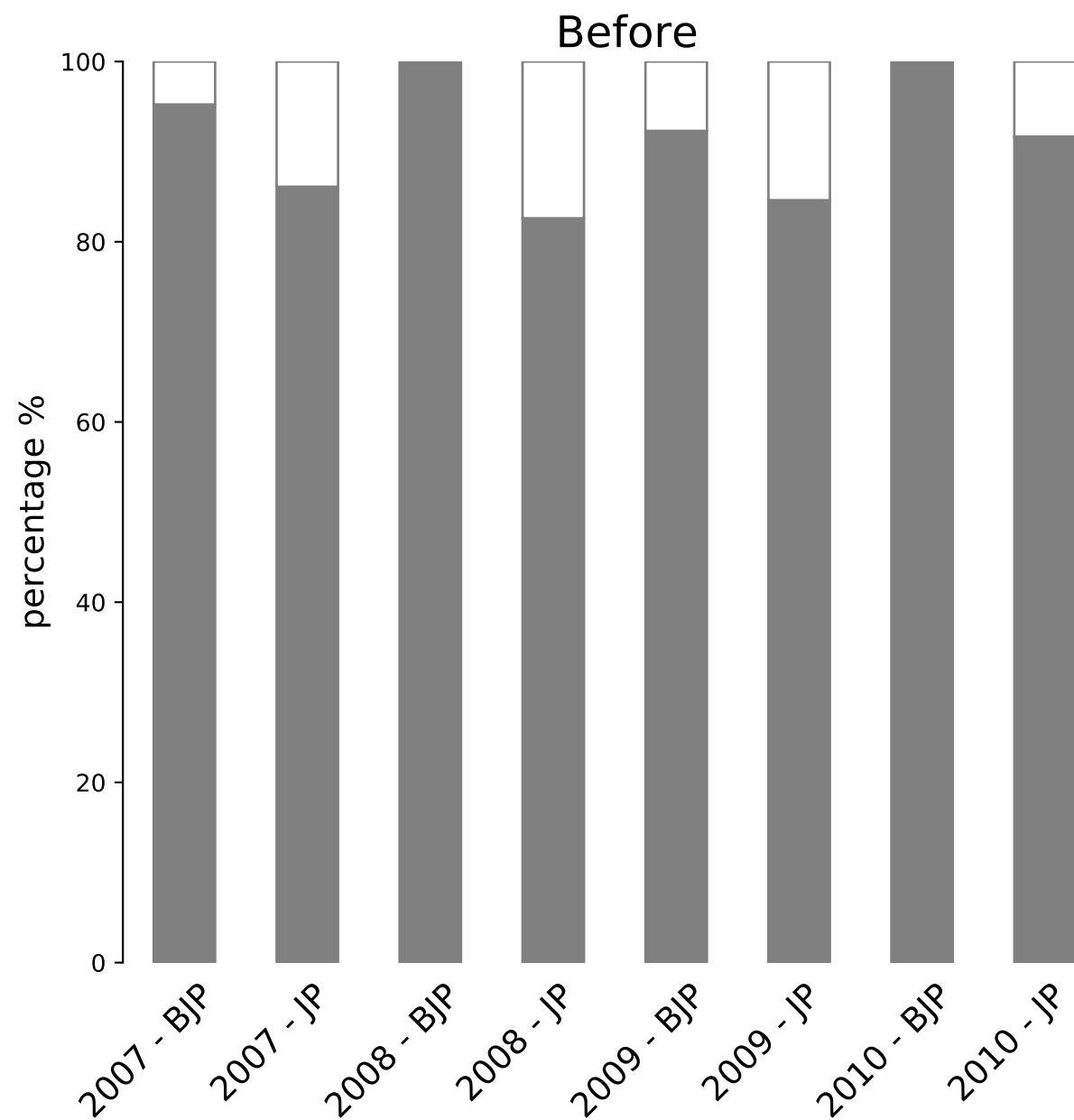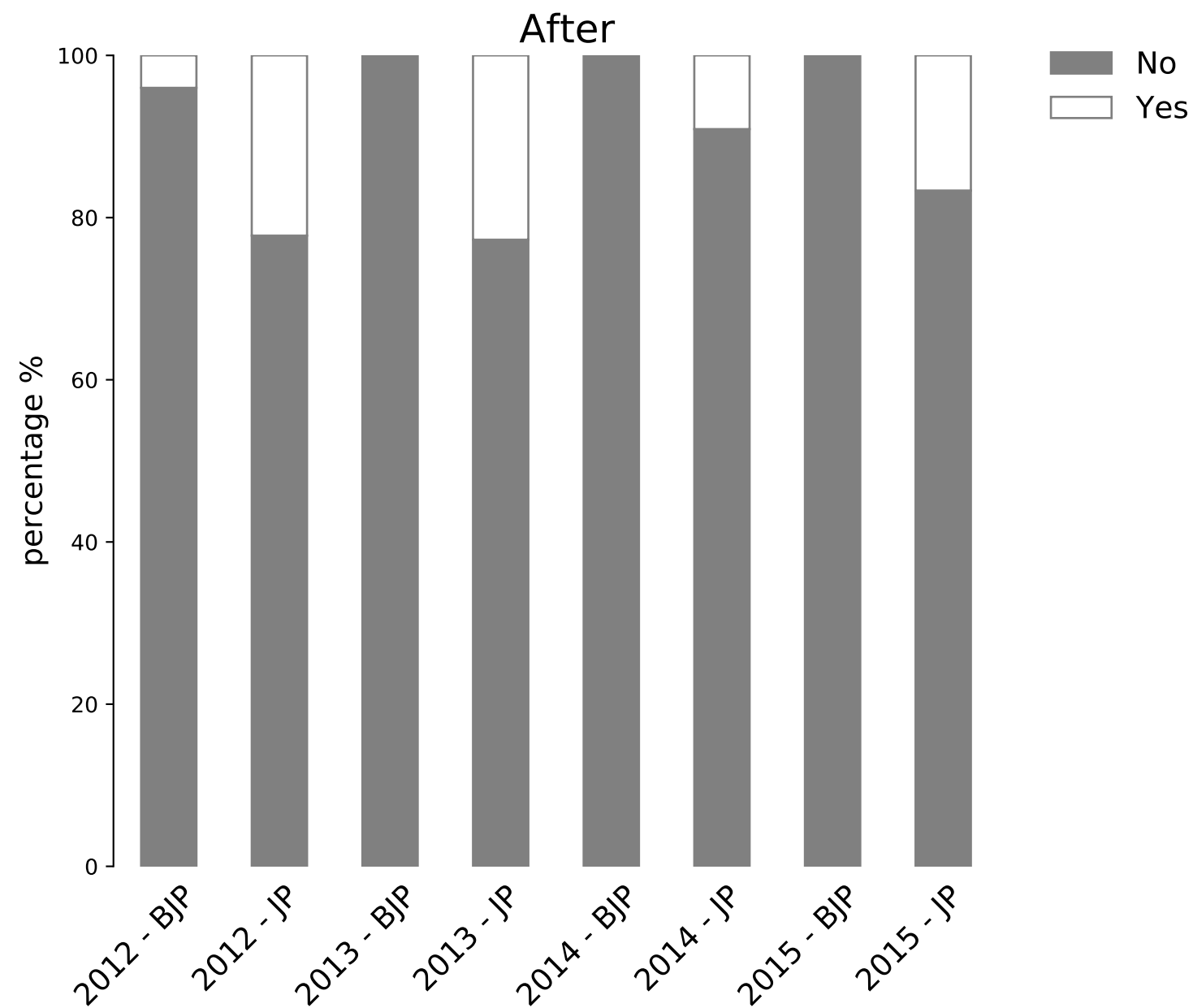

Supplement: S3 File — Comma-separated-values (CSV) file of raw scores for questions 1-10 and the Python files used to analyse the data. See the included README.txt file for a full description. (ZIP) [file pone.0202121.s003.zip › S3 File/supplementary_results/suppl_q4_year_journal.pdf]

Question 1: Are all written measures that summarize data variability defined?

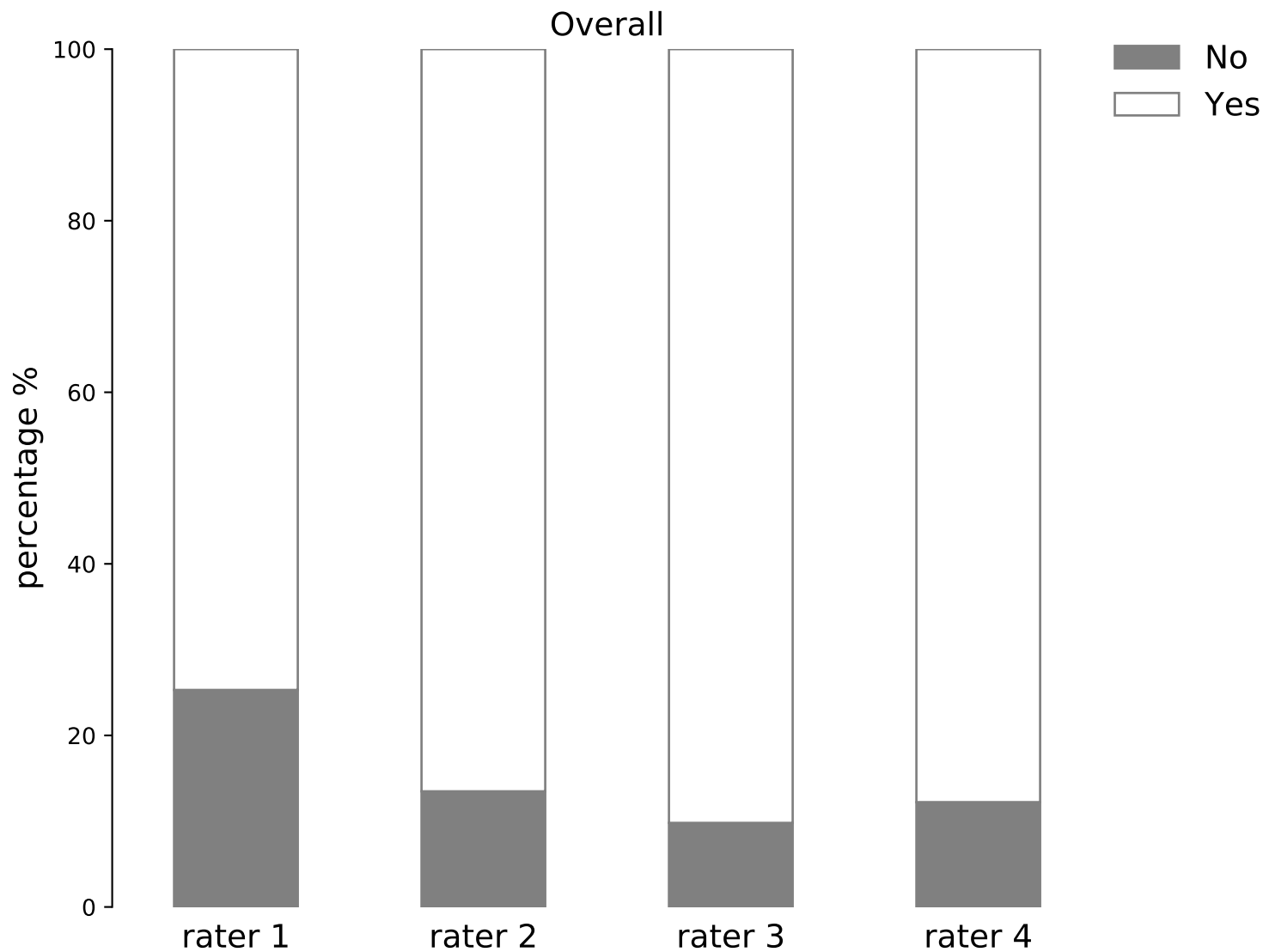

Supplement: S3 File — Comma-separated-values (CSV) file of raw scores for questions 1-10 and the Python files used to analyse the data. See the included README.txt file for a full description. (ZIP) [file pone.0202121.s003.zip › S3 File/supplementary_results/suppl_q1_rater.pdf]
