# Supplementary material for: Poor statistical reporting, inadequate data presentation and spin persist despite editorial advice
Source: PLoS One. 2018 Aug 15;13(8):e0202121. doi: 10.1371/journal.pone.0202121 (PMC6093658; doi:10.1371/journal.pone.0202121)

Question 1: Are all written measures that summarize data variability defined?

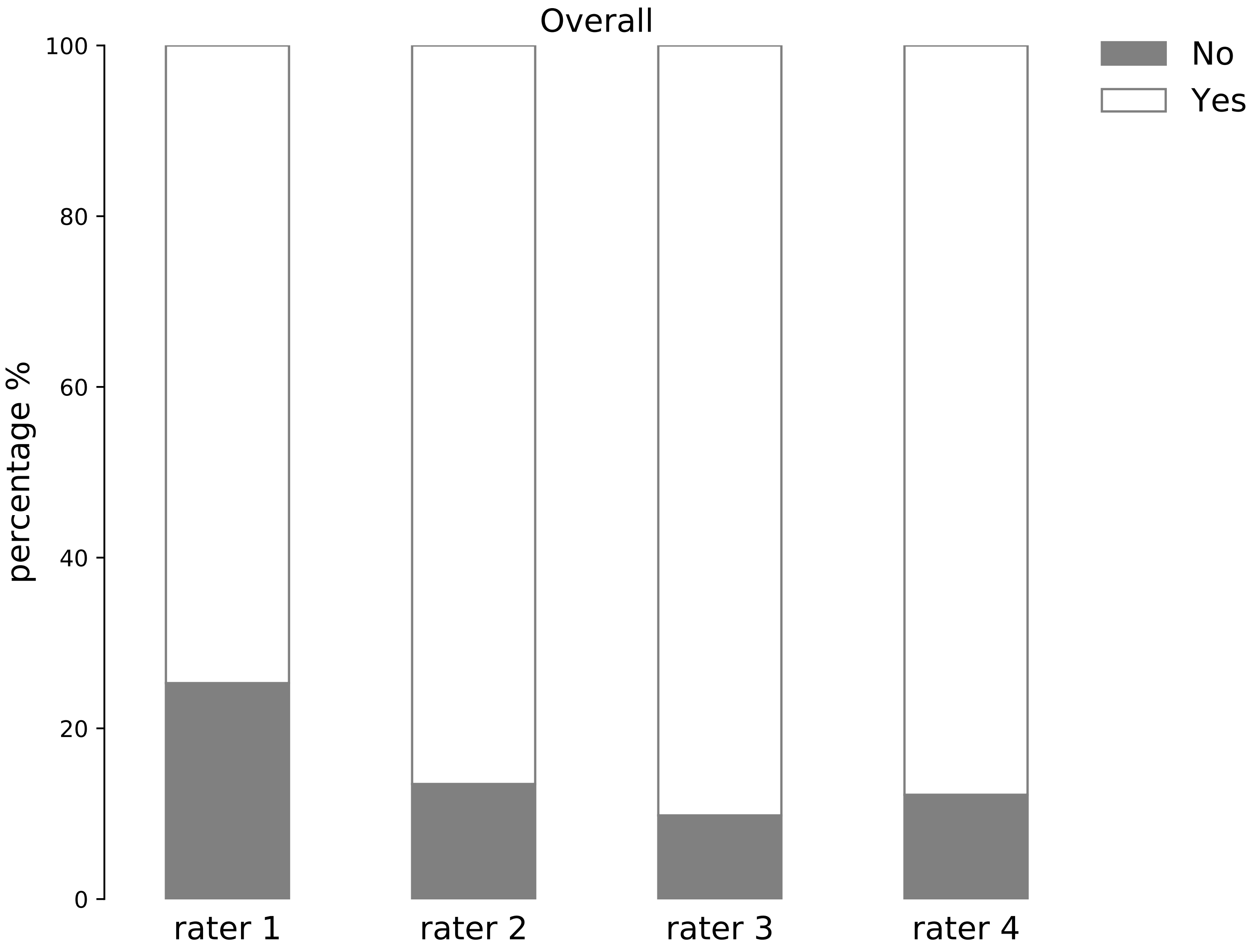

Question 2: Are any written measures that summarize data variability SEM?

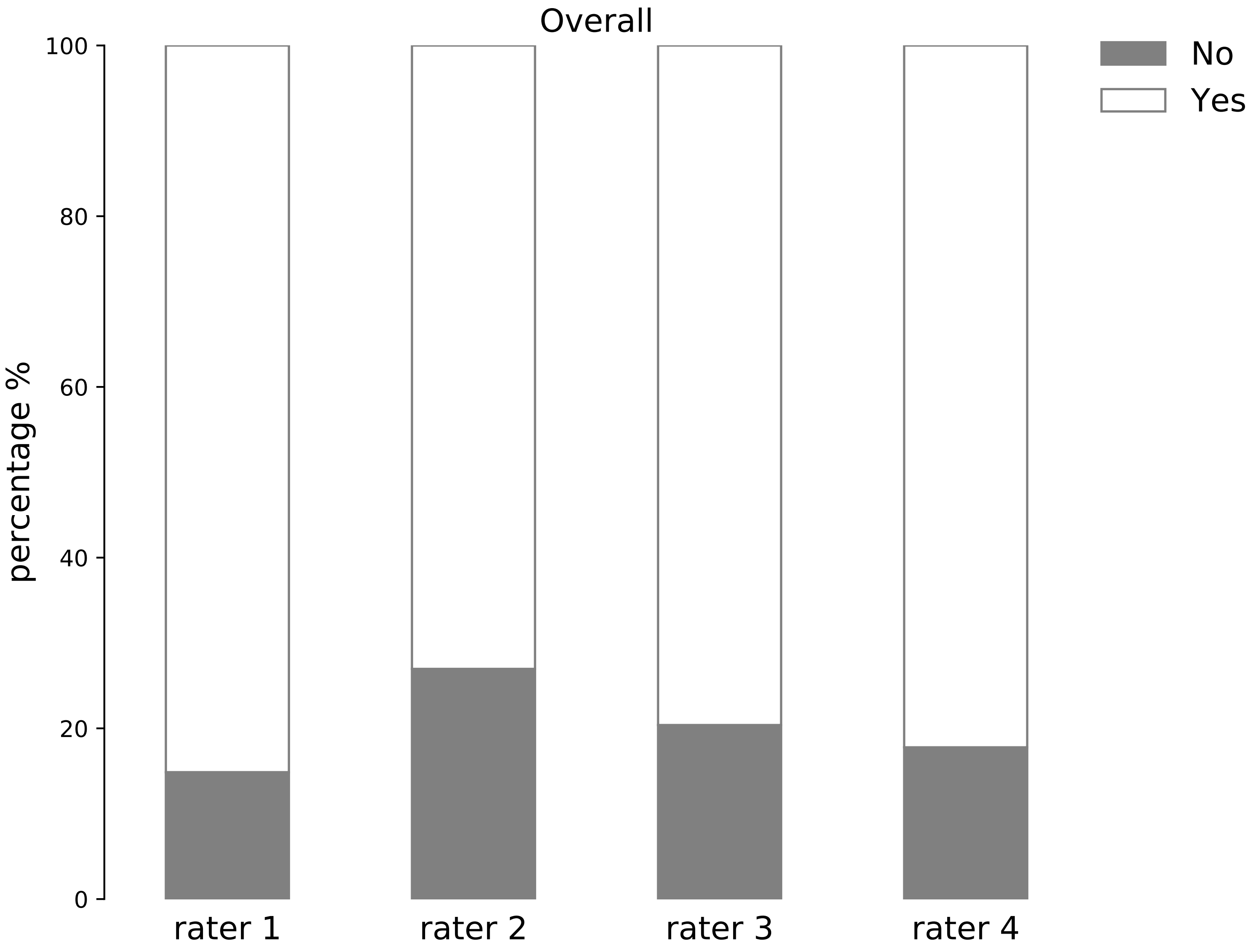

Question 3: Are p-values for all main analyses reported or implied?

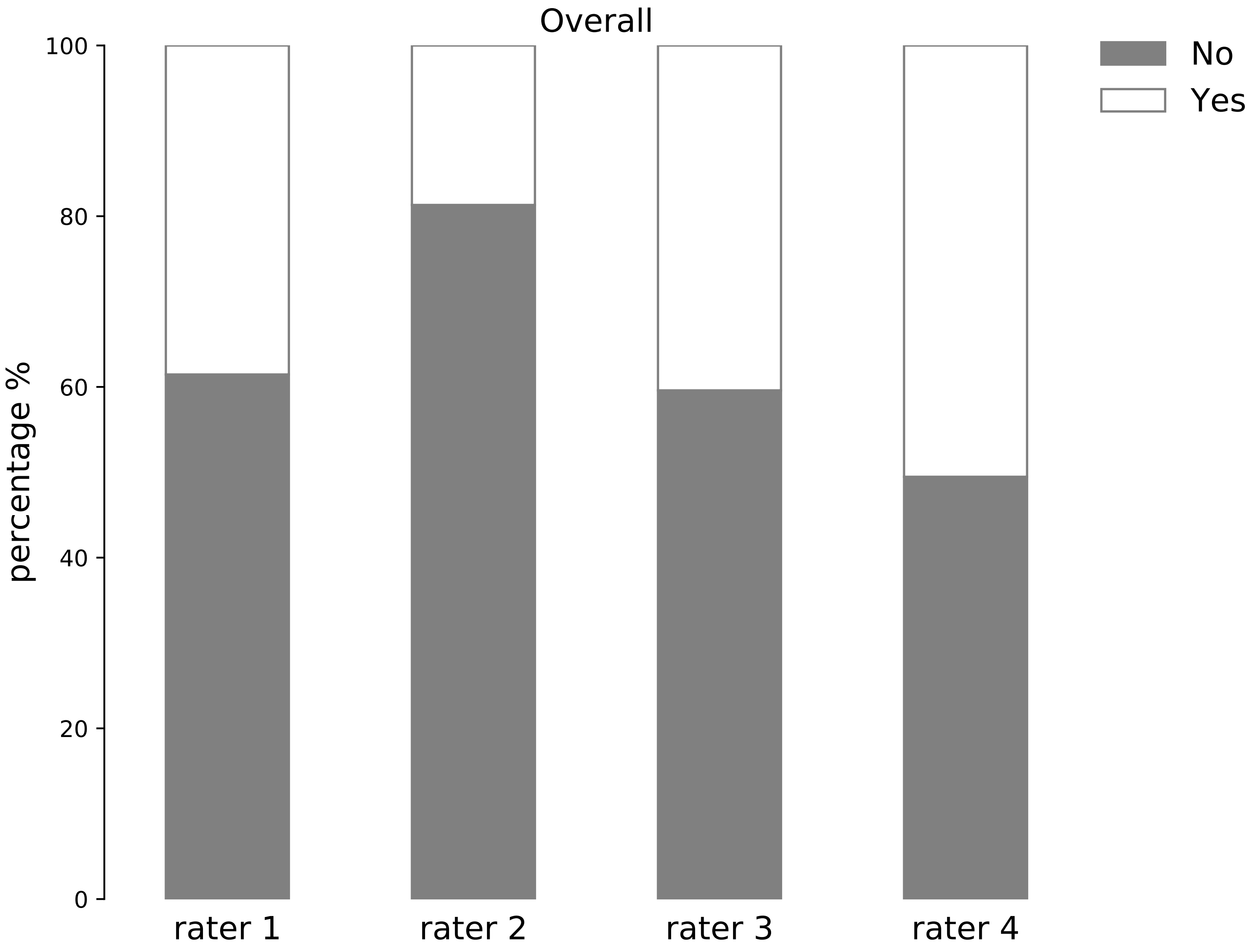

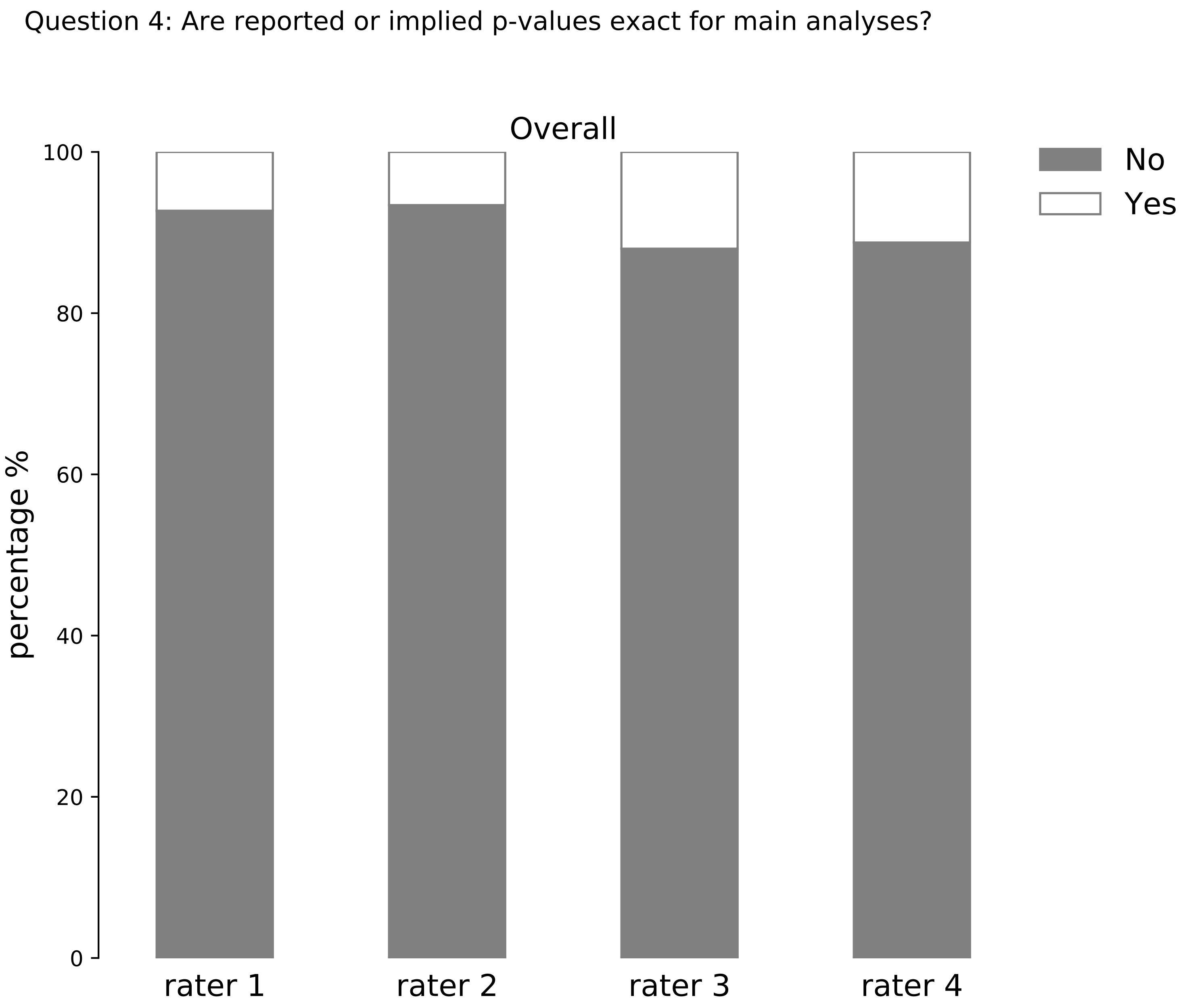
Question 4: Are reported or implied p-values exact for main analyses?
Overall
No
Yes
percentage %
100
80
60
40
20
0
rater 1
rater 2
rater 3
rater 4

Question 5: Are all reported or implied post-hoc p-values exact?

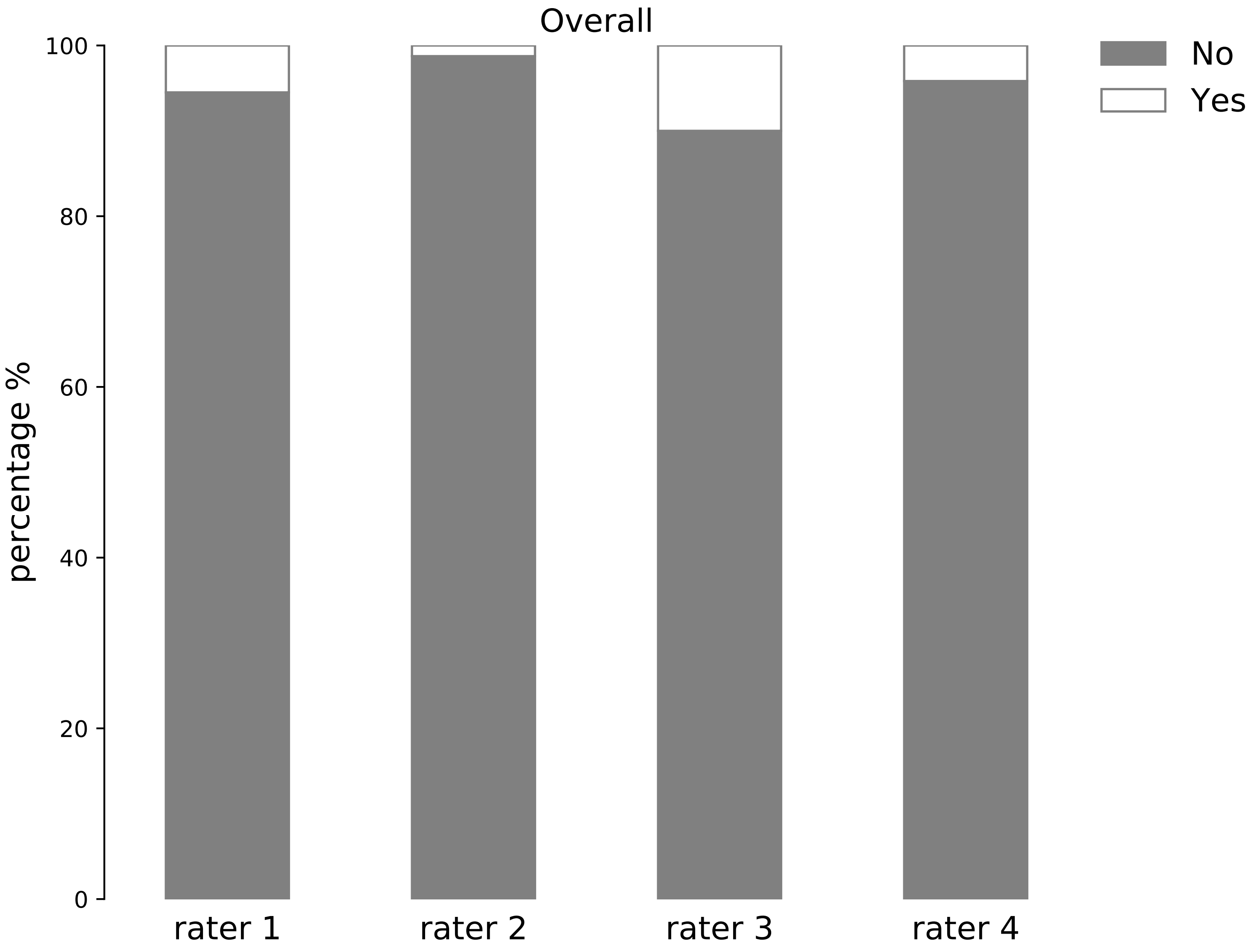

Question 6: Are all plotted measures that summarize variability defined?

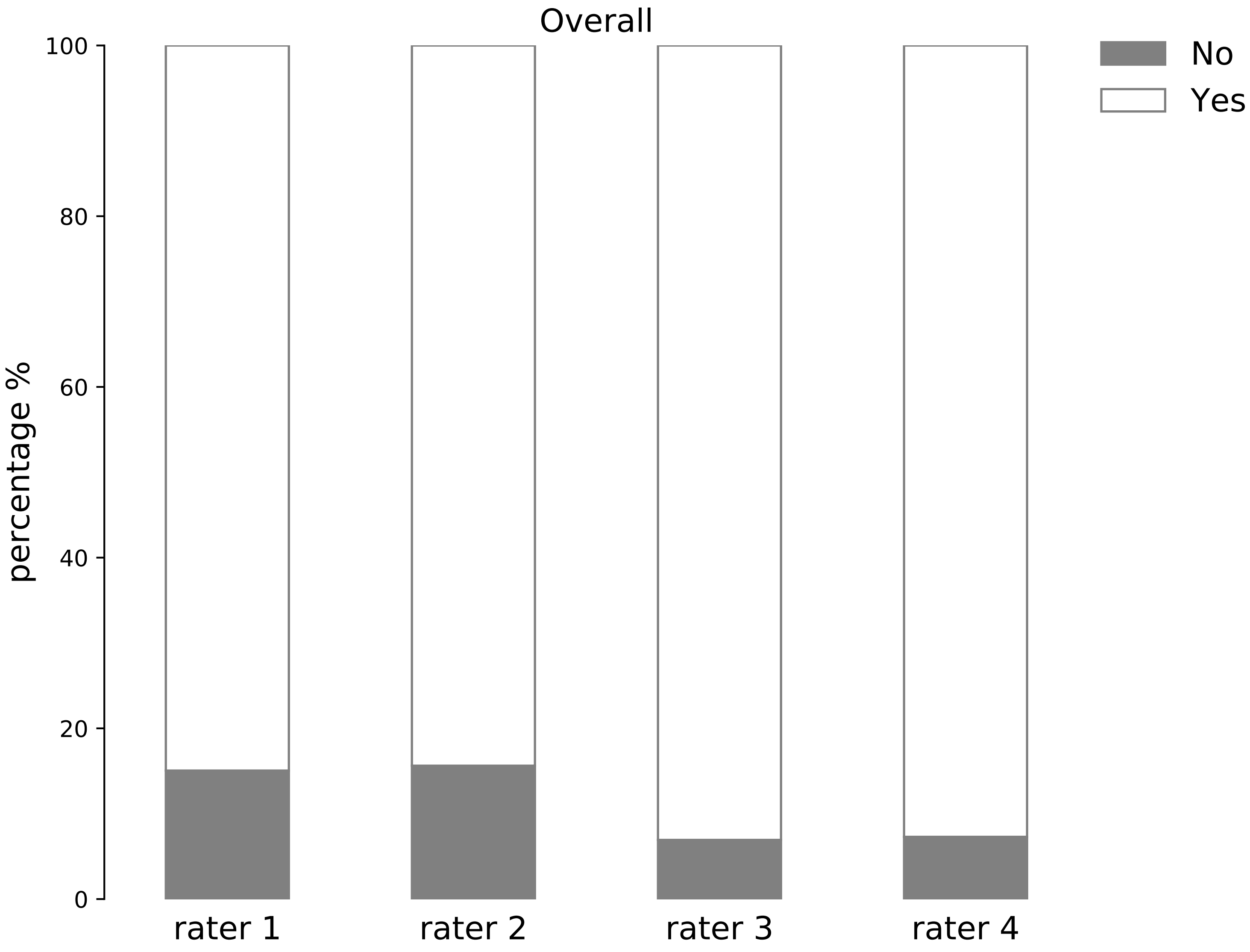

Question 7: Are any plotted measures that summarize variability SEM?

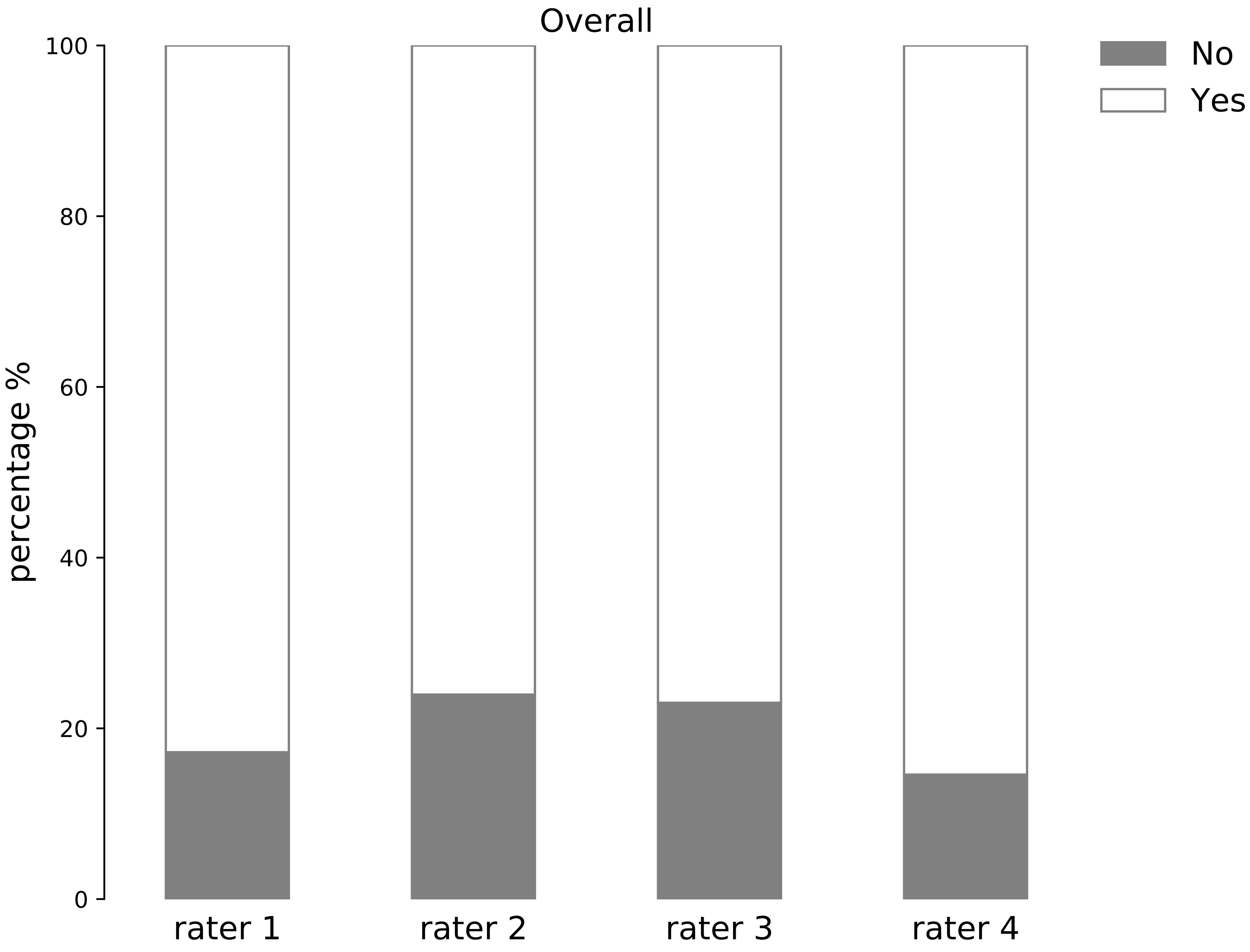

Question 8: For all figures that summarize data/variability, are raw data plotted?

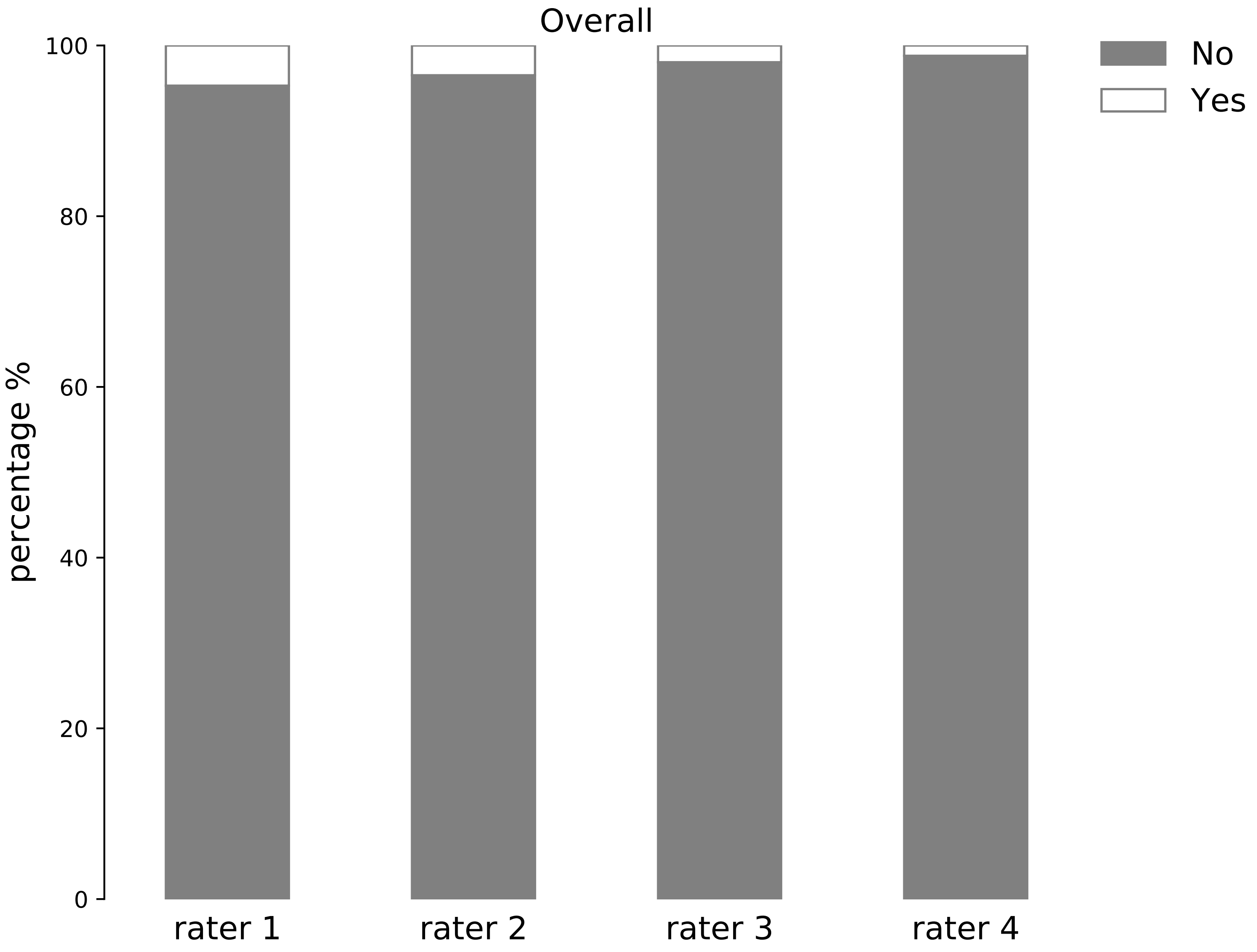

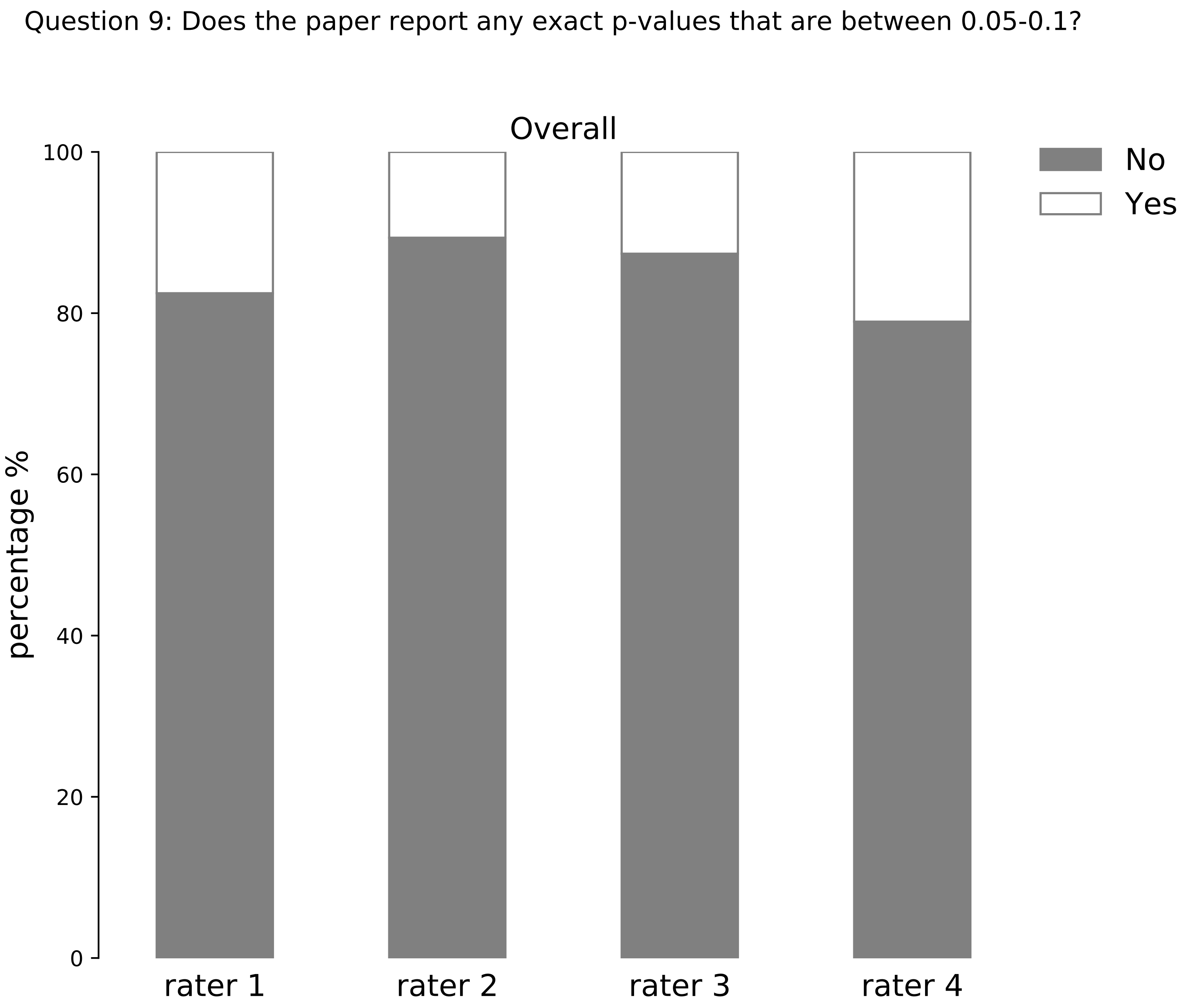

Question 9: Does the paper report any exact p-values that are between 0.05-0.1?
Overall
percentage %
0
20
40
60
80
100
rater 1
rater 2
rater 3
rater 4
No
Yes

Question 10: Are any p-values between 0.05-0.1 interpreted as trends/significance?

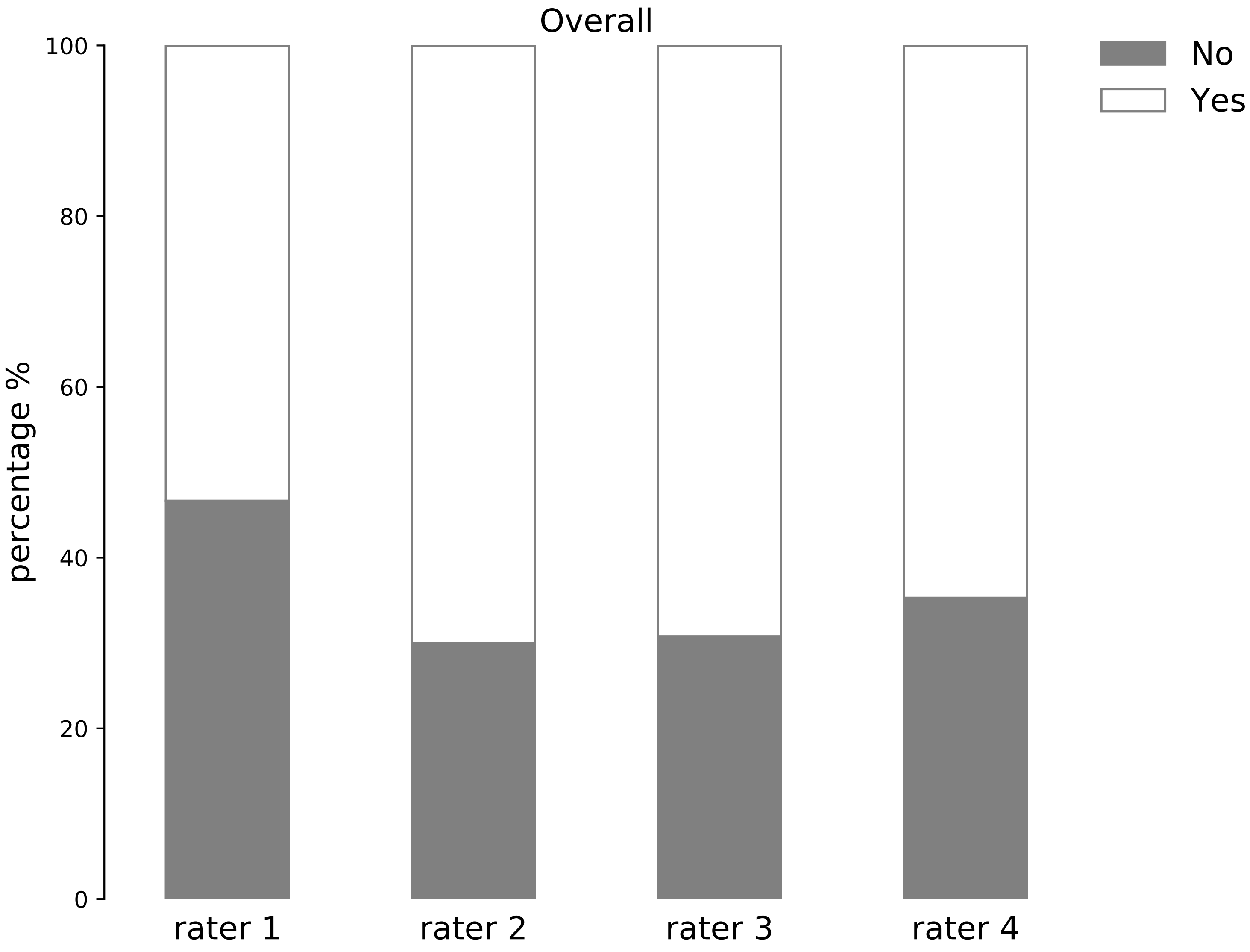

Supplement: S4 File — Comparison of audit results across raters indicates the scoring criteria were applied uniformly across raters. (PDF) [file pone.0202121.s004.pdf]
