# Supplementary material for: Poor statistical reporting, inadequate data presentation and spin persist despite editorial advice
Source: PLoS One. 2018 Aug 15;13(8):e0202121. doi: 10.1371/journal.pone.0202121 (PMC6093658; doi:10.1371/journal.pone.0202121)
Supplement: S5 File — Comparison of audit results for each year and journal. The British Journal of Pharmacology consistently had lower reporting rates of p-values for main analyses, and exact p-values for main analyses. (PDF) [file pone.0202121.s005.pdf]

Question 1: Are all written measures that summarize data variability defined?

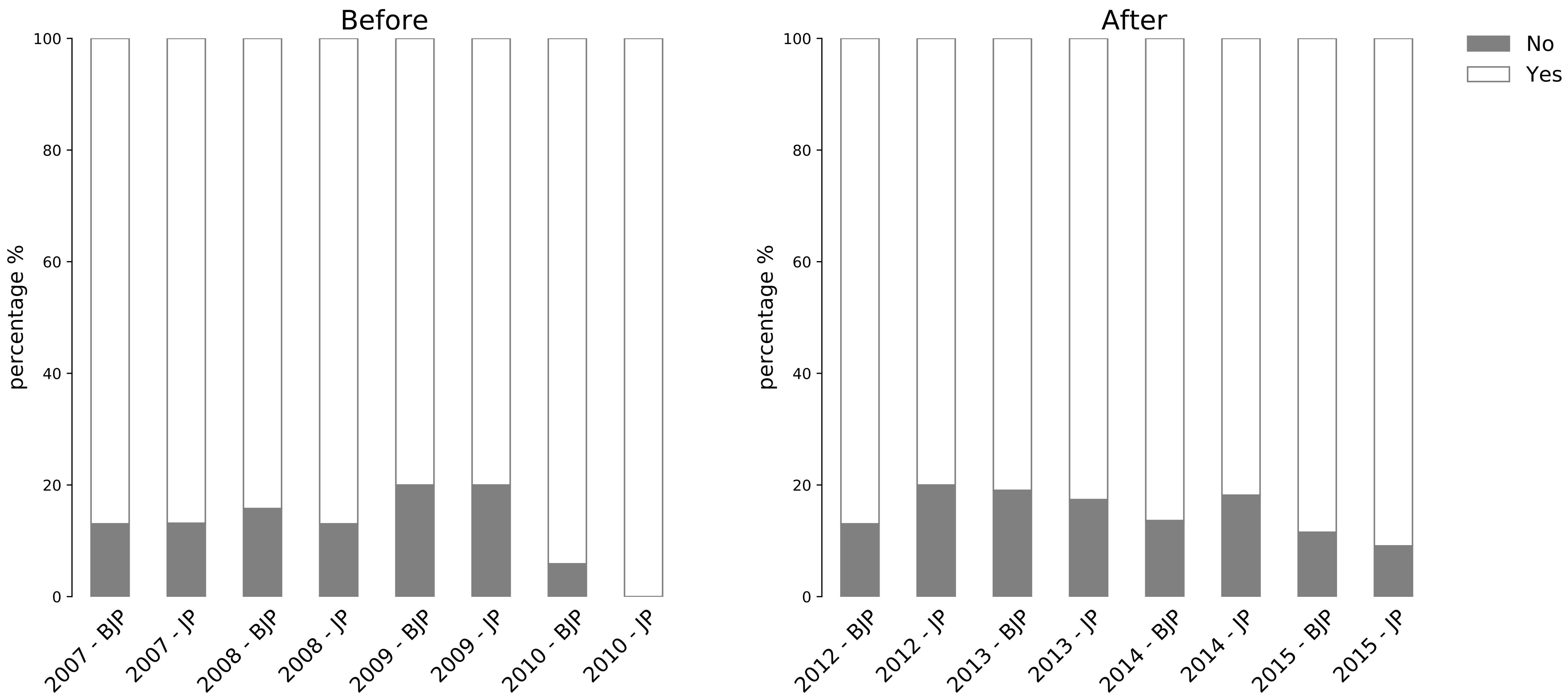

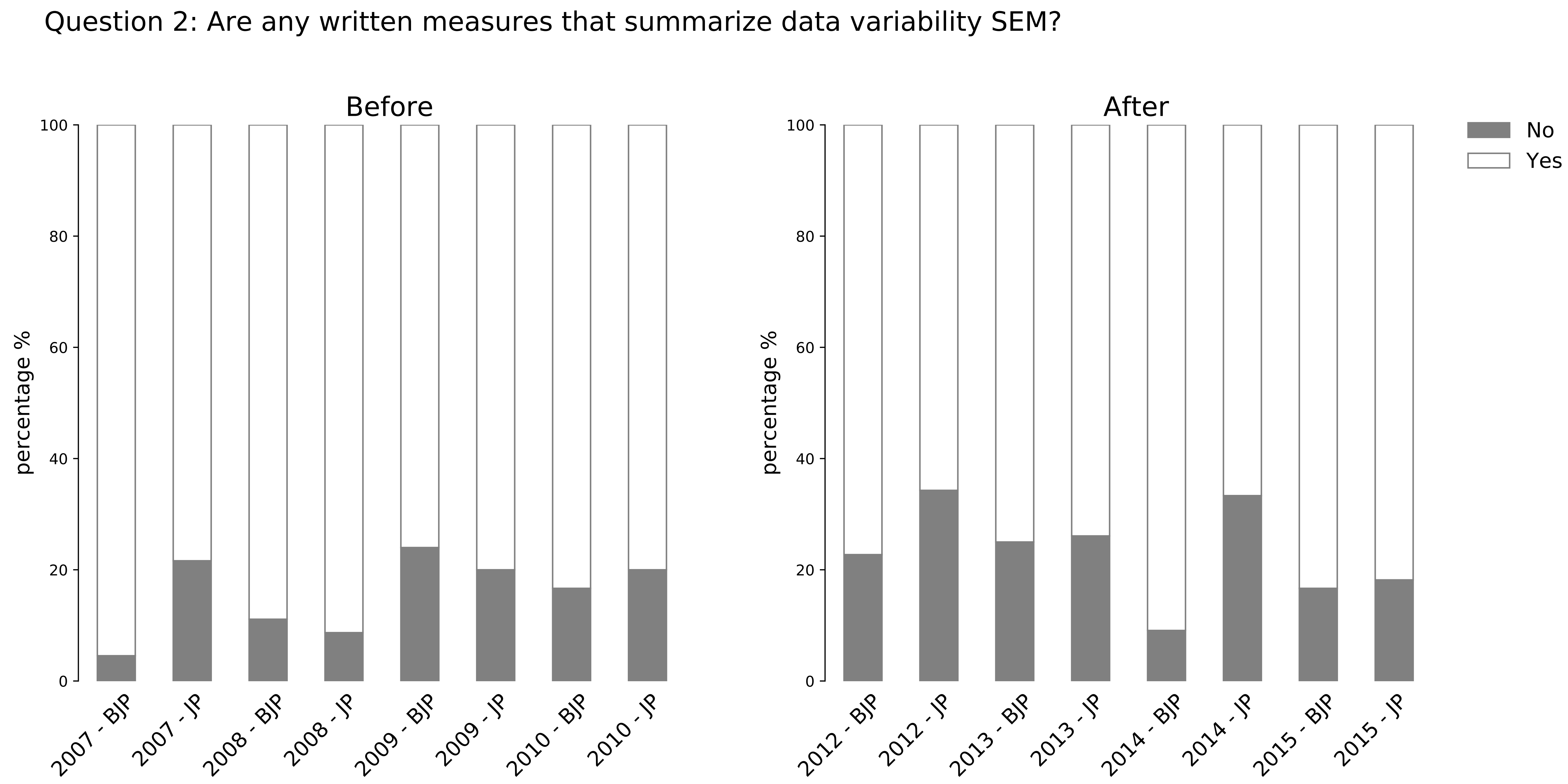
Question 2: Are any written measures that summarize data variability SEM?
Before
After
percentage %
percentage %
100
80
60
40
20
0
100
80
60
40
20
0
No
Yes
2007 - BJP
2007 - JP
2008 - BJP
2008 - JP
2009 - BJP
2009 - JP
2010 - BJP
2010 - JP
2012 - BJP
2012 - JP
2013 - BJP
2013 - JP
2014 - BJP
2014 - JP
2015 - BJP
2015 - JP

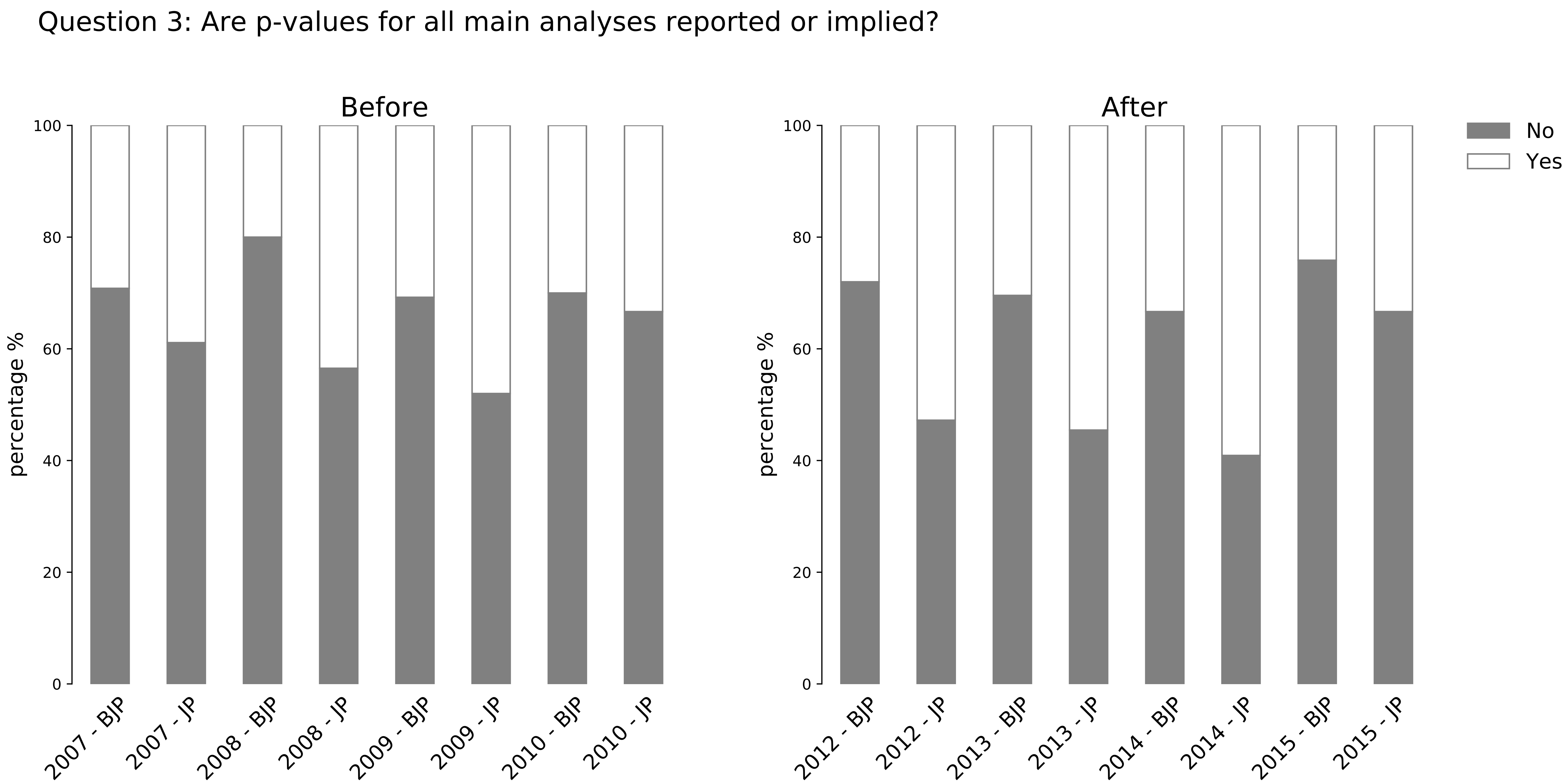

Question 3: Are p-values for all main analyses reported or implied?
Before
After
percentage %
0
20
40
60
80
100
2007 - BJP
2007 - JP
2008 - BJP
2008 - JP
2009 - BJP
2009 - JP
2010 - BJP
2010 - JP
2012 - BJP
2012 - JP
2013 - BJP
2013 - JP
2014 - BJP
2014 - JP
2015 - BJP
2015 - JP
No
Yes

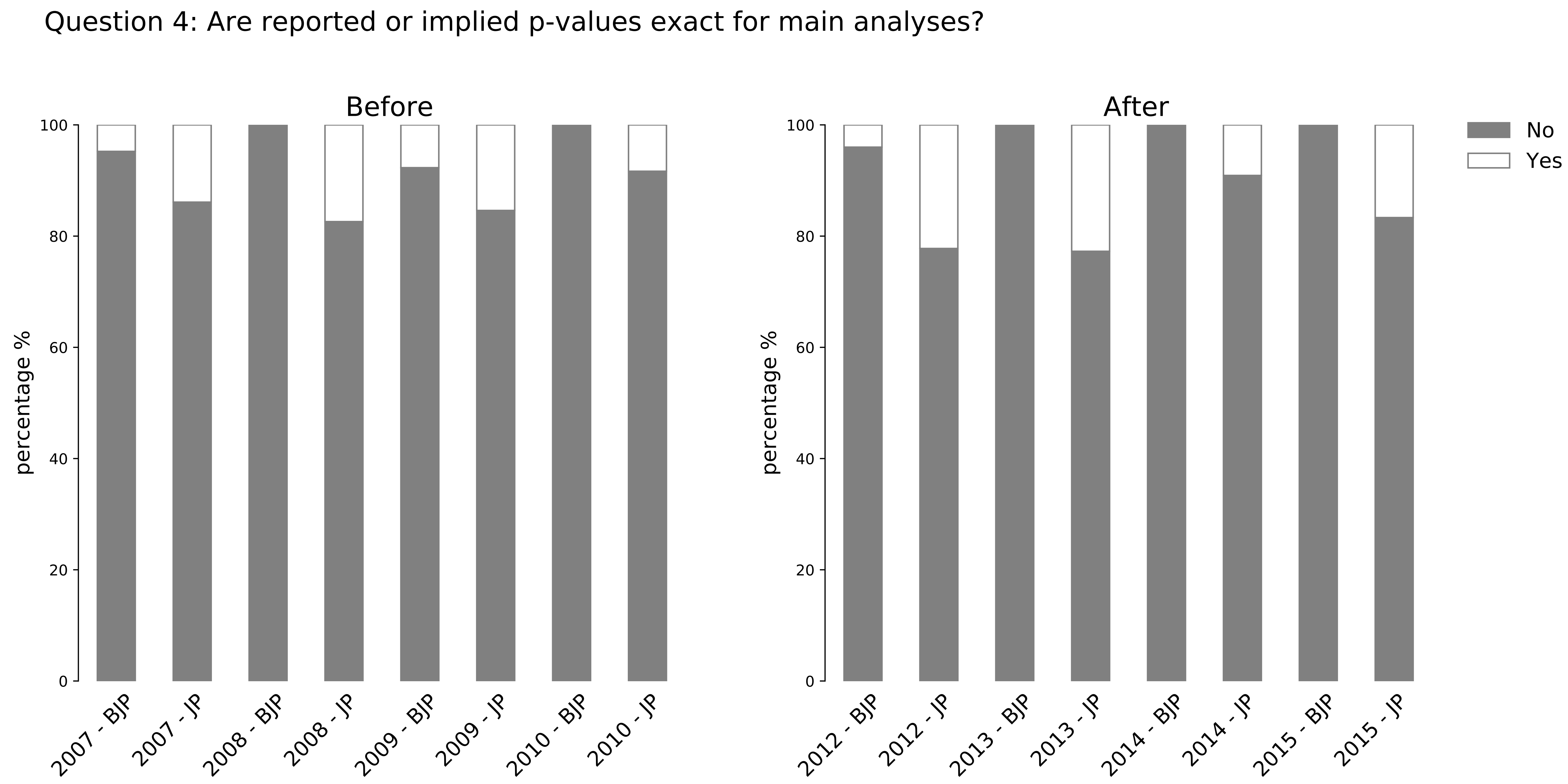

Question 4: Are reported or implied p-values exact for main analyses?
Before
After
percentage %
0
20
40
60
80
100
2007 - BJP
2007 - JP
2008 - BJP
2008 - JP
2009 - BJP
2009 - JP
2010 - BJP
2010 - JP
2012 - BJP
2012 - JP
2013 - BJP
2013 - JP
2014 - BJP
2014 - JP
2015 - BJP
2015 - JP
No
Yes

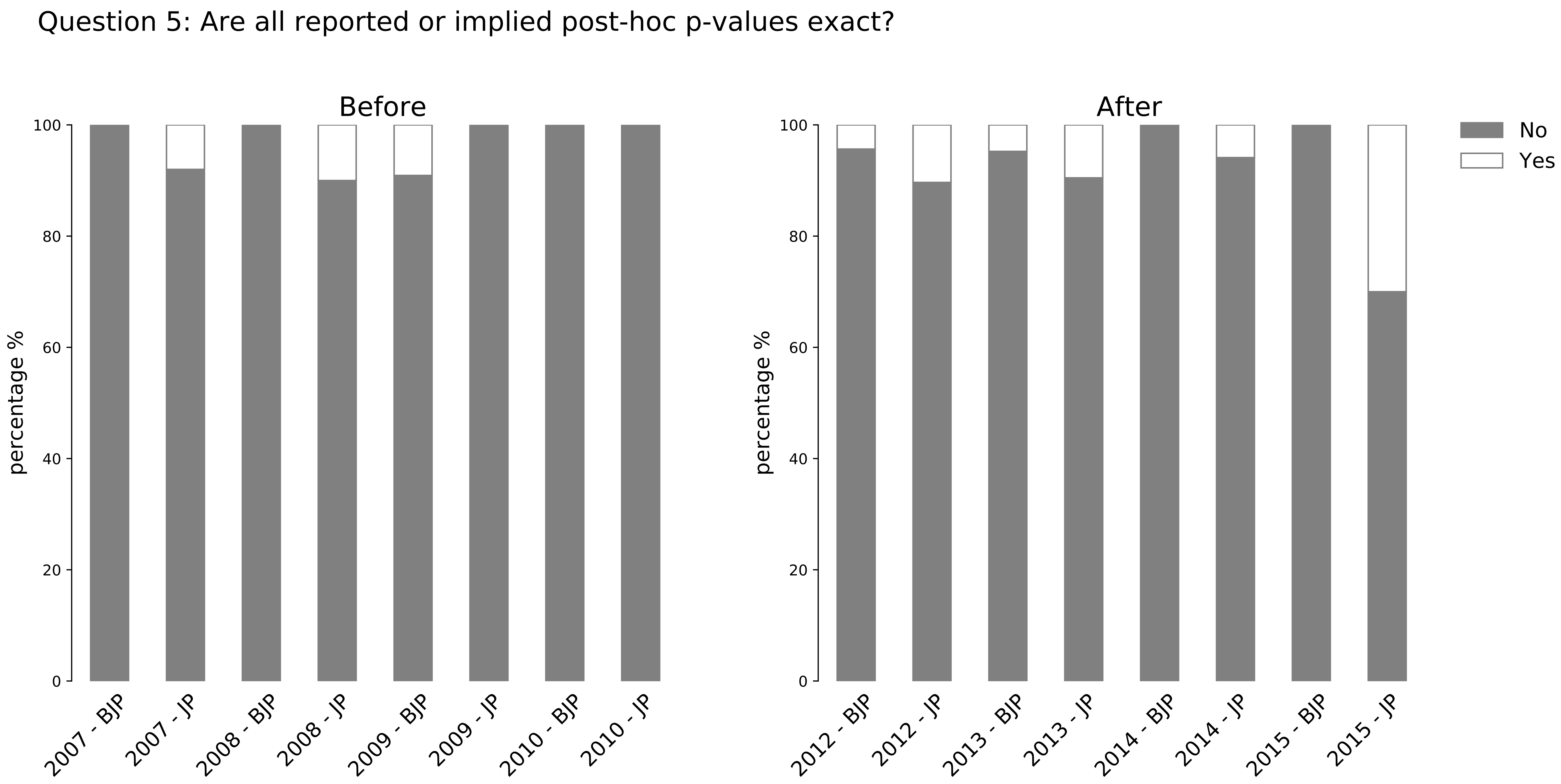
Question 5: Are all reported or implied post-hoc p-values exact?
Before
After
No
Yes
percentage %
0
20
40
60
80
100
2007 - BJP
2007 - JP
2008 - BJP
2008 - JP
2009 - BJP
2009 - JP
2010 - BJP
2010 - JP
2012 - BJP
2012 - JP
2013 - BJP
2013 - JP
2014 - BJP
2014 - JP
2015 - BJP
2015 - JP

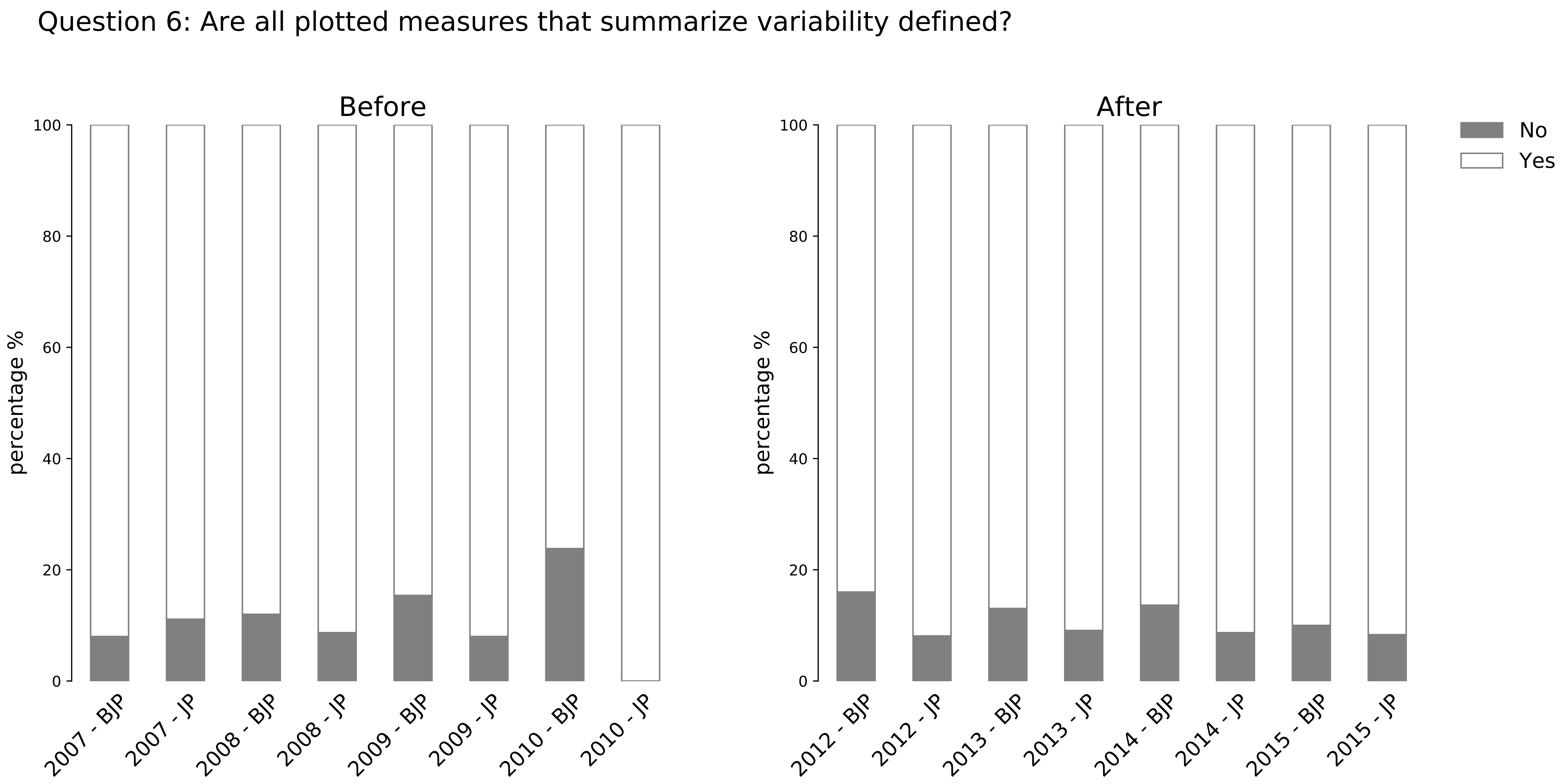

Question 6: Are all plotted measures that summarize variability defined?
Before
After
percentage %
percentage %
0
20
40
60
80
100
No
Yes
2007 - BJP
2007 - JP
2008 - BJP
2008 - JP
2009 - BJP
2009 - JP
2010 - BJP
2010 - JP
2012 - BJP
2012 - JP
2013 - BJP
2013 - JP
2014 - BJP
2014 - JP
2015 - BJP
2015 - JP

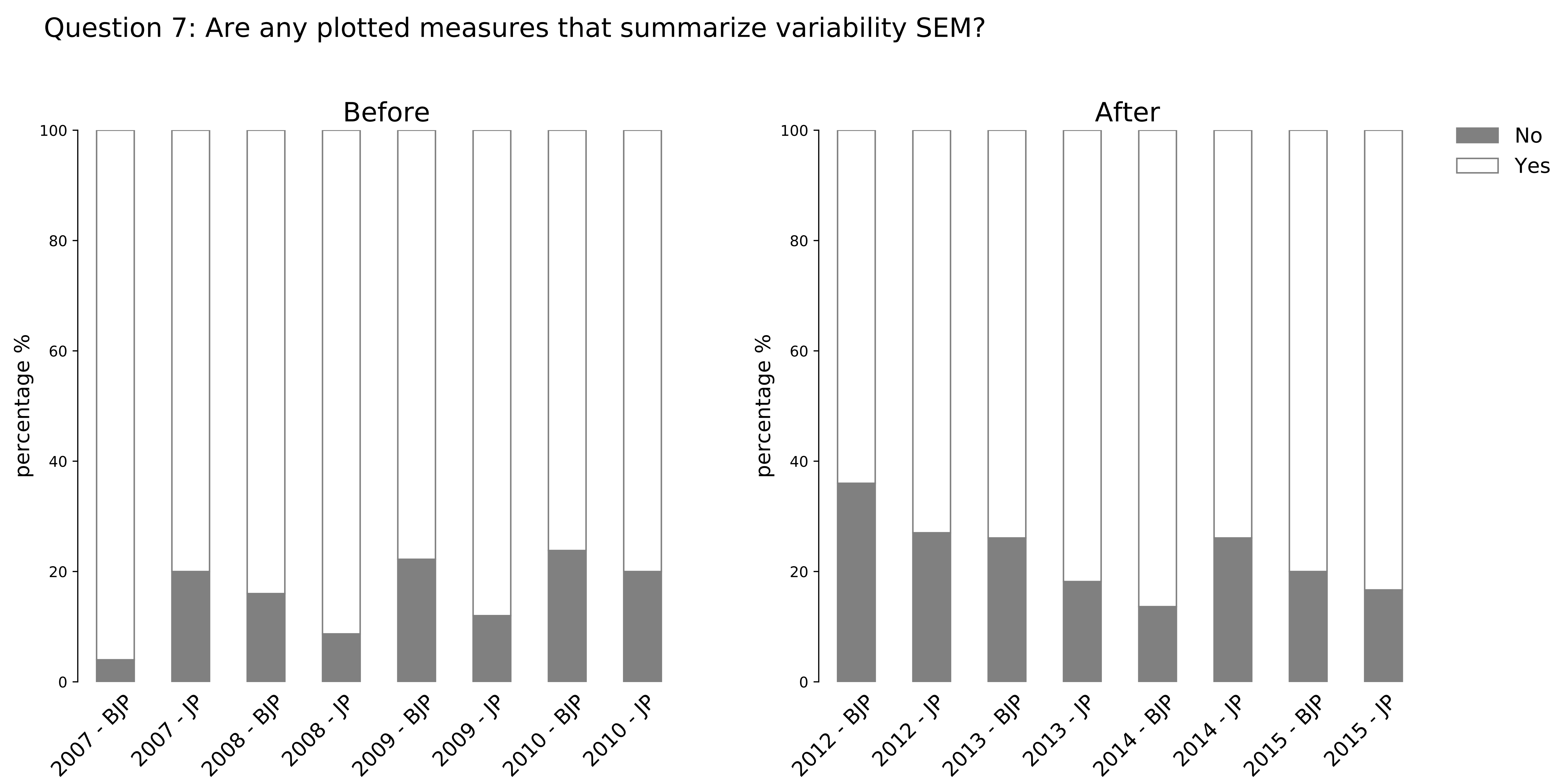

Question 7: Are any plotted measures that summarize variability SEM?
Before
After
percentage %
0
20
40
60
80
100
2007 - BJP
2007 - JP
2008 - BJP
2008 - JP
2009 - BJP
2009 - JP
2010 - BJP
2010 - JP
2012 - BJP
2012 - JP
2013 - BJP
2013 - JP
2014 - BJP
2014 - JP
2015 - BJP
2015 - JP
No
Yes

Question 8: For all figures that summarize data/variability, are raw data plotted?

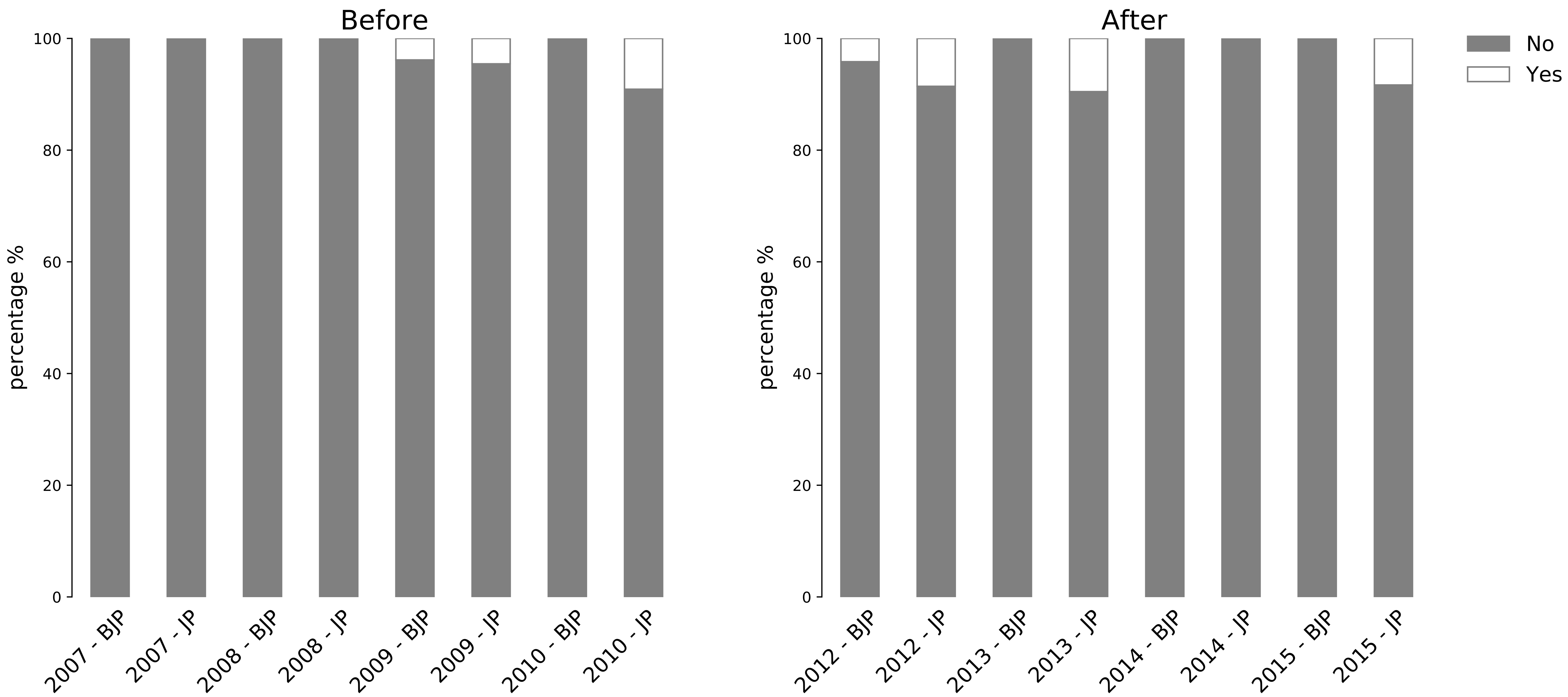

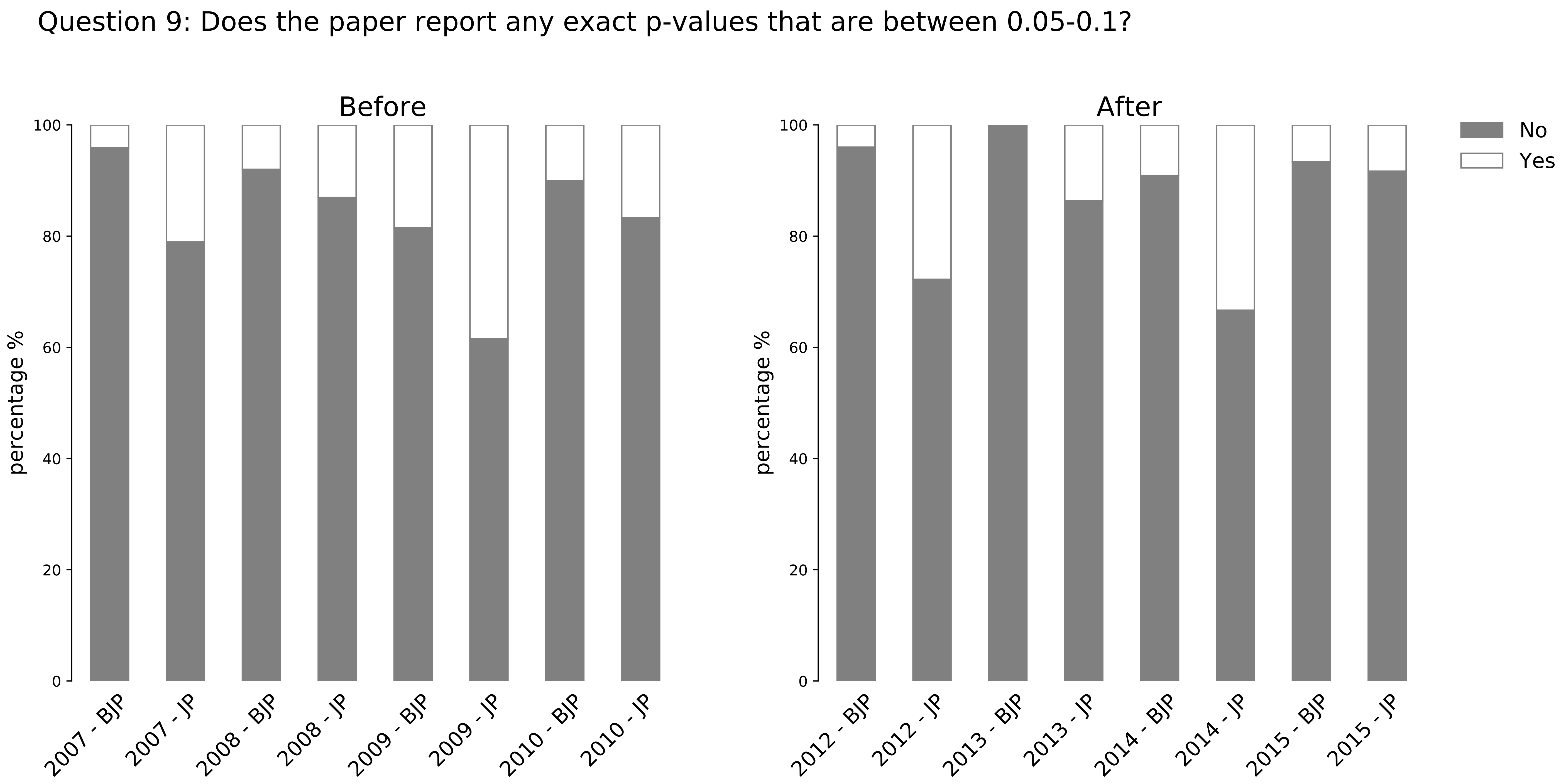
Question 9: Does the paper report any exact p-values that are between 0.05-0.1?
Before
After
percentage %
percentage %
0
20
40
60
80
100
2007 - BJP
2007 - JP
2008 - BJP
2008 - JP
2009 - BJP
2009 - JP
2010 - BJP
2010 - JP
2012 - BJP
2012 - JP
2013 - BJP
2013 - JP
2014 - BJP
2014 - JP
2015 - BJP
2015 - JP
No
Yes

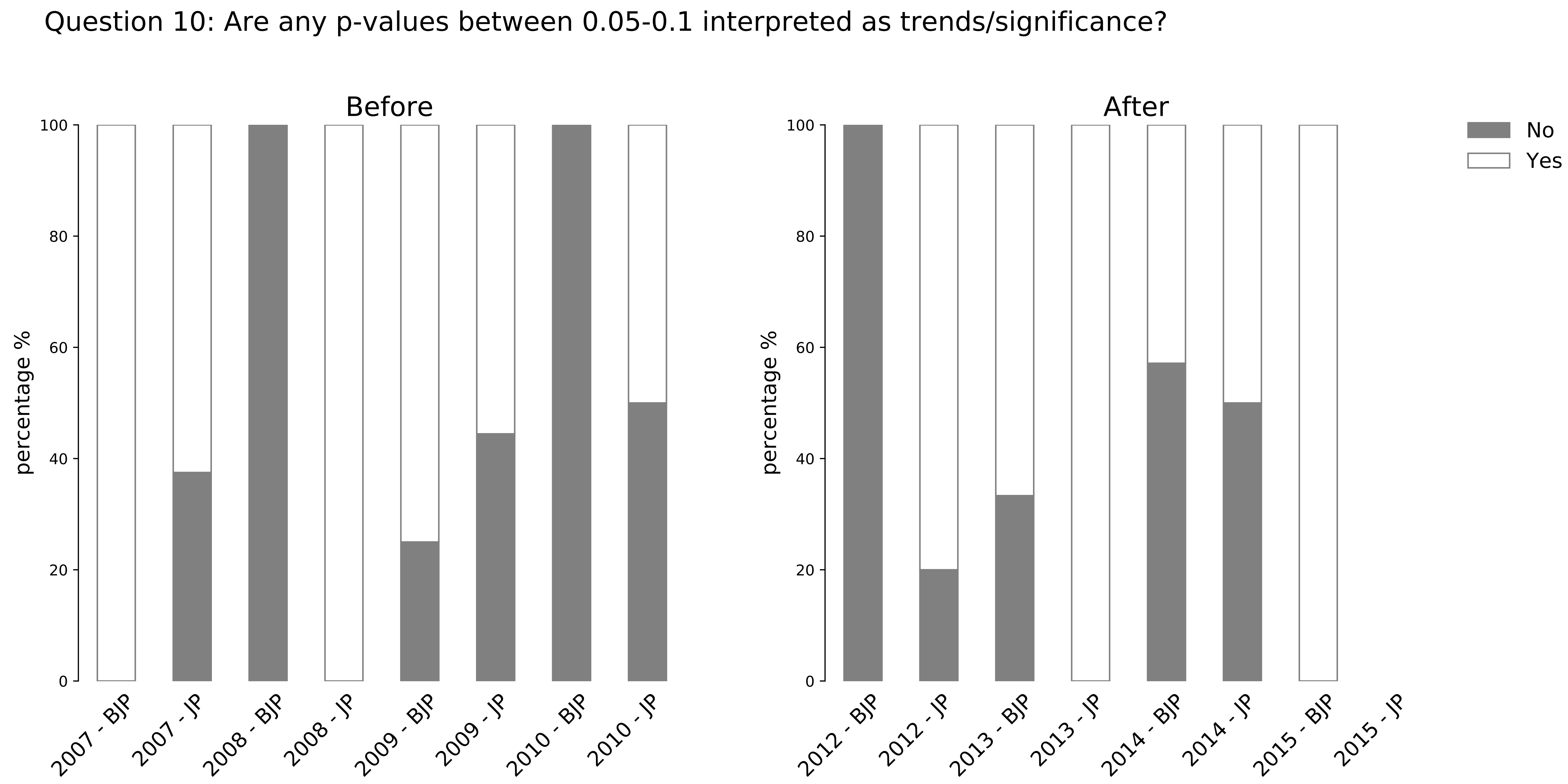
Question 10: Are any p-values between 0.05-0.1 interpreted as trends/significance?
Before
After
No
Yes
percentage %
0
20
40
60
80
100
2007 - BJP
2007 - JP
2008 - BJP
2008 - JP
2009 - BJP
2009 - JP
2010 - BJP
2010 - JP
2012 - BJP
2012 - JP
2013 - BJP
2013 - JP
2014 - BJP
2014 - JP
2015 - BJP
2015 - JP
